# Adjuvant mitotane versus surveillance in low-grade, localised adrenocortical carcinoma (ADIUVO): an international, multicentre, open-label, randomised, phase 3 trial and observational study

**DOI:** 10.1016/S2213-8587(23)00193-6

**Published:** 2023-10

**Authors:** Massimo Terzolo, Martin Fassnacht, Paola Perotti, Rossella Libé, Darko Kastelan, André Lacroix, Wiebke Arlt, Harm Reinout Haak, Paola Loli, Bénédicte Decoudier, Helene Lasolle, Marcus Quinkler, Magalie Haissaguerre, Olivier Chabre, Philippe Caron, Antonio Stigliano, Roberta Giordano, Maria Chiara Zatelli, Irina Bancos, Maria Candida Barisson Villares Fragoso, Letizia Canu, Michaela Luconi, Soraya Puglisi, Vittoria Basile, Giuseppe Reimondo, Matthias Kroiss, Felix Megerle, Stefanie Hahner, Otilia Kimpel, Tina Dusek, Svenja Nölting, Isabelle Bourdeau, Vasileios Chortis, Madeleine Hester Ettaieb, Deborah Cosentini, Salvatore Grisanti, Eric Baudin, Paola Berchialla, Francesca Bovis, Maria Pia Sormani, Paolo Bruzzi, Felix Beuschlein, Jerome Bertherat, Alfredo Berruti

**Affiliations:** a Internal Medicine, Department of Clinical and Biological Sciences, San Luigi Gonzaga Hospital, University of Turin, Turin, Italy; b Statistical Unit, Department of Clinical and Biological Sciences, San Luigi Gonzaga Hospital, University of Turin, Turin, Italy; c Department of Internal Medicine I, Division of Endocrinology and Diabetes, University Hospital, University of Würzburg, Würzburg, Germany; d Comprehensive Cancer Center Mainfranken, University of Würzburg, Würzburg, Germany; e Rare Cancer Network COMETE Cancer, Hôpital Cochin, Paris, France; f Department of Endocrinology University Hospital Zagreb, Zagreb, Croatia; g Service d'Endocrinologie, Département de Médecine, Centre Hospitalier de l'Universite de Montréal, Montreal, QC, Canada; h Institute of Metabolism and Systems Research, University of Birmingham, Birmingham, UK; i MRC London Institute of Medical Sciences and Faculty of Medicine, Imperial College London, London, UK; j Department of Internal Medicine, Maxima Medisch Centrum, Eindhoven, Netherlands; k CAPHRI School for Public Health and Primary Care, Ageing and Long-Term Care, Maastricht University, and Department of Internal Medicine, Division of General Internal Medicine, Maastricht University Medical Centre+, Maastricht, Netherlands; l Division of Endocrinology, Niguarda Cà Granda Hospital, Milan, Italy; m Centre Hospitalier Universitaire de Reims, Service d'Endocrinologie-Diabètologie-Nutrition, Reims, France; n Federation d'Endocrinologie, Hospices Civils de Lyon and University de Lyon, Lyon, France; o Endokrinologie in Charlottenburg, Berlin, Germany; p Department of Endocrinology and Endocrine Oncology, Haut Leveque Hospital, University Hospital of Bordeaux, France; q University Grenoble Alpes, Service d'Endocrinologie CHU Grenoble Alpes, Unité Mixte de Recherche INSERM-CEA-UGA UMR1036 38000 Grenoble Alpes, France; r Department of Endocrinology and Metabolic Diseases, Cardiovascular and Metabolic Unit, CHU Larrey, Toulouse, France; s Endocrinology, Department of Clinical and Molecular Medicine, Sant'Andrea University Hospital, Sapienza University of Rome, Rome, Italy; t Division of Endocrinology, Diabetology and Metabolism, Department of Biological and Clinical Sciences, University of Turin, Turin, Italy; u Section of Endocrinology and Internal Medicine, Department of Medical Sciences, University of Ferrara, Ferrara, Italy; v Division of Endocrinology, Diabetes, Metabolism and Nutrition, Mayo Clinic, Rochester, MN, USA; w Unidade de Suprarrenal, Disciplina de Endocrinologia e Metabologia do Hospital das Clinicas da Faculdade de Medicina da Universidade de São Paulo, Instituto do Cancer do Estado de São Paulo-ICESP, São Paulo, Brazil; x Department of Experimental and Clinical Biomedical Sciences, University of Florence, Florence, Italy; y Department of Endocrinology, Medizinische Klinik und Poliklinik IV, Klinikum der Universität München, Munich, Germany; z Department of Endocrinology, Diabetology and Clinical Nutrition, University Hospital Zurich and University of Zurich, Zurich, Switzerland; aa Oncology, Department of Medical and Surgical Specialties, Radiological Sciences, and Public Health Medical, ASST-Spedali Civili, University of Brescia, Brescia, Italy; ab Endocrine Oncology Gustave Roussy, Villejuif, France; ac Department of Health Sciences, University of Genoa, Genoa, Italy; ad Université Paris Cité, Institut Cochin, Inserm U1016, CNRS UMR8104, Service d'Endocrinologie, Hopital Cochin, APHP, Paris, France

## Abstract

**Background:**

Adjuvant treatment with mitotane is commonly used after resection of adrenocortical carcinoma; however, treatment remains controversial, particularly if risk of recurrence is not high. We aimed to assess the efficacy and safety of adjuvant mitotane compared with surveillance alone following complete tumour resection in patients with adrenocortical carcinoma considered to be at low to intermediate risk of recurrence.

**Methods:**

ADIUVO was a multicentre, open-label, parallel, randomised, phase 3 trial done in 23 centres across seven countries. Patients aged 18 years or older with adrenocortical carcinoma and low to intermediate risk of recurrence (R0, stage I–III, and Ki67 ≤10%) were randomly assigned to adjuvant oral mitotane two or three times daily (the dose was adjusted by the local investigator with the target of reaching and maintaining plasma mitotane concentrations of 14–20 mg/L) for 2 years or surveillance alone. All consecutive patients at 14 study centres fulfilling the eligibility criteria of the ADIUVO trial who refused randomisation and agreed on data collection via the European Network for the Study of Adrenal Tumors adrenocortical carcinoma registry were included prospectively in the ADIUVO Observational study. The primary endpoint was recurrence-free survival, defined as the time from randomisation to the first radiological evidence of recurrence or death from any cause (whichever occurred first), assessed in all randomly assigned patients by intention to treat. Overall survival, defined as time from the date of randomisation to the date of death from any cause, was a secondary endpoint analysed by intention to treat in all randomly assigned patients. Safety was assessed in all patients who adhered to the assigned regimen, which was defined by taking at least one tablet of mitotane in the mitotane group and no mitotane at all in the surveillance group. The ADIUVO trial is registered with ClinicalTrials.gov, NCT00777244, and is now complete.

**Findings:**

Between Oct 23, 2008, and Dec 27, 2018, 45 patients were randomly assigned to mitotane and 46 to surveillance alone. Because the study was discontinued prematurely, 5-year recurrence-free and overall survival are reported instead of recurrence-free and overall survival as defined in the protocol. 5-year recurrence-free survival was 79% (95% CI 67–94) in the mitotane group and 75% (63–90) in the surveillance group (hazard ratio 0·74 [95% CI 0·30–1·85]). Two people in the mitotane group and five people in the surveillance group died, and 5-year overall survival was not significantly different (95% [95% CI 89–100] in the mitotane group and 86% [74–100] in the surveillance group). All 42 patients who received mitotane had adverse events, and eight (19%) discontinued treatment. There were no grade 4 adverse events or treatment-related deaths.

**Interpretation:**

Adjuvant mitotane might not be indicated in patients with low-grade, localised adrenocortical carcinoma considering the relatively good prognosis of these patients, and no significant improvement in recurrence-free survival and treatment-associated toxicity in the mitotane group. However, the study was discontinued prematurely due to slow recruitment and cannot rule out an efficacy of treatment.

**Funding:**

AIFA, ENSAT Cancer Health F2-2010-259735 programme, Deutsche Forschungsgemeinschaft, Cancer Research UK, and the French Ministry of Health.

**Research in context**

**Evidence before this study**

Adrenocortical carcinoma is a rare malignancy, and prognosis is poor; however, heterogeneity in prognosis between different groups of patients has been increasingly recognised. Adjuvant mitotane has been extensively applied following tumour resection in most patients with adrenocortical carcinoma on the basis of retrospective studies suggesting that mitotane treatment after tumour removal prolongs the time to recurrence. Given some conflicting findings from other studies and additional challenges associated with mitotane administration, which entails a cumbersome drug regimen, complex hormone replacement, need of careful monitoring, and potential toxicity, the utility of adjuvant mitotane treatment for all patients with adrenocortical carcinoma was questionable. Due to the absence of randomised studies, we recognised that a phase 3 trial was needed to establish whether this therapeutic intervention could benefit patients. In 2008, we initiated the ADIUVO trial with the support of the European Network for the Study of Adrenal Tumors (ENS@T). During preparation of the European Society of Endocrinology and ENS@T 2018 Clinical Guidelines on Management of Adrenocortical Carcinoma, a comprehensive literature search of PubMed, the NHS Economic Evaluation Database, and the Cochrane Database of Systematic Reviews, and the Database of Abstracts of Reviews of Effects was performed, from Jan 1, 1986, to Feb 28, 2018, for all systematic reviews and studies that had assessed adjuvant therapies in adult patients with adrenocortical carcinoma. The search terms were “adrenal”/”adrenocortical cancer”/”carcinoma”/”neoplasm”, “adjuvant”/”additional”/”post-operative therapy”, “mitotane”/”o,p’-DDD”, “recurrence”/”relapse”, “overall survival”. We searched for studies published in English. This search revealed only six retrospective studies that assessed the effect of adjuvant mitotane on recurrence and five studies on survival, including a total of 322 patients on mitotane and 718 patients on surveillance alone. In a meta-analysis, the pooled hazard ratio for recurrence was 0·7 (95% CI 0·5–1·1) and for mortality 0·7 (95% CI 0·5–0·9). We updated our initial literature search (to include literature from March 1, 2018, to March 31, 2023) using the same search terms and language restriction, and identified one additional meta-analysis, three new retrospective studies, and no randomised trials. The results were in line with the findings described above.

**Added value of this study**

Despite the rarity of adrenocortical carcinoma, we were able to prospectively follow up almost 200 patients who were included in the interventional trial (ADIUVO) and the accompanying observational study (ADIUVO Observational); a size rivalled only by the FIRM-ACT trial on advanced adrenocortical carcinoma. ADIUVO is the first randomised trial in the postoperative adjuvant setting for patients with adrenocortical carcinoma. The study indicates that it is possible to identify, with a simple and widely available prognostication scheme based on adrenocortical carcinoma stage, resection status, and Ki67 assessment, a subset of patients whose prognosis is much better than anticipated. The estimated 5-year recurrence-free survival in our patient cohorts approximated 75%, whereas it was previously reported to be less than 40% in retrospective patient cohorts. We showed prospectively that adjuvant mitotane treatment is associated with inherent toxicity, leading to a rate of permanent discontinuation of 19% even in the participating expert centres. Moreover, we found that the quality of life of patients with adrenocortical carcinoma is affected by adjuvant treatment with mitotane. The study did not reach the planned power because recruiting was hampered by the fact that only a minority of patients referred after surgery could be considered as having a low risk of recurrence; therefore, an effect of mitotane cannot be definitively ruled out. However, given the relatively good prognosis of our patient cohort even with surveillance alone, and the challenges associated with adjuvant mitotane treatment, the ADIUVO findings do not support a routine use of adjuvant mitotane after complete removal of a low-grade, localised adrenocortical carcinoma.

**Implications of all the available evidence**

Our study has an immediate impact on the management of patients with localised adrenocortical carcinoma, since we show for the first time in a prospective way that patients with low risk of recurrence do exist, and can be identified easily following surgery. Such patients can be spared a challenging and potentially toxic treatment with mitotane and be adequately managed with active surveillance alone. The present evidence improves our capability of providing a more personalised adjuvant treatment. Our findings do not apply to patients with adrenocortical carcinoma at standard or high risk of recurrence, for which adjuvant mitotane remains recommended on the basis of previous evidence.

## Introduction

Adrenocortical carcinoma is a rare tumour arising from the cortex of the adrenal gland, with few therapeutic options.^1^ Surgery is considered the best treatment for non-metastatic adrenocortical carcinoma; however, retrospective studies reported that as many as 40–70% of adrenocortical carcinomas eventually recur even after complete resection.^2^, ^3^, ^4^, ^5^ The rarity of the disease, the lack of promising molecular targets, and the scarce resources devoted to the implementation of treatment strategies have hindered progress in medical therapy of adrenocortical carcinoma.^6^

On the basis of this evidence, the development of an effective adjuvant treatment is an unmet clinical need. Post-surgical adjuvant mitotane treatment was introduced into clinical practice more than 50 years ago.^7^ However, the relevant literature is filled with small, retrospective, observational studies with conflicting results.^6^ In the absence of data from randomised trials, the best evidence in favour of adjuvant mitotane comes from an observational study in which we tried to minimise confounding and bias by comparing two management strategies (mitotane treatment *vs* surveillance alone) that were applied following the local treatment policy of each centre and not based on the characteristics of patients.^8^

Following that study and subsequent recommendations of international guidelines to use adjuvant mitotane in most patients,^1^, ^9^ mitotane has been increasingly adopted in expert centres. Although a randomised trial would formally be required to establish a treatment-related clinical benefit, assignment of patients at high risk of recurrence to surveillance alone was considered unethical. Thus, the ADIUVO study was designed to evaluate the efficacy and safety of adjuvant mitotane compared with surveillance alone following complete tumour resection in patients with adrenocortical carcinoma considered to be at low to intermediate risk of recurrence. In this subset of patients, treatment is considered to be more controversial than in patients with high risk of recurrence.^10^, ^11^ Inclusion criteria were based on the assumption that patient's risk of recurrence could be stratified on the basis of the proliferation rate,^2^, ^5^, ^12^, ^13^, ^14^ with a Ki67 level at 10% differentiating best between patients at low risk and those at high risk.^15^ Given the slow recruitment rate and the number of patients who refused randomisation, a prospective, observational study recruiting patients eligible for but not randomly assigned in the interventional study was carried out in parallel to the randomised trial.

## Methods

### Study design and participants

ADIUVO is a public-funded, investigator-initiated, randomised, controlled, open-label, trial that was conducted in seven countries at 23 centres participating in the European Network for the Study of Adrenal Tumors (ENS@T; appendix p 14). Key inclusion criteria were age 18 years or older, histologically confirmed diagnosis of adrenocortical carcinoma, ENS@T stage I–III,^16^ microscopically complete resection (R0 surgery); Ki67 proliferation index of 10% or less, no evidence of disease at postoperative imaging (thoracic, abdominal, and pelvic contrast-enhanced CT or MRI), and Eastern Cooperative Oncology Group performance status of 0–2. Key exclusion criteria were time between primary surgery and randomisation of 3 months or more, repeated surgery for recurrent adrenocortical carcinoma, second malignancy, previous or current treatment with mitotane or other antineoplastic drugs for adrenocortical carcinoma, and previous radiotherapy for adrenocortical carcinoma. The complete list of inclusion and exclusion criteria are in the appendix (pp 52–53). The protocol and the statistical analysis plan are available in the appendix (pp 35–138). The study conformed to the principles of the Declaration of Helsinki and the Good Clinical Practice Guidelines and was approved by the ethics committee at each study centre (approval at the coordinating centre on Jan 21, 2008, reference number 153/INT). All patients provided written informed consent to participate.

### Randomisation and masking

After consent, patients were randomly assigned (1:1) in a parallel group design to receive either adjuvant mitotane or to active surveillance alone, and stratified for stage (I–II *vs* III) using automated computer-generated randomisation with the technique of randomly permuted balanced blocks with random block size. A centralised randomisation procedure was run online on the ADIUVO website throughout the study. After registering the patient and confirming inclusion and exclusion criteria, the patient was randomly assigned through logging on to the web system. The procedure was completely concealed to researchers and accessible only to the clinical research coordinator. The study was conducted unmasked. Placebo was not used because patients on mitotane require concomitant glucocorticoid replacement to prevent adrenal insufficiency.^1^

All consecutive patients at 14 study centres fulfilling the eligibility criteria of the ADIUVO trial who refused randomisation and agreed on data collection via the ENS@T adrenocortical carcinoma registry were included prospectively in the ADIUVO Observational study (appendix pp 29–30). These patients were assigned to adjuvant mitotane or surveillance alone according to patients’ choices and were followed up in an identical manner to the patients included in the ADIUVO trial.

### Procedures

Mitotane (Lysodren) was provided by HRA Pharma (Paris, France) and was administered orally starting at latest 7 days after randomisation. The mitotane dose was adjusted by the local investigator with the target of reaching and maintaining plasma mitotane concentrations of 14–20 mg/L because these concentrations have been associated with therapeutic efficacy.^17^, ^18^ A summary of the studies that assessed the prognostic value of plasma mitotane concentrations is in the appendix (p 24). When these concentrations were not attained, the patient received the maximal tolerated daily dose of mitotane. Measurement of plasma mitotane concentrations was performed in a central laboratory through the Lysosafe service. Lysosafe is a service provided without charge by HRA Pharma for the monitoring of mitotane plasma concentrations in patients on treatment. Mitotane concentrations were measured every 3 months with additional samplings done at the discretion of the local investigator. Patients treated with mitotane received concomitant glucocorticoid replacement. As the individually required dosage of this replacement is variable depending on several factors, including patient age, bodyweight, and clinical circumstances, the exact regimen was selected by the local investigator. Other therapies that were deemed as necessary for the supportive care and safety of the patients were also allowed according to patient needs at the discretion of the treating physician. Treatment with mitotane was continued for a minimum of 2 years or until adrenocortical carcinoma recurrence or occurrence of unacceptable toxicity. The recommendation of the treatment duration was based on our practice and consensus among experts, due to the absence of specific studies.^6^

Assessments were scheduled at baseline and every 12 weeks for 2 years, both in the ADIUVO trial and the ADIUVO Observational study, and included history, physical examination, imaging, standard biochemical measurements, and hormone work-up. After 2 years, the follow-up intervals were decided by the local investigators and was done with visits every 3–4 months in both studies. Safety was assessed with the use of the National Cancer Institute Common Terminology Criteria for Adverse Events (version 3.0).^19^ Quality of life was assessed in the ADIUVO trial by using the European Organisation for Research and Treatment of Cancer (EORTC) quality-of-life core questionnaire (QLQ-C30, version 3.0;^20^ appendix p 27). Monitoring of study procedures and safety was organised on a country-specific basis by the respective coordinating centres.

### Outcomes

The primary endpoint was recurrence-free survival, defined as the time from randomisation to the first radiological evidence of recurrence or death from any cause (whichever occurred first); patients without recurrence were censored at the date of the last follow-up visit. Recurrence was defined as the appearance of a new lesion confirmed by imaging. Disease recurrence was evaluated with the use of contrast-enhanced CT or MRI at baseline and every 12 weeks from the date of randomisation. Secondary endpoints were overall survival, effects of plasma mitotane concentration on patient outcome, efficacy of mitotane in predefined subgroups of patients, measures of quality of life, and toxicities. Overall survival was measured from the date of randomisation to the date of death from any cause, and the data were censored at the date of the last follow-up visit. Details on how plasma mitotane concentrations were assessed are given in the appendix (p 23).

Both recurrence-free survival and overall survival in ADIUVO Observational were measured from the date of registration in the study.

Safety measures used in the study included physical examination and clinical laboratory tests (haematology, blood chemistries, and creatinine clearance).

### Statistical analysis

The sample size of the study was estimated to achieve sufficient power in the assessment of the effect of mitotane on recurrence-free survival (the primary endpoint). Based on retrospective data available at the time the study was designed,^8^, ^15^ in patients with low-intermediate risk of recurrence, a recurrence-free survival rate after 2 years was estimated to be approximately 60% with postoperative surveillance alone. The sample size was calculated to have 80% power to detect an increase in the proportion of patients free from recurrence at 2 years from 60% to 75% in the mitotane group, which was considered as the minimal clinically worthwhile improvement given the toxicity of mitotane treatment. This increase corresponds to a hazard ratio of 0·56. Considering a type I error level of 5% and using a two-sided log-rank test for analysis, it was calculated that 97 events in the primary endpoint should have been observed and approximately 184 patients should have been randomised, considering 4 years of accrual and 2 years of follow-up. Assuming a lost-to-follow-up rate of 10%, a total of 200 patients (100 per treatment group) were required. Two interim analyses had been planned at 20% and 60% of the information rate that, assuming a constant accrual rate, correspond to 19 expected events to occur in approximately 2 years, and 58 expected events to occur in approximately 4 years, respectively. Critical p values for stopping were set at p=0·00001 for the first interim analysis, p=0·01202 for the second analysis, and p=0·0464 for the final analysis, according to O’Brien-Fleming sequential design with maximum three stages, setting the family-wise α error at 0·05, assuming the survival times are exponential with uniform patient enrolment rate over time. No futility rules were set. The ADDPLAN software was used for the sample size calculation.

The assumed study duration for patients without recurrence was 2 years. In December, 2018, 10 years after the start of the randomised trial, only 91 patients had been enrolled in the randomised ADIUVO trial (recruitment per year is in the appendix p 15), and the Steering Committee decided to stop patient enrolment. This decision was not based on any stopping rules mentioned in the study protocol, but on the consideration that the recruitment was too slow to achieve the required number of patients in an acceptable time frame. Furthermore, funding was depleted. Of note, the Steering Committee was masked to study results at that time, since data collection was still ongoing and no interim analyses had been conducted.

Primary and secondary endpoints were analysed on the intention-to-treat population that included all randomly assigned participants. The per-protocol and safety populations included all participants who adhered to the assigned regimen, which was defined by taking at least one tablet of mitotane in the mitotane group and no mitotane at all in the surveillance group. Recurrence-free survival and overall survival in the two groups were estimated with the Kaplan-Meier method and compared using the log-rank test. The Cox's proportional hazard model was used to estimate the hazard ratios (HRs) with 95% CIs. Exploratory post-hoc intention-to-treat comparisons on the risk of recurrence at 5 years were made between treatment assignment and subgroups of interest defined by demographic characteristics (age and sex) and clinical characteristics (tumour size, stage, preoperative hormone secretion, Ki67 index, and surgical approach) at baseline, using a set of Cox's proportional hazard models that included an interaction term between the treatment assignment and the subgroup variable. Time to recurrence, defined as the time from randomisation to the time of recurrence, was also assessed. Data are restricted mean survival time and 95% CI, since the median survival was not reached.

The proportionality of hazards was checked using graphical diagnostics based on the scaled Schoenfeld residuals. Continuous variables are presented as medians and IQRs and were compared between groups using Mann-Whitney *U* test. Categorical variables are summarised as counts and percentages, and the χ^2^ test or Fisher test, as appropriate, was used for comparison. The distribution and median of follow-up duration were estimated using the reverse Kaplan-Meier method. The Wilcoxon-Mann-Whitney test was used to compare data on quality of life. The safety population, which included all the patients who had undergone randomisation and had received at least one dose of trial treatment, was used for all safety analyses.

In the ADIUVO Observational study, recurrence-free survival and overall survival in the mitotane and surveillance groups were compared using a Cox model, after adjusting for baseline covariate unbalances with an inverse probability weighting (IPW) approach based on propensity score (appendix pp 31–32).

All group comparisons in both studies were made with the use of a two-sided test at an α level of 5%. An independent data and safety monitoring board supervised the collection of efficacy and safety data. Statistical analyses were done with R (version 4.0.2) and SAS (version 9.4).

The ADIUVO trial is registered with ClinicalTrials.gov, NCT00777244.

### Role of the funding source

The funders of the study had no role in study design, data collection, data analysis, data interpretation, or writing of the report.

## Results

**Figure 1:**
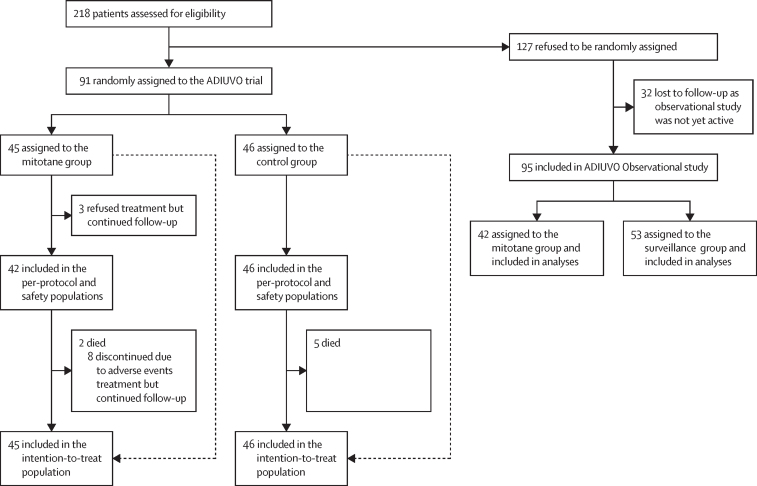
Trial profile

From Oct 23, 2008, to Dec 27, 2018, 45 patients were randomly assigned to receive mitotane treatment and 46 to surveillance alone (figure 1). Demographic and clinical characteristics of the patients are shown in table 1. 42 (93%) of 45 patients in the mitotane group and 38 (83%) of 46 patients in the surveillance group had follow-up visits every 3 months in the first 2 years. Mitotane was taken by 42 patients in the mitotane group, and median treatment duration was 21 months (IQR 6–24). The median daily dose of mitotane was 4 g (IQR 3–5) at 3 months, 3 g (2–4) at 6 months, and 2 g (1–3) both at 9 and 12 months. Plasma mitotane concentrations of 14 mg/L or higher were observed in 25 (60%) of 42 patients, with 13 (31%) of them reaching the target concentrations at 3 months. Three patients refused mitotane after randomisation but continued follow-up in the study. No patients in the surveillance alone group received mitotane during the study.

**Table 1:** Baseline characteristics in the intention-to-treat population

		**Mitotane group (n=45)**	**Surveillance group (n=46)**
Sex			
	Male	12 (27%)	15 (33%)
	Female	33 (73%)	31 (67%)
Age, years		51·0 (37·7–61·0)	50·5 (41·0–60·1)
Tumour ENS@T stage			
	I	9 (20%)	12 (26%)
	II	30 (67%)	29 (63%)
	III	6 (13%)	5 (11%)
Tumour size, mm		80 (49–127)	80 (50–122)
Ki67 index, %		5% (5–10)	5% (3–8)
Weiss		5 (4–6)	5 (4–6)
Preoperative hormone secretion*			
	Yes	20 (44%)	16/45 (36%)
	No	25 (56%)	29/45 (64%)
Preoperative overt cortisol excess*			
	Yes	7 (16%)	5/45 (11%)
	No	38 (84%)	40/45 (89%)
Surgery			
	Open	20 (44%)	25/45 (56%)
	Laparoscopy	25 (56%)	20/45 (44%)

Data are n (%), median (IQR), or n/N (%). ENS@T=European Network for the Study of Adrenal Tumors.

* Hormone secretion confirmed by laboratory data.

Between Jan 4, 2011, and Dec 16, 2018, 95 patients were included in the ADIUVO Observational study, of whom 42 received mitotane and 53 underwent surveillance alone (appendix p 30). Duration of mitotane treatment was 18 months (IQR 10–34), and target mitotane concentrations were observed in 26 (62%) patients. The databases of both the interventional trial and the observational study were closed for analysis on Dec 31, 2019.

**Figure 2:**
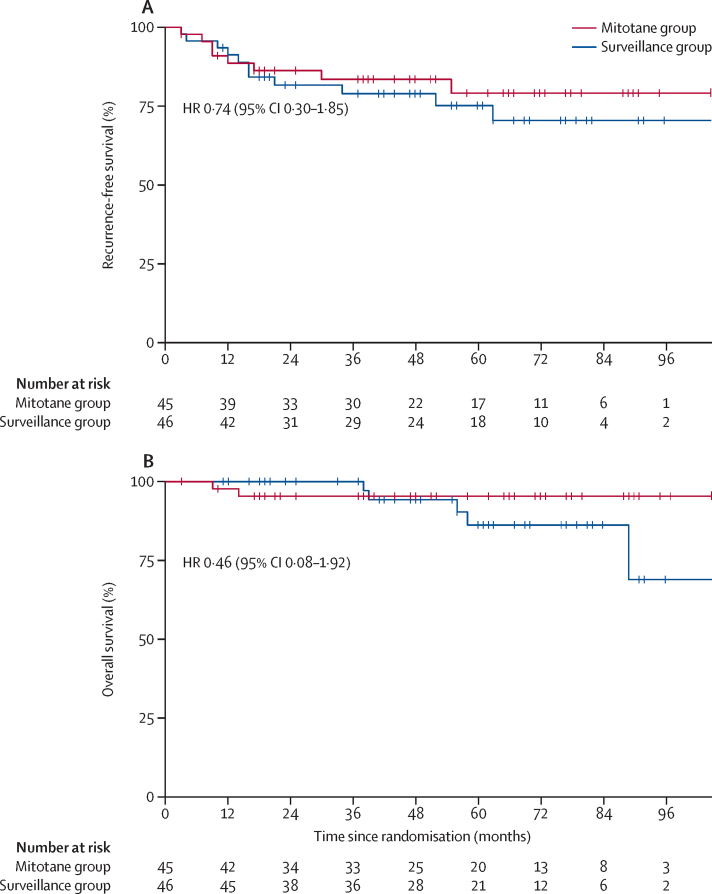
Survival in the intention-to-treat population Kaplan-Meier estimates of recurrence-free-survival (A) and overall survival (B).

**Figure 3:**
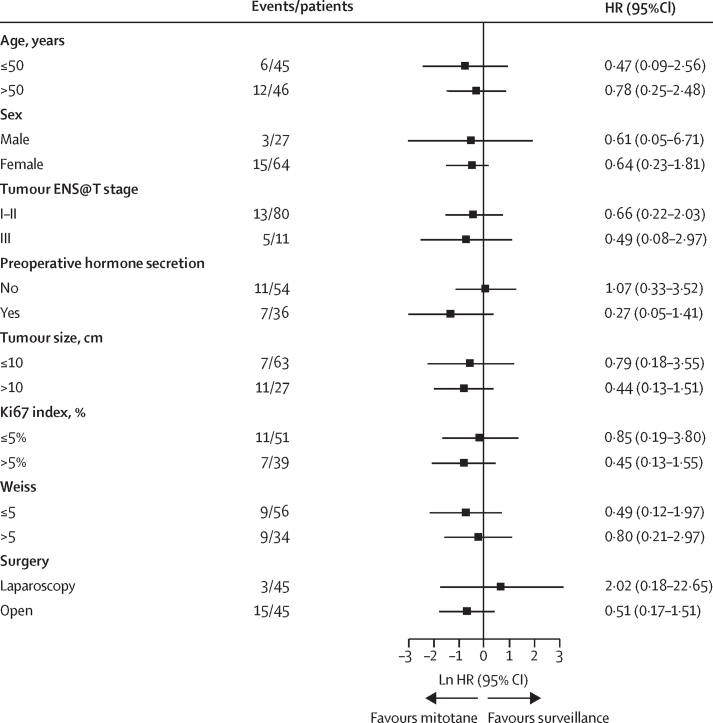
Subgroup analyses of the risk of recurrence at 5 years Error bars indicate the 95% CI for ln HR. ENS@T=European Network for the Study of Adrenal Tumors. HR=hazard ratio.

Among the 91 patients in the ADIUVO trial, disease recurrence occurred in 18 (20%) patients: seven (16%) of 45 in the mitotane group and 11 (24%) of 46 in the surveillance group. One death for pulmonary embolism in a patient free of disease was counted as an additional event in the mitotane group (table 2). Details on disease recurrence are given in the appendix (pp 19–20). Baseline characteristics did not differ between patients who had disease recurrence within 2 years or later (appendix pp 19–20). Median recurrence-free survival was not reached (95% CI not reached–not reached), and the median follow-up time for recurrence-free survival was 48 months (IQR 19–70; figure 2A). Estimated 5-year recurrence-free survival was 79% (95% CI 67–94) in the mitotane group and 75% (63–90) in the surveillance group (HR 0·74 [95% CI 0·30–1·85]). Similar results were obtained in the per-protocol analysis (appendix pp 16–18, 21). HRs for disease recurrence according to prespecified baseline factors did not show any relevant heterogeneity in the effect of mitotane in specific subgroups stratified by patient age and sex, tumour size and stage, preoperative hormone secretion, hormone production, Ki67 index, Weiss score, and surgical approach (figure 3). Moreover, we did not observe any significant difference in recurrence-free survival between patients in the mitotane group who did or did not reach the target plasma mitotane concentrations of 14–20 mg/L, and between patients who did or did not reach the target concentrations within 3 months (appendix pp 25–26). We also did not find any significant difference in the time in target range of plasma mitotane concentrations (appendix p 24).

**Table 2:** Efficacy in the intention-to-treat population

		**Mitotane group (n=45)**	**Surveillance group (n=46)**	**HR (95% CI)**
5-year recurrence-free survival		..	..	0·74 (0·30–1·85)
	Recurrences	7 (16%)	11 (24%)	..
	Local recurrences	3 (7%)	3 (7%)	..
	Distant or multiple recurrences	4 (9%)	8 (17%)	..
	Non-cancer-related death	1 (2%)	0	..
5-year overall survival		..	..	0·46 (0·08–1·92)
	Deaths	2 (4%)	5 (11%)	..
	Deaths from cancer	1 (2%)	4 (9%)	..
	Non-cancer-related death	1 (2%)	1 (2%)	..
Time to recurrence, months*		87·6 (76·8–98·5)	82·1 (70·4–93·8)	..
Quality of life at baseline†				
	Functional scale	69·1 (21·8)	81·7 (16·4)	..
	Symptoms scale	31·2 (21·9)	14·4 (13·1)	..
	Global health scale	62·2 (23·5)	70·8 (19·9)	..
Quality of life at follow-up‡				
	Functional scale	58·8 (19·1)	77·9 (19·1)	..
	Symptoms scale	41·2 (20·2)	19·3 (16·6)	..
	Global health scale	45·0 (25·7)	72·2 (19·3)	..

Data are n (%) and mean (SD) unless otherwise stated.

* Data are restricted mean survival time and 95% CI, since the median survival was not reached. Difference between groups is 5·52 months (95% CI −10·41 to 21·45).

† Assessed in 20 patients in the mitotane group and 18 patients in the surveillance group with the QLQ-C30 questionnaire.

‡ Assessed in 18 patients in the mitotane group and 15 patients in the surveillance group with the QLQ-C30 questionnaire.

In the ADIUVO Observational study, disease recurrence occurred in 17 (18%) of 95 patients, with eight (19%) in the mitotane group and nine (17%) in the surveillance alone group. Median recurrence-free survival was not reached (95% CI not reached–not reached), and the median follow-up time for recurrence-free survival was 34 months (IQR 16–67; appendix p 33). The IPW-adjusted rate of 5-year recurrence-free survival was 74% (95% CI 58–94) in the mitotane group and 72% (59–88) in the surveillance group (appendix p 33). 12 (71%) of 17 recurrences occurred within 2 years from surgery.

Seven (8%) of 91 patients died in the ADIUVO trial, with five deaths caused by progressive disease (one in the mitotane group and four in the surveillance group). Median overall survival was not reached (95% CI not-reached–not reached), and the median follow-up time for overall survival was 55 months (IQR 29–73). The rate of 5-year overall survival was 95% (95% CI 89–100) in the mitotane group and 86% (74–100) in the surveillance group (HR 0·46 [95% CI 0·08–1·92]; figure 2B).

In the ADIUVO Observational study, death was documented in three patients in the mitotane group and three patients in the surveillance group. The IPW-adjusted 5-year overall survival rate was 86% (95% CI 72–100) in the mitotane group and 90% (79–100) in the surveillance group (appendix p 33).

The QLQ-C30 questionnaire was completed by 20 (44%) of 45 patients in the mitotane group and 18 (39%) of 46 patients in the surveillance group at baseline, and by 18 (40%) patients in the mitotane group and 15 (33%) patients in the surveillance group at the 6-month evaluation. The scores of the functional scale, symptoms scale, global health status scale of the QLQ-C30 questionnaire were similar at baseline between patients receiving mitotane or active surveillance. Changes from baseline were apparent only in patients receiving mitotane, who experienced a decrease in the scores of the functional scale and global health scale with an increase in the score of the symptoms scale (table 2; appendix p 28).

There were no treatment-related deaths. All patients receiving mitotane reported adverse events that emerged during treatment or worsened as compared with the pre-treatment state. Table 3 lists all the adverse events documented that were mostly mild or moderate, and we did not record any grade 4 adverse events. In particular, no severe event related to adrenal insufficiency (adrenal crisis) or Cushing signs or symptoms was observed. However, eight (19%) of the 42 patients who received mitotane permanently discontinued mitotane after a median period of treatment of 6 months (IQR 4–7) for treatment-related toxicity. The causes of treatment discontinuation were liver (three), gastrointestinal (two), neurological (two), and haematological (one) toxicities. No unexpected serious adverse reaction was recorded. Of the 42 patients who received mitotane, six were treated until the time of death or last follow-up, and 24 of the remaining patients were able to discontinue glucocorticoid replacement after 6 months (three), 12 months (nine), or more than 12 months (12). 12 patients were still on glucocorticoid replacement as of Dec 31, 2019.

**Table 3:** Adverse events in the 42 patients who received mitotane

		**Grade 1**	**Grade 2**	**Grade 3**	**Grade 4**
**Anticipated adverse event**					
Constitutional symptoms					
	Asthenia or fatigue	11	13	3	0
Gastrointestinal symptom					
	Anorexia	13	5	1	0
	Diarrhoea	10	11	2	0
	Nausea or vomiting	13	12	1	0
Sexual symptoms					
	Gynaecomastia	2	2	1	0
	Erectile dysfunction	1	0	0	0
	Amenorrhoea	2	0	0	0
	Hot flushes	3	0	0	0
Metabolic symptoms					
	Elevated alanine or aspartate aminotransferase	10	3	3	0
	Elevated γ-glutamyl transferase	8	15	12	0
	Hypercholesterolaemia	12	10	1	0
	Hypertriglyceridaemia	11	4	1	0
Haematological symptoms					
	Anaemia	5	1	2	0
	Neutropenia	3	0	0	0
Other					
Cutaneous rush		9	0	0	0
	Lower limb oedema	5	0	0	0
**Adverse event of special interest**					
Neurological symptoms					
	Confusion	6	3	0	0
	Dizziness or vertigo	15	6	0	0
	Memory impairment	6	6	0	0
	Mood alteration	3	7	0	0
	Somnolence	9	2	0	0
	Tremor or paresthesia	7	0	2	0
Endocrine symptoms					
	Adrenal insufficiency	6	5	0	0

Adverse events were graded according to the National Cancer Institute's Common Terminology Criteria for Adverse Events (version 3.0). Multiple side-effects were reported by individual patients. Eight patients discontinued prematurely treatment because of grade 3 liver toxicity (three), grade 3 neurological toxicity (two), grade 3 gastrointestinal toxicity (two) and grade 3 haematological toxicity (one). Neurological symptom and adrenal insufficiency were considered as adverse events of special interest.

## Discussion

To our knowledge, ADIUVO is the first randomised trial to investigate an adjuvant treatment protocol in patients with completely resected adrenocortical carcinoma and, therefore, provides the best level of evidence currently available. Before the ADIUVO trial, limited evidence was available on adjuvant mitotane treatment, and the indications of available guidelines^1^, ^9^ on adjuvant mitotane treatment were based on a meta-analysis of six retrospective studies only including just 322 treated and 718 untreated patients.^2^, ^21^, ^22^, ^23^, ^24^, ^25^ In the ADIUVO trial, the observed reduction in the risk of adrenocortical carcinoma recurrence following complete resection of tumours with a Ki67 proliferation index of 10% or less in patients who received adjuvant mitotane did not reach significance (HR 0·74 [95% CI 0·30–1·85]). However, this finding should be interpreted considering that the power of the study was reduced by the following reasons: first, the prespecified sample size of the randomised trial was not reached and, second, the observed rate of recurrence was much lower than expected. Of note, the outcome results of the interventional ADIUVO trial were confirmed in the independent, prospective ADIUVO Observational study that included patients eligible for the trial but not randomly assigned. Many patients refused randomisation in the interventional trial; however, it is known that acceptance is lower for trials with an active treatment group compared with no treatment,^26^ and indeed the ADIUVO Observational study was organised to capture outcome data of patients who refused randomisation.

Despite the participation of many centres from different countries, the trial did not include the planned number of patients, which is not uncommon in investigator-sponsored trials in rare diseases. Furthermore, it is unlikely that a larger cohort will be recruited in the near future, because almost all international reference centres for this rare disease participated in the trial.

The prognosis of the ADIUVO cohort comprising patients with presumed low risk of recurrence was much better than previously anticipated, and this is a key finding of the study that has an impact on clinical practice. In previous studies including unselected cohorts of patients with adrenocortical carcinoma, 5-year recurrence-free survival was consistently less than 40%,^8^, ^21^, ^22^, ^23^, ^24^, ^25^ and, even in studies on patients with presumably moderate risk of recurrence, at least 50% of patients had disease recurrence.^2^, ^5^, ^15^, ^27^ The fact that the outcome of patients on surveillance alone was far better than anticipated, with an observed 5-year recurrence-free survival of 75% compared with a value of 60% used to design the study, strongly contributed to affect the power of the study to show a significant effect of treatment, if present. It has to be acknowledged that we had limited information on the natural history of patients with adrenocortical carcinoma at the time of designing the trial, with no available data from prospective studies.

Because of its low power, the ADIUVO trial cannot rule out an effect of adjuvant mitotane (appendix p 22); however, the observed good prognosis of patients with low-grade, localised adrenocortical carcinoma even with surveillance alone is a strong argument by itself against adjuvant treatment with mitotane that would convey only a potentially small absolute benefit in this patient population. An exceedingly large number of patients (about 1000 according to our calculations) would have been required to show a significant benefit of treatment, and such numbers are beyond the capability of recruitment of any studies in this disease. Although the advantage of mitotane is similar to that obtained with aromatase inhibitors over tamoxifen in adjuvant therapy of breast cancer,^28^ one must consider that the toxicity of mitotane is considerably higher than that of aromatase inhibitors.^1^, ^6^ Moreover, the challenges of adjuvant mitotane include a cumbersome regimen that entails a high number of tablets per day, a narrow therapeutic index, the need of a complex supportive therapy including high replacement doses of hydrocortisone, and a long treatment duration.^6^ Our results should be interpreted in the context of all the previous clinical considerations; therefore, we cannot recommend the use of adjuvant mitotane as standard of care in adult patients after complete resection of a low-grade, localised adrenocortical carcinoma. We did not find any significant influence of patient age and sex, tumour size and stage, hormone production, Ki67 index, Weiss score, and surgical approach on the effect of treatment.

In the future, analysis of molecular markers might further refine individual prognosis and indication to treatment.^6^ However, while some markers have been found to harbour predictive or prognostic value in retrospective studies (eg, *BUB1* and *PINK1* mRNA,^29^, ^30^ hypermethylation of *PAX5*,^31^, ^32^ *G0S2*,^33^ and *RRM1*^34^), it will be important to test their value prospectively in patients with localised disease who are allocated to adjuvant mitotane or surveillance alone after complete tumour resection.

The ADIUVO trial confirms in a prospective setting that adjuvant mitotane therapy is demanding even at reference centres, with 19% of patients discontinuing treatment definitively for toxicity before completing the scheduled 2-year period. Although this figure cannot be dismissed in an adjuvant setting, the ADIUVO trial shows that most patients can tolerate mitotane when they are managed at expert centres. The pattern of observed toxicity was expected, and most adverse events occurred quite early in the course of treatment independently on mitotane concentrations, thus outlining the role of individual sensitivity.^6^ Even if we did not observe severe toxicity, the concomitance of multiple adverse events and their duration led to discontinuation of treatment in some patients.

A low-dose mitotane regimen was used in most centres and the presumed therapeutic mitotane concentrations of 14–20 mg/L^17^, ^18^ were attained in about 60% of patients, a figure similar to that reported in a retrospective study in adjuvant setting in which, however, many patients have been treated with a high-dose regimen.^18^ The optimal dosing of mitotane is controversial:^6^ a high-dose regimen might lead to the target doses more rapidly, but it has the drawback of being potentially more toxic, thus affecting patient tolerability and the achievement of target concentrations. The ADIUVO trial shows that, when treating patients at perceived limited risk of recurrence, a low-dose regimen is favoured for the sake of safety. However, the difference between diverse dosing regimens is not of major relevance,^35^ concerning only the first weeks of treatment; thereafter, mitotane dose is adjusted on patient tolerability and plasma mitotane concentrations.

In the ADIUVO trial, survival of patients who did attain the target mitotane concentrations was not significantly different than that of patients who did not, either when considering the peak value of mitotane concentrations or the time in range, a method recently proposed to take into account the fluctuations of concentrations over time.^36^ We also did not observe any significant difference between patients who did or did not attain the target concentrations within 3 months; however, the power of these analyses is low. The optimal duration of adjuvant mitotane therapy remains to be defined; however, a retrospective study has shown that prolonging adjuvant mitotane treatment for more than 2 years does not provide a survival advantage.^37^

To our knowledge, for the first time, the ADIUVO trial provides measures of the quality of life collected prospectively in patients receiving adjuvant mitotane treatment. Although the low compliance in answering the questionnaires limits the interpretation of data, our findings suggest that patients receiving mitotane experienced a worse quality of life than patients on active surveillance.

Furthermore, the ADIUVO trial confirms in a prospective setting the concept that adrenocortical carcinoma is not a uniform disease, substantiating genomic studies that have identified specific molecular signatures associated with differences in prognosis in localised adrenocortical carcinoma.^30^, ^31^, ^32^, ^33^, ^38^, ^39^, ^40^ In the ADIUVO trial, the frequency of cortisol-secreting adrenocortical carcinoma was lower than usually reported in unselected patient series,^2^, ^8^, ^21^, ^22^, ^23^, ^24^, ^25^, ^27^ and this finding is in agreement with studies showing that the molecular profile of steroidogenetic differentiation is less represented among adrenocortical carcinoma with good prognosis.^38^, ^39^

The ADIUVO findings further validate prospectively the value of the proliferation marker Ki67 for prognostication^2^, and the cutoff value of 10% as useful to identify a low to moderate risk category of patients. Given that Ki67 analysis is widely available,^1^ this information is readily transferrable to clinical practice.

We acknowledge that the ADIUVO trial has limitations, including the fact that the study was small, terminated early, had an open-label design, and is possibly prone to the effect of time in estimating the treatment response. However, we considered a blind, placebo-controlled study to be unfeasible because high-dose preventive steroid replacement is needed with mitotane treatment.^6^

In summary, the ADIUVO findings provide insufficient evidence of mitotane efficacy and suggest that active surveillance is the most adequate concept for patients with low-grade, localised adrenocortical carcinoma. ADIUVO indicates that a complex and toxic treatment can be spared and management can be simplified for this cohort with better quality of life and lower costs. However, the results of the ADIUVO trial should not be generalised to patients with adrenocortical carcinoma at standard or high risk of recurrence for whom adjuvant mitotane treatment should still be considered standard of care.

## Data sharing

We will consider sharing de-identified, individual participant-level data that underlie the results reported in this Article on receipt of a request detailing the study hypothesis and statistical analysis plan. All requests should be sent to the corresponding author. The corresponding author and lead investigators of this study will discuss all requests and make decisions about whether data sharing is appropriate based on the scientific rigour of the proposal. All applicants will be asked to sign a data access agreement.

## Declaration of interests

MT has received research grant from HRA Pharma and Novartis, and advisory board honoraria from HRA Pharma. AB has received advisory board honoraria from HRA Pharma. All other authors declare no competing interests.
