# Correction to *Lancet Diabetes Endocrinol* 2023; 11: 720–30

**DOI:** 10.1016/S2213-8587(23)00319-4

**Published:** 2023-12

*Terzolo M, Fassnacht M, Perotti P, et al. Adjuvant mitotane versus surveillance in low-grade, localised adrenocortical carcinoma (ADIUVO): an international, multicentre, open-label, randomised, phase 3 trial and observational study.* Lancet Diabetes Endocrinol *2023; **11:** 720–30*—The Summary and Acknowledgments of this Article should have included the following funder: AIRC Foundation for Cancer Research in Italy (Fondazione AIRC per la Ricerca sul Cancro). This correction has been made to the online version as of Nov 21, 2023.

