# Supplementary material for: Adjuvant mitotane versus surveillance in low-grade, localised adrenocortical carcinoma (ADIUVO): an international, multicentre, open-label, randomised, phase 3 trial and observational study
Source: Lancet Diabetes Endocrinol. 2023 Oct;11(10):720–30. doi: 10.1016/S2213-8587(23)00193-6 (PMC10522778; doi:10.1016/S2213-8587(23)00193-6)
Supplement: Supplementary appendix [file mmc1.pdf]

THE LANCET
Diabetes & Endocrinology

Supplementary appendix

This appendix formed part of the original submission and has been peer reviewed. We post it as supplied by the authors.

Supplement to: Terzolo M, Fassnacht M, Perotti P, et al. Adjuvant mitotane versus surveillance in low-grade, localised adrenocortical carcinoma (ADIUVO): an international, multicentre, open-label, randomised, phase 3 trial and observational study. *Lancet Diabetes Endocrinol* 2023; published online Aug 21. https://doi.org/10.1016/S2213-8587(23)00193-6.

SUPPLEMENTARY APPENDIX

Supplement to: M. Terzolo, M. Fassnacht, P. Perotti, et al. "Adjuvant mitotane in low-grade, localized adrenocortical carcinoma (ADIUVO): an international, multicentre, open-label, randomised, phase III study."

CONTENTS

Supplementary material -ADIUVO trial-

CONSORT checklist for Abstract page 3

CONSORT checklist page 4

CONSORT HARMS checklist page 8

DELTA2 sample size checklist page 12

Supplementary Table 1. Centre-specific patient contribution and ethnicity. page 14

Supplementary Figure 1. Recruitment per year in the ADIUVO trial. page 15

Supplementary Table 2. Baseline characteristics in the Per Protocol Population. page 16

Supplementary Table 3. Table of efficacy in the Per Protocol Population. page 18

Supplementary Table 4. Characteristics of patients with disease recurrence. page 19

Supplementary Table 5. Characteristics of patients with disease recurrence within 2 years or later. page 20

Supplementary Figure 2. Survival in the Per Protocol Population. page 21

Supplementary Table 6. Posterior power estimates in the ADIUVO trial. page 22

Effects of plasma mitotane concentrations on patient outcome page 23

Supplementary Table 7. Studies on the therapeutic impact of plasma mitotane concentrations. page 24

Supplementary Figure 3. Survival of patients according to plasma mitotane concentrations. page 25

Supplementary Figure 4. Survival of patients according to the time needed to reach plasma mitotane concentrations ≥14 mg/L. page 26

Measures of Quality of Life page 27

Supplementary Table 8. Quality of Life according to the EORTC QLQ-C30 questionnaire. page 28

Supplementary material -ADIUVO Observational study-

Supplementary Table 9. Centre-specific patient contribution in the ADIUVO Observational Study. page 29

Supplementary Figure 5. Flow diagram of the ADIUVO Observational study. page 30

Additional statistical analyses -ADIUVO Observational study- page 31

Supplementary Table 10. Baseline characteristics of patients in the ADIUVO Observational study. page 32

Supplementary Figure 6. Survival of patients in the ADIUVO Observational study. page 33

References to supplemental material page 34

Study Protocol page 35

Statistical Analysis Plan page 119

CONSORT checklist for Abstract.

Item	Description	Line
Title	Identification of the study as randomized	1-3
Authors*	Contact details for the corresponding author	n/a
Trial design	Description of the trial design (e.g. Parallel, cluster non –inferiority).	95, 96
Methods		
Participants	Eligibility criteria for participants and the settings where the data were collected	96-98
Interventions	Interventions intended for each group	101, 102
Objective	Specific objective or hypothesis	98, 99
Outcome	Clearly defined primary outcome for this report	98, 99
Randomization	How participants were allocated to interventions	101
Blinding (masking)	Whether or not participants, care givers, and these assessing the outcomes were blinded to group assignment	95
Results		
Number randomised	Number of participants randomized to each group	101, 102
Recruitment	Trial status	99
Numbers analysed	Number of participants analyzed in each group	101, 102
Outcome	For the primary outcome a result for each group and the estimated effect size and its precision	102, 103
Harms	Important adverse events or side-effects	105-107
Conclusion	General interpretation of the results	109-112
Trial Registration	Registration number and name of trial registry	95
Funding	Sources of funding	114

*For conference abstracts.

Reporting checklist for randomised trial
CONSORT checklist.

		Reporting Item	Page Number
**Title and Abstract**			
Title	1a	Identification as a randomized trial in the title	1
Abstract	1b	Structured summary of trial design, methods, results, and conclusions	4
**Introduction**			
Background and objectives	2a	Scientific background and explanation of rationale	8
Background and objectives	2b	Specific objectives or hypothesis	8
**Methods**			
Trial design	3a	Description of trial design (such as parallel, factorial) including allocation ratio.	10
Trial design	3b	Important changes to methods after trial commencement (such as eligibility criteria), with reasons	14[1]
Participants	4a	Eligibility criteria for participants	10
Participants	4b	Settings and locations where the data were collected	10
Interventions	5	The experimental and control interventions for each group with sufficient details to allow replication, including how and when they were actually administered	11, 12

Outcomes	6a	Completely defined prespecified primary and secondary outcome measures, including how and when they were assessed	12, 13
Outcomes	6b	Any changes to trial outcomes after the trial commenced, with reasons	n/a
Sample size	7a	How sample size was determined.	13
Sample size	7b	When applicable, explanation of any interim analyses and stopping guidelines	n/a
Randomization - Sequence generation	8a	Method used to generate the random allocation sequence.	10
Randomization - Sequence generation	8b	Type of randomization; details of any restriction (such as blocking and block size)	10
Randomization - Allocation concealment mechanism	9	Mechanism used to implement the random allocation sequence (such as sequentially numbered containers), describing any steps taken to conceal the sequence until interventions were assigned	10, 11
Randomization - Implementation	10	Who generated the allocation sequence, who enrolled participants, and who assigned participants to interventions	10
Blinding	11a	If done, who was blinded after assignment to interventions (for example, participants, care providers, those assessing outcomes) and how.	n/a[2]
Blinding	11b	If relevant, description of the similarity of interventions	n/a[2]
Statistical methods	12a	Statistical methods used to compare groups for primary and secondary outcomes	14, 15
Statistical methods	12c	Methods for additional analyses, such as subgroup analyses and adjusted analyses	14, 15

Results

Participant flow diagram (strongly recommended)	13a	For each group, the numbers of participants who were randomly assigned, received intended treatment, and were analysed for the primary outcome	16 and Fig.1
Participant flow	13b	For each group, losses and exclusions after randomization, together with reason	16 and Fig.1
Recruitment	14a	Dates defining the periods of recruitment and follow-up	16
Recruitment	14b	Why the trial ended or was stopped	14
Baseline data	15	A table showing baseline demographic and clinical characteristics for each group	Table 1
Numbers analysed	16	For each group, number of participants (denominator) included in each analysis and whether the analysis was by original assigned groups	16 and Fig. 1
Outcomes and estimation	17a	For each primary and secondary outcome, results for each group, and the estimated effect size and its precision (such as 95% confidence interval)	16, 17
Outcomes and estimation	17b	For binary outcomes, presentation of both absolute and relative effect sizes is recommended	16, 17
Ancillary analyses	18	Results of any other analyses performed, including subgroup analyses and adjusted analyses, distinguishing pre-specified from exploratory	16, 17
Harms	19	All important harms or unintended effects in each group (For specific guidance see CONSORT for harms)	17, 18

Discussion

Limitations	20	Trial limitations, addressing sources of potential bias, imprecision, and, if relevant, multiplicity of analyses	20-22
Generalisability	21	Generalisability (external validity, applicability) of the trial findings	20, 23
**Other information**			
Interpretation	22	Interpretation consistent with results, balancing benefits and harms, and considering other relevant evidence	20
Registration	23	Registration number and name of trial registry	4, 15
Protocol	24	Where the full trial protocol can be accessed, if available	appendix
Funding	25	Sources of funding and other support (such as supply of drugs), role of funders	4, 15, 24

[1]Due to limited number of patients recruited, an interim analysis was not performed. Due to increasingly disappointing recruitment rate and financial difficulties, the Steering Committee decided to stop enrolment in the study on December 2018. This decision was taken blinded to study results, since data collection was still ongoing and no interim analyses had been conducted.

[2]The trial was conducted unblinded. A blind placebo-controlled study was considered unfeasible since patients treated with mitotane are at risk of adrenal insufficiency and need preventive steroid coverage, which may have detrimental consequences in patients who are on surveillance only. Since the outcomes (recurrence and death) are not subjective, outcome adjudication was not blinded.

Reporting checklist for randomised trial.

CONSORT HARMS checklist.

		Reporting Item	Page Number
**Title and Abstract**			
Title	1a	-	
Abstract	1b	Structured summary of trial design, methods, results of outcomes of benefits and harms, and conclusions	4
**Introduction**			
Background and objectives	2a	-	
Background and objectives	2b	Specific objectives or hypothesis for outcomes of benefits and harms	8
**Methods**			
Trial design	3a	-	
Trial design	3b	-	
Participants	4a	-	
Participants	4b	-	
Interventions	5	-	
Outcomes	6a	Completely defined prespecified primary and secondary outcomes, for both benefits and harms, including how and when they were assessed	12, 13
Outcomes	6b	-	
	6c	Describe if and how non-prespecified outcomes of benefits and harms were identified, including any selection criteria, if applicable	n/a

Sample size	7a	-	
Sample size	7b	-	
Randomization - Sequence generation	8a	-	
Randomization - Sequence generation	8b	-	
Randomization - Allocation concealment mechanism	9	-	
Randomization - Implementation	10	-	
Blinding	11a	If done, who was blinded after assignment to interventions (for example, participants, care providers, those assessing outcomes of benefits and harms) and how	n/a[1]
Blinding	11b	-	
Statistical methods	12a	Statistical methods used to compare groups for primary and secondary outcomes of both benefits and harms	14, 15
Statistical methods	12b	-	
**Results**			
Participant flow diagram (strongly recommended)	13a	For each group, the numbers of participants who were randomly assigned, received intended treatment, and were analysed for the primary outcome of benefits and harms	16 and Fig.1
Participant flow	13b	-	
Recruitment	14a	Dates defining the periods of recruitment and follow-up for outcomes of benefits and harms	16

Recruitment	14b	-	
Baseline data	15	-	
Numbers analysed	16	For each group, number of participants (denominator) included in each analysis of outcomes of benefits and harms and whether the analysis was by original assigned groups and if any exclusions were made	16-18 and Fig.1
Outcomes and estimation	17a	For each primary and secondary outcome of benefits and harms, results for each group, and the estimated effect size and its precision (such as 95% confidence interval)	16 and Figure 3
Outcomes and estimation	17a2	For outcomes omitted from the trial report (benefits and harms), provide rationale for not reporting and indicate where the data on omitted outcomes can be accessed	n/a
Outcomes and estimation	17b	Presentation of both absolute and relative effect sizes is recommended, for outcomes of benefits and harms	16-18
Outcomes and estimation	17c	Report zero events if no harms were observed	Table 3
Ancillary analyses	18	Results of any other analyses performed for outcomes of benefits and harms, including subgroup analyses and adjusted analyses, distinguishing prespecified from exploratory	16, 17
Harms	19	-	
**Discussion**			
Limitations	20	Trial limitations, addressing sources of potential bias related to the approach to collecting or reporting data on harms, imprecision, and, if relevant, multiplicity or selection of analyses	20-22

Generalisability	21	-	
**Other information**			
Interpretation	22	-	
Registration	23	-	
Protocol	24	Whether the full trial protocol and other relevant documents can be assessed, including additional data on harms	appendix
Funding	25	-	

[1]The trial was conducted unblinded. A blind placebo-controlled study was considered unfeasible since patients treated with mitotane are at risk of adrenal insufficiency and need preventive steroid coverage, which may have detrimental consequences in patients who are on surveillance only. Since the outcomes (recurrence and death) are not subjective, outcome adjudication was not blinded.

Delta[2] recommended reporting items for the sample size calculation of a randomized controlled trial with a superiority question.

	Recommended reporting items	Page and line numbers where item is reported
**Core items**		
1	Primary outcome (and any other outcome on which the calculation is based)	Page 12, line 269
	If a primary outcome is not used as the basis for the sample size calculation, state why	NA
2	Statistical significance level and power	Page 13, line 289-293
3	Express the target difference according to outcome type	Page 13 line 290-292
	(a) Binary - state the target difference as an absolute or relative effect (or both), along with the intervention and control group proportions. If both an absolute and a relative difference are provided, clarify if either takes primacy in terms of the sample size calculation	NA
	(b) Continuous – state the target mean difference on the natural scale, common standard deviation, and standardized effect, size (mean difference divided by the standard deviation)	NA
	(c) Time-to-event – state the target difference as an absolute or relative difference (or both); provide the control group event proportion, planned length of follow-up, intervention and control group survival distributions, and accrual time (if assumptions regarding them are made). If both an absolute and relative difference are provided for a particular time point, clarify if either takes primacy in terms of the sample size calculation	Page 13 line 290-292
4	Allocation ratio	Page 10, line 222
	If an unequal ratio is used, the reason for this should be stated	NA
5	Sample size based on the assumptions as per above	Page 13, line 285

	(a) Reference the formula/sample size calculation approach, if standard binary, continuous, or survival outcome formulas are not used. For a time-to-event outcome, the number of events required should be stated	Page 13, line 294
	(b) If any adjustments (eg, allowance for loss to follow-up, multiple testing) that alter the required sample size are incorporated, they should also be specified, referenced, and justified along with the final sample size	Pag 13, line 296-299
	(c) For alternative designs, additional input should be stated and justified. For example, for a cluster randomized controlled trial (or an individually randomized controlled trial with clustering), state the average cluster size and intracluster correlation coefficient(s). Variability in cluster size should be considered and, if necessary, the coefficient of variation should be incorporated into the sample size calculation. Justification for the values chosen should be given	NA
	(d) Provide details of any assessment of the sensitivity of the sample size to the inputs used	NA

Additional items for grant application and trial protocol

6	Underlying basis used for specifying the target difference (an important or realistic difference)	Page 13, line 291
7	Explain the choice of target difference – specify and reference any formal method used or relevant previous research	Page 13, line 287

Additional items for trial results paper

8	Reference the trial protocol	Pag 15, line 342

Supplementary Table 1. Centre-specific patient contribution and ethnicity.

Centre	Country	All patients (n=91, 100%)	Ethnicity	
			Caucasian (n=88, 96·7%)	African (n=3, 3·3%)
Würzburg	Germany	18	18	0
Orbassano	Italy	17	17	0
Brescia	Italy	7	7	0
Paris	France	7	4	3
Villejuif	France	6	6	0
Munich	Germany	5	5	0
Zagreb	Croatia	5	5	0
Reims	France	4	4	0
Lyon	France	4	4	0
Milan	Italy	4	4	0
Montreal	Canada	4	4	0
Eindhoven	The Netherlands	2	2	0
Berlin	Germany	1	1	0
Birmingham	UK	1	1	0
Bordeaux	France	1	1	0
Ferrara	Italy	1	1	0
Grenoble	France	1	1	0
Rome	Italy	1	1	0
Toulouse	France	1	1	0
Turin	Italy	1	1	0

Supplementary Figure 1. Recruitment per year in the ADIUVO trial.

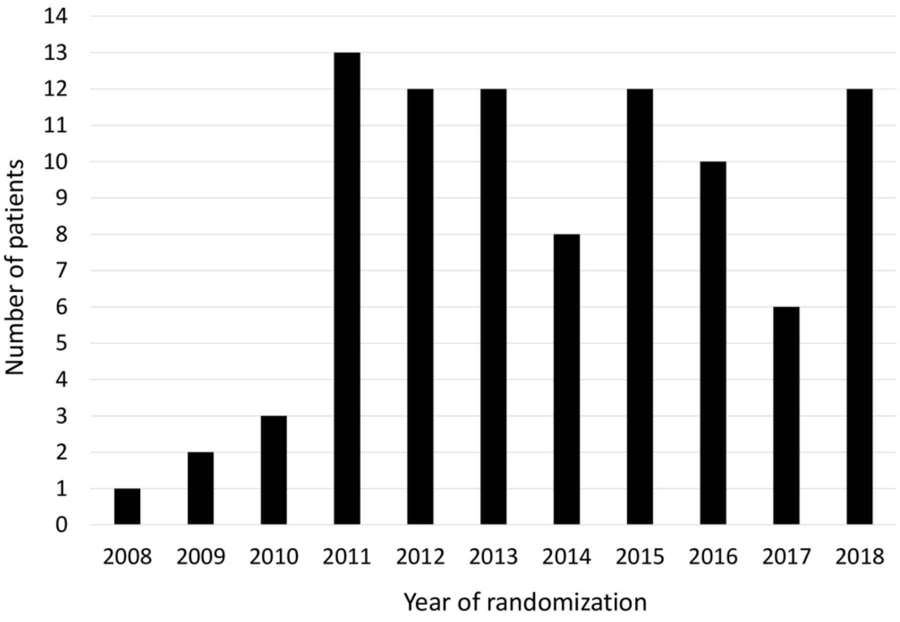

Supplementary Table 2. Baseline characteristics in the Per Protocol Population.

**Characteristic**	Valid cases	Surveillance (N=46)	Mitotane (N=42)
Sex — no. (%)	88		
Male		15 (32.6)	11 (26.2)
Female		31 (67.4)	31 (73.8)
Age — yrs	88		
Median		50.5	50.5
IQR		41.0—60.1	37.2—60.7
Tumor stage — no. (%)	88		
I		12 (26.1)	8 (19.0)
II		29 (63.0)	28 (66.7)
III		5 (10.9)	6 (14.3)
Tumor size — mm	88		
Median		80	100
IQR		50—122	80—150
Ki67 index —%	88		
Median		5.0	5.0
IQR		3.0—8.0	5.0—10
Weiss	88		

Median		5	5
IQR		4—6	4—6
Preoperative hormone secretion§ — no. (%)	87		
Yes		16 (35.6)	20 (47.6)
No		29 (64.4)	22 (52.4)
Preoperative overt cortisol excess# — no. (%)	87		
Yes		5 (11.1)	7 (16.7)
No		40 (88.9)	35 (83.3)
Surgery — no. (%)	87		
Open		25 (55.6)	19 (45.2)
Laparoscopy		20 (44.4)	23 (54.8)

IQR denotes interquartile range
§Hormone secretion has been confirmed by laboratory data
#Clinically apparent Cushing phenotype

Supplementary Table 3. Table of efficacy in the Per Protocol Population.

Characteristic	Surveillance (N=46)	Mitotane (N=42)	HR (95% CI)
Recurrence-free Survival§	11	8	0.78 (0.31 -1.95)
Local recurrences	3 (6.5)	3 (6.7)	
Distant/multiple recurrences	8 (17.4)	4 (8.9)	
Non-cancer-related death	0 (0.0)	1 (2.2)	
Overall Survival§	5	2	0.42 (0.08 – 2.18)
Death from cancer	4 (8.7)	1 (2.2)	
Non-cancer-related death	1 (2.2)	1 (2.2)	
Time to Recurrence (months)¶	82.1 (70.4-93.8)	86.8 (75.5-98.1)	
Quality of Life (baseline)#	*(18 valid cases)*	*(20 valid cases)*	
Functional scale*	82.2 (67.1-98.2)	71.1 (53.3-85.9)	
Symptoms scale*	11.5 (3.2-25.0)	41.0 (9.4-47.7)	
Global health scale*	75.0 (53.5-83.3)	58.3 (41.7-83.3)	
Quality of Life (follow-up)#	*(15 valid cases)*	*(18 valid cases)*	
Functional scale*	84.4 (63.3-91.1)	55.6 (45.5-70.7)	
Symptoms scale*	17.9 (3.8-27.8)	38.5 (28.2-55.1)	
Global health scale*	66.7 (66.7-83.3)	50.0 (33.3-58.3)	

§Data are expressed as number and (%)

¶Data are expressed as Restricted Mean Survival Time (RMST) and 95% CI, since the median survival was not achieved. Difference between arms is 4.69 (95%CI, -11.56-20.94)

# Wilcoxon-Mann-Whitney test was used to compare data on quality of life. No statistically significant differences were observed at baseline, while at follow-up differences in all items were statistically significant. A higher value in the Functional Scale and Global Health Scale corresponds to a better quality of life while a lower value corresponds in the Symptoms Scale corresponds to a better quality of life.

*Data are expressed as median and IQR (interquartile range)

Supplementary Table 4. Characteristics of patients with disease recurrence.

Characteristic	Valid cases	Distant/multiple (N=12)	Local (N=6)
Arm — no. (%)	18		
Mitotane		4 (33·3)	3 (50·0)
Surveillance		8 (66·7)	3 (50·0)
Sex — no. (%)	18		
Male		1 (8·3)	2 (33·3)
Female		11 (91·7)	4 (66·7)
Age — yrs	18		
Median		57·0	49·5
IQR		50·2—62·5	40·2—55·7
Tumour stage — no. (%)	18		
I		0 (0·0)	0 (0·0)
II		8 (66·7)	5 (83·3)
III		4 (33·3)	1 (16·7)
Tumour size — mm	18		
Median		135	110
IQR		90—175	80—150
Ki67 index —%	18		
Median		8·0	4·0
IQR		6·2—10·0	3·0—8·7
Weiss	18		
Median		6	5
IQR		5—7	5—6
Hormone excess	18		
Yes		2 (16·7)	1 (16·7)
No		10 (83·3)	5 (83·3)
Surgery — no. (%)	18		
Open		11 (91·7)	4 (66·7)
Laparoscopy		1 (8·3)	2 (33·3)
Treatment of recurrence	18		
Local therapies only§		5 (41·7)	3 (50·0)
Mitotane ± local therapies		4 (33·3)	3 (50·0)
Chemotherapy		3 (25·0)	0

§Local therapies include surgery, radiotherapy, radiofrequency, chemo-embolization.
IQR = interquartile range.

Supplementary Table 5. Characteristics of patients with disease recurrence within 2 years or later.

**Characteristic**	Valid cases	Early (≤ 2 years) (N=13)	Late (> 2 years) (N=5)
Arm — no. (%)	18		
Mitotane		5 (38·5)	2 (40·0)
Surveillance		8 (61·5)	3 (60·0)
Sex — no. (%)	18		
Male		2 (15·4)	1 (20·0)
Female		11 (84·6)	4 (80·0)
Age — yrs	18		
Median		57·0	47·0
IQR		53·0—64·0	41·0—52·0
Tumour stage — no. (%)	18		
I		0 (0·0)	0 (0·0)
II		10 (76·9)	3 (60·0)
III		3 (23·1)	2 (40·0)
Tumour size — mm	18		
Median		120	115
IQR		90—160	75—165
Ki67 index —%	18		
Median		8·0	3·0
IQR		5·0—10·0	3·0—7·0
Weiss	18		
Median		7	5
IQR		5—7	5—5
Hormone excess	18		
Yes		6 (46·2)	1 (20·0)
No		7 (53·8)	4 (80·0)
Surgery — no. (%)	18		
Open		11 (84·6)	4 (80·0)
Laparoscopy		2 (15·4)	1 (20·0)
Type of recurrence — no. (%)	18		
Distant/multiple		10 (76·9)	3 (60·0)
Local		3 (23·1)	2 (40·0)

IQR = interquartile range

Supplementary Figure 2. Survival in the Per Protocol Population. Kaplan-Meier estimates of recurrence-free-survival (Upper Panel) and overall survival (Lower Panel). Red line indicates the mitotane group and blue line the surveillance group.

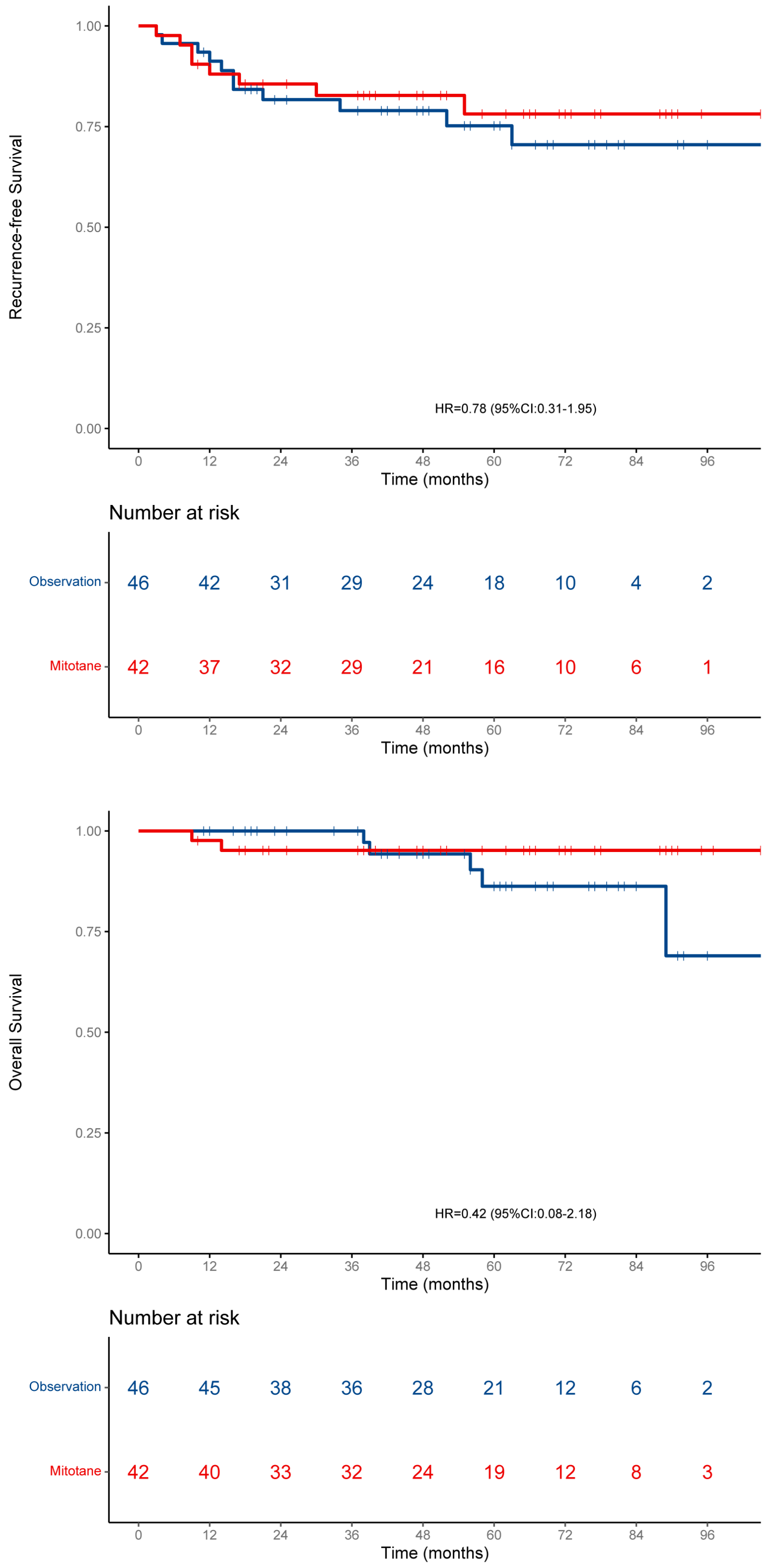

Additional statistical analyses - ADIUVO trial -

Supplementary Table 6. Posterior power estimates in the ADIUVO trial.

Scenario	Target Hazard Ratio	N. Events	Power
Original study design	0.56	97	80%
Power vs target Hazard Ratio	0.56	19	24%
Power vs most plausible Hazard Ratio*	0.42	19	47%
True Hazard Ratio for having power of 80%	0.27	19	80%
Minimal Statistically Significant Hazard Ratio (p=0.05)	0.38	19	-

*Estimated as the (geometric) mean of the 2 Hazard Ratios obtained from a retrospective study in patients with ACC comparing a group of 47 patients on adjuvant mitotane with 2 different control groups of 55 and 75 patients, respectively, on surveillance only [1].

Effects of plasma mitotane concentrations on patient outcome

The mitotane dose has been adjusted to patient tolerability and physician preference aiming to achieve a plasma level of ≥14 mg/L (or the maximum tolerated dose), since that level has been previously associated with the therapeutic effect of the drug (Supplementary Table 7). Analysis of circulating mitotane levels was done by a central laboratory (Lysosafe® service, HRA Pharma, France).

Of the 45 patients assigned to adjuvant treatment with mitotane, 42 received the drug for at least one month and 25 of them (59.5%) reached plasma mitotane concentrations ≥14 mg/L. Thirteen of these patients (28.8%) reached the target concentrations within 3 months. The daily dose of mitotane was 4 (3-5) grams at the 3-month follow-up, 2.5 (1.5-3.5) grams at 6 months, 2 (0.5-3) grams at 9 months and 2 (1-4) grams 12 months. Median mitotane levels were 9.3 mg/L (5.8-13.7 mg/L) at 3 months, 13.2 mg/L (6.3-17.8 mg/L) at 6 months, 12.7 mg/L (6.0-17.8 mg/L) at 9 months and 11.8 mg/dL (5.7-15.4 mg/L) at 12 months.

No difference in the risk of recurrence between the 25 patients who did reach the therapeutical level and the 17 who did not was found (HR 0.59; 95%CI, 0.15-2.36) (Supplementary Figure 3A).

No difference in the OS between the 25 patients who did reach the therapeutical level and the 17 who did not was found (HR 0.61; 95%CI, 0.04-9.71) (Supplementary Figure 3B).

Supplementary Table 7. Studies on the therapeutic impact of plasma mitotane concentrations.

Study	Setting	Patient N.	Method to assess exposure	Main findings
Haak et al, 1994 [2]	Adjuvant<br>Advanced	11<br>51	Maintenance levels*	Levels ≥ 14 mg/L are associated with objective tumour response in advanced ACC. No effect in adjuvant setting.
Baudin et al, 2001 [3]	Adjuvant<br>Advanced	11<br>13	Peak level	Levels ≥ 14 mg/L are associated with objective tumour response for advanced ACC. No effect in adjuvant setting.
Hermsen et al, 2011 [4]	Advanced	91	Peak level§	Levels ≥ 14 mg/L are associated with longer OS.
Terzolo et al, 2013 [5]	Adjuvant	122	Percentage of measurements ≥ 14 mg/L	≥ 75% of measurements ≥ 14 mg/L are associated with longer RFS.
Megerle et al, 2018 [6]	Advanced	127	Peak level	Levels ≥ 14 mg/L are associated with longer OS.
Puglisi et al, 2019 [7]	Adjuvant	110	TTR	TTR is associated with longer RFS.
Puglisi et al, 2020 [8]	Advanced	80	TTR	TTR is associated with longer RFS.

*No details on definition of maintenance levels.
§Level closest to the date of best response or progression.
ACC = adrenocortical carcinoma; OS = overall survival; RFS = recurrence free survival; TTR = time in target range (levels ≥ 14 mg/L).

For the previous analysis, we have stratified our patients by their peak plasma mitotane concentration, with level ≥14 mg/L defining the category in target range. This is the method most frequently used in previous studies, as summarized in the supplementary table 5. This method has inherent limitations since it fails to capture the variability in plasma mitotane levels that may produce remarkable fluctuations in individual subjects. To overcome this limit, we have recently proposed to calculate the time in target range (TTR) of plasma mitotane concentrations, adopting a method analogous to that used for the anticoagulant drug warfarin [7]. The TTR did not differ in a statistically significant manner between patients who have disease recurrence or not. The median TTR in patients with ACC recurrence was 0 (IQR 0-6) vs. 4 (IQR 0-15.8) in patients without recurrence; however, the power of this analysis is limited (n=7 patients with recurrence compared to n=35 patients without).

Supplementary Figure 3. Survival of patients according to plasma mitotane concentrations. Kaplan-Meier plot for the risk of recurrence (panel A) and death (panel B) over 5 years according to the therapeutical levels. The red line indicates the patients who did reach the therapeutic levels and the blue line indicates the patients who did not. The dashed lines represent the respective 95% Confidence Intervals.

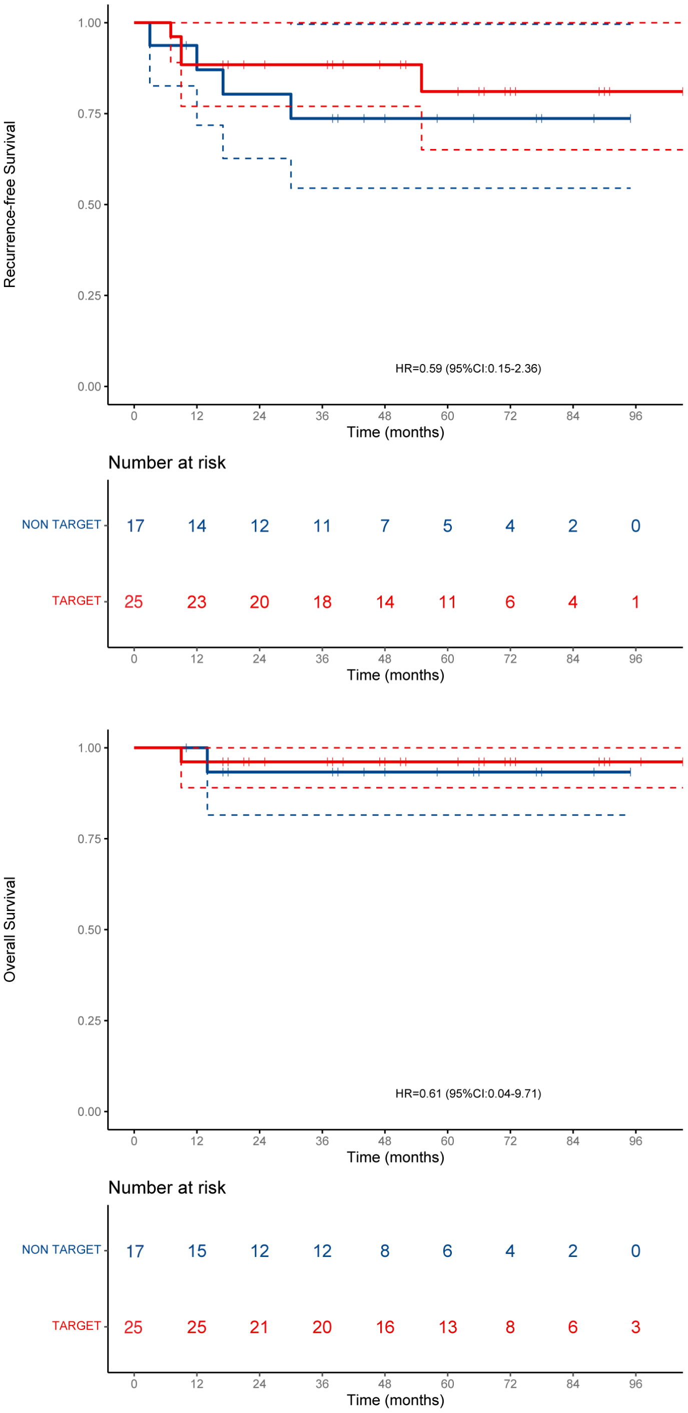

We have also analysed whether the time elapsed to reach plasma mitotane concentrations may have an effect on RFS, but we did not observe any statistically significant difference between the 13 patients who reached the target levels within 3 months and those who did it after 3 months, or never (Supplementary Figure 4).

Supplementary Figure 4. Survival of patients according to the time needed to reach plasma mitotane concentrations ≥14 mg/L. Kaplan-Meier plot for the risk of recurrence over 5 years according to the therapeutical levels. The red line indicates the patients who did reach the therapeutic levels within 3 months and the blue line indicates the patients who did not. The dashed lines represent the respective 95% Confidence Intervals.

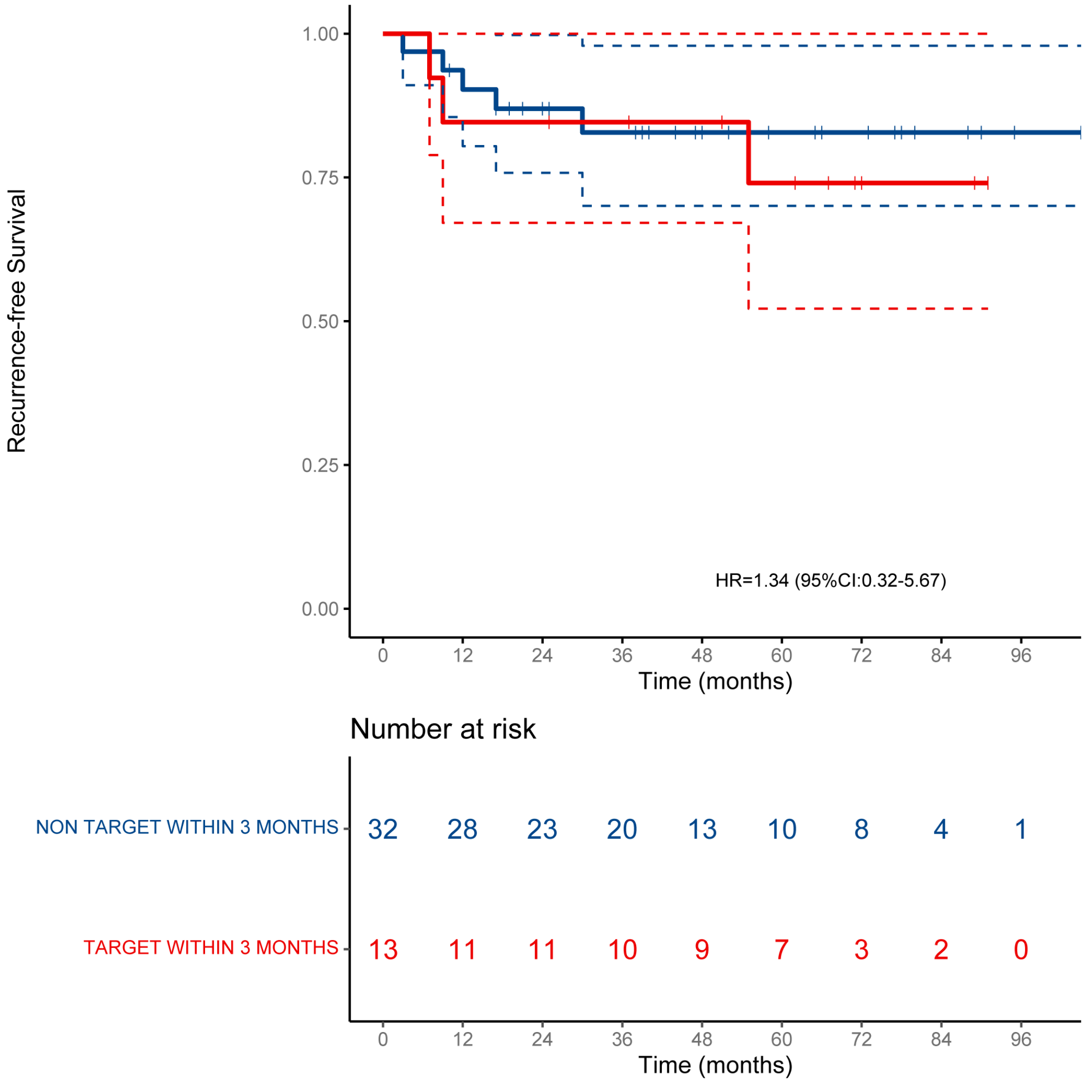

Measures of quality of life

Since there was at the time of study initiation no questionnaire specifically developed to evaluate the health-related quality of life (HRQoL) in patients with ACC, we collected data from a generic questionnaire routinely used for cancer diseases, the EORTC QLQ-C30 (European Organization for Research and Treatment of Cancer Quality of Life Questionnaire). EORTC QLQ-C30 is an integrated system for defining HRQoL in oncological patients that has been validated in several trials and is available in different languages [9]. The EORTC questionnaire consists of 30 questions (or items) that make up 3 multi-item scales that are assessed separately. The Functional scale includes 15 items divided among 5 sub-scales (Physical functioning, Role functioning, Emotional functioning, Cognitive functioning and Social functioning). The Symptom scale includes 3 multi-item sub-scales (Fatigue, Nausea and Vomiting, Pain) and 6 single-item sub-scales (Dyspnea, Insomnia, Appetite Loss, Constipation, Diarrhea, Financial difficulties). The Global Health status scale has no sub-scales and consists of only 2 items. The final scores of the EORTC QLQ-C30 questionnaire were calculated following the indications of the EORTC manual [10]. For both the functional scale and the symptom scale, each item was assigned a score from 1 to 4 while for the two items of the global health scale the possible score was between 1 and 7. For each of the three scales, the average score (raw score) of all the items composing every scale was calculated and then a linear transformation allowed to obtain a final score with a range between 0 and 100. Higher the score of the functional scale or of the global health scale, higher the QoL. On the contrary, higher the score of the symptom scale, greater the symptoms and lower the QoL [10]. The results of the comparison between the patients in the two arms, both at baseline and follow-up, are reported in the Supplementary Table 8.

Supplementary Table 8. Quality of Life according to the EORTC QLQ-C30 questionnaire. A, comparison between study arms. B, comparison between baseline and follow-up evaluations.

**A**	**Baseline**		***P*[1]**	**Follow-up**		***P*[1]**
	**MITOTANE ARM (N=20)**	**SURVEILLANCE ARM (N=18)**		**MITOTANE ARM (N=18)**	**SURVEILLANCE ARM (N=15)**	
Functional scale¶ *Mean ± SD*	69.1 ± 21.8	81.7 ± 16.4	0.204	58.8 ± 19.1	77.9 ± 19.1	0.015
Symptoms scale§ *Mean ± SD*	31.2 ± 21.9	14.4 ± 13.1	0.082	41.2 ± 20.2	19.3 ± 16.6	0.004
Global Health scale¶ *Mean ± SD*	62.2 ± 23.5	70.8 ± 19.9	0.382	45.0 ± 25.7	72.2 ± 19.3	0.004

**B**	**Change from Baseline to Follow-up**		
	**Mean difference**	**95%CI**	***P*[2]**
**MITOTANE ARM**			
Functional scale¶	10.31	-5.58 – 26.20	0.194
Symptoms scale§	-9.95	-26.31 – 6.41	0.222
Global Health scale¶	17.18	-2.08 – 36.44	0.078
**SURVEILLANCE ARM**			
Functional scale¶	-0.19	-17.5 – 17.1	0.982
Symptoms scale§	-1.63	-16.4 – 13.2	0.821
Global Health scale¶	-7.29	-25.4 – 10.88	0.412

[1] Wilcoxon-Mann-Whitney test
[2] Student's test
¶A higher value corresponds to a better quality of life.
§ A lower value corresponds to a better quality of life.

Supplementary Table 9. Centre-specific patient contribution in the ADIUVO Observational study.

Centre	Country	Patients randomized in the ADIUVO trial (n=91)	Patients not randomized and included in the ADIUVO Observational study (n=95)
Würzburg	Germany	18	25
Orbassano	Italy	17	8
Brescia	Italy	7	2
Paris	France	7	7
Villejuif	France	6	8
Munich	Germany	5	4
Zagreb	Croatia	5	5
Reims	France	4	0
Lyon	France	4	0
Milan	Italy	4	1
Montreal	Canada	4	0
Eindhoven	The Netherlands	2	2
Berlin	Germany	1	8
Birmingham	UK	1	3
Bordeaux	France	1	0
Ferrara	Italy	1	0
Grenoble	France	1	0
Rome	Italy	1	0
Toulouse	France	1	0
Turin	Italy	1	0
Rochester*	USA	0	10
Florence*	Italy	0	8
Sao Paolo*	Brasil	0	4

*These centres did not get ethical approval to participate in the interventional trial.

Supplementary Figure 5. Flow diagram of the ADIUVO Observational study.

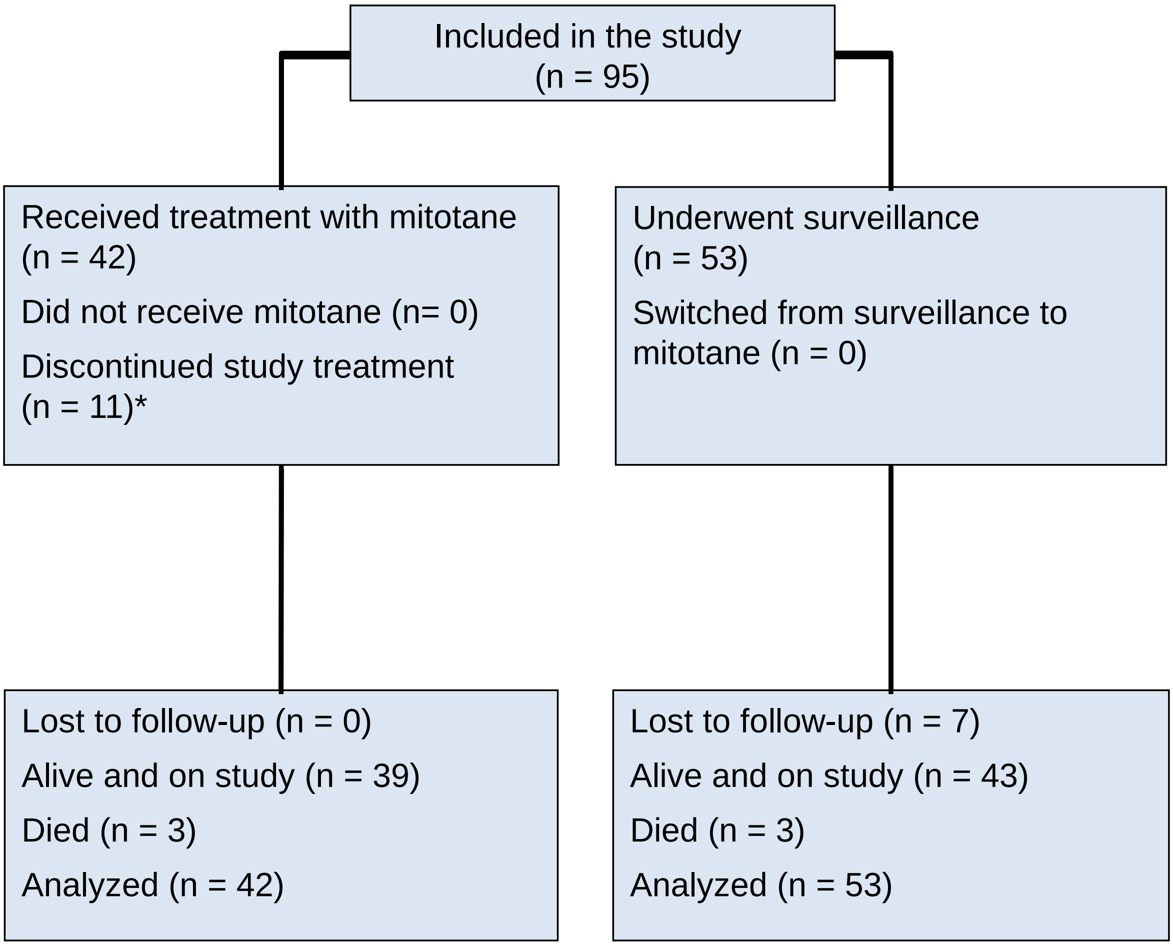

72% of the patients had follow-up visits every 3 months in the first 2 years of the study.

Additional statistical analyses - ADIUVO Observational study -

Baseline, demographic, and clinical characteristics of the patients with adrenocortical carcinoma (ACC) included in the ADIUVO Observational study were summarized before any adjustment using descriptive statistics, and were compared between study groups using the standardized mean difference as calculated according to Cohen d effect size [11]. A Cohen d effect size >0.1 denotes meaningful imbalance in the baseline covariates.

To mitigate the baseline differences between groups, the inverse probability weighting (IPW) approach based on propensity score (PS) was used [12-16]. The weights correspond to the inverse of the conditional PS of receiving treatment (mitotane). The PS for each patient was calculated as a probability from a logistic regression model that had treatment group as the dependent variable (mitotane vs. surveillance only) and the following baseline variables as independent covariates: age, Weiss score, tumour size, and Ki67 (as continuous variables), hormone secretion, and tumour stage (as dichotomized variables).

The semiparametric IPW Cox regression model was used to assess treatment effect differences on recurrence-free survival (RFS) and overall survival (OS). The estimates of mitotane effect were expressed as hazard ratios (HR) and 95% confidence intervals (95%CI) and Kaplan-Meier survival curves were displayed.

A total of 95 patients (42 in the mitotane group and 53 in the surveillance group) were included in the analysis.

The unweighted and the inverse probability-weighted characteristics of the patients according to the study groups are presented in the Supplementary Table 10.

The IPW-adjusted rate of 5-year RFS was 74% (95% CI, 58 to 94) in the mitotane group and 72% (95% CI, 59 to 88) in the surveillance group. The IPW-adjusted 5-year overall survival rate was 86% (95% CI, 72 to 100) in the mitotane group and 90% (95% CI, 79 to 100) in the surveillance group. (Supplementary Figure 6).

Supplementary Table 10. Baseline characteristics of patients in the ADIUVO Observational study. Unweighted and inverse probability-weighted characteristics of the patients of the ADIUVO Observational study included in the analysis, according to the treatment group.

	Unweighted			IPW		
	Mitotane	Surveillance	SMD	Mitotane	Surveillance	SMD
n	42	53		42<br>Sum of weights: 93	53<br>Sum of weights: 97	
Age at diagnosis (years), mean (SD)	46.6 (13.1)	54.4 (16.8)	0.253	48.2 (13.3)	48.1 (17.6)	0.009
Weiss score, median (IQR)	5 (2)	4 (2)	0.577	5 (2)	5 (3)	0.013
Tumor size in mm, mean (SD)	92.8 (45.9)	83.9 (47.1)	0.191	92.4 (44.9)	91.2 (50.4)	0.025
Hormone secretion, n (%)	23 (54.8)	30 (56.6)	0.037	54 (58.1)	56 (57.7)	0.024
Tumor stage, n (%)			0.089			0.015
Stage I-II	36 (85.7)	47 (88.7)		36 (88.6)	48 (88.1)	
Stage III	6 (14.3)	6 (11.3)		10 (10.7)	11 (11.3)	
KI67, median (IQR)	5 (5)	5 (5)	0.202	5 (4)	5 (7)	0.020

IPW: inverse probability-weighted; SMD: standardized mean difference; IQR: interquartile range

Supplementary Figure 6. Survival of patients in the ADIUVO Observational study. Kaplan-Meier estimates of recurrence-free-survival (Panel A) and overall survival (Panel B). Red line indicates the mitotane group and blue line the surveillance group.

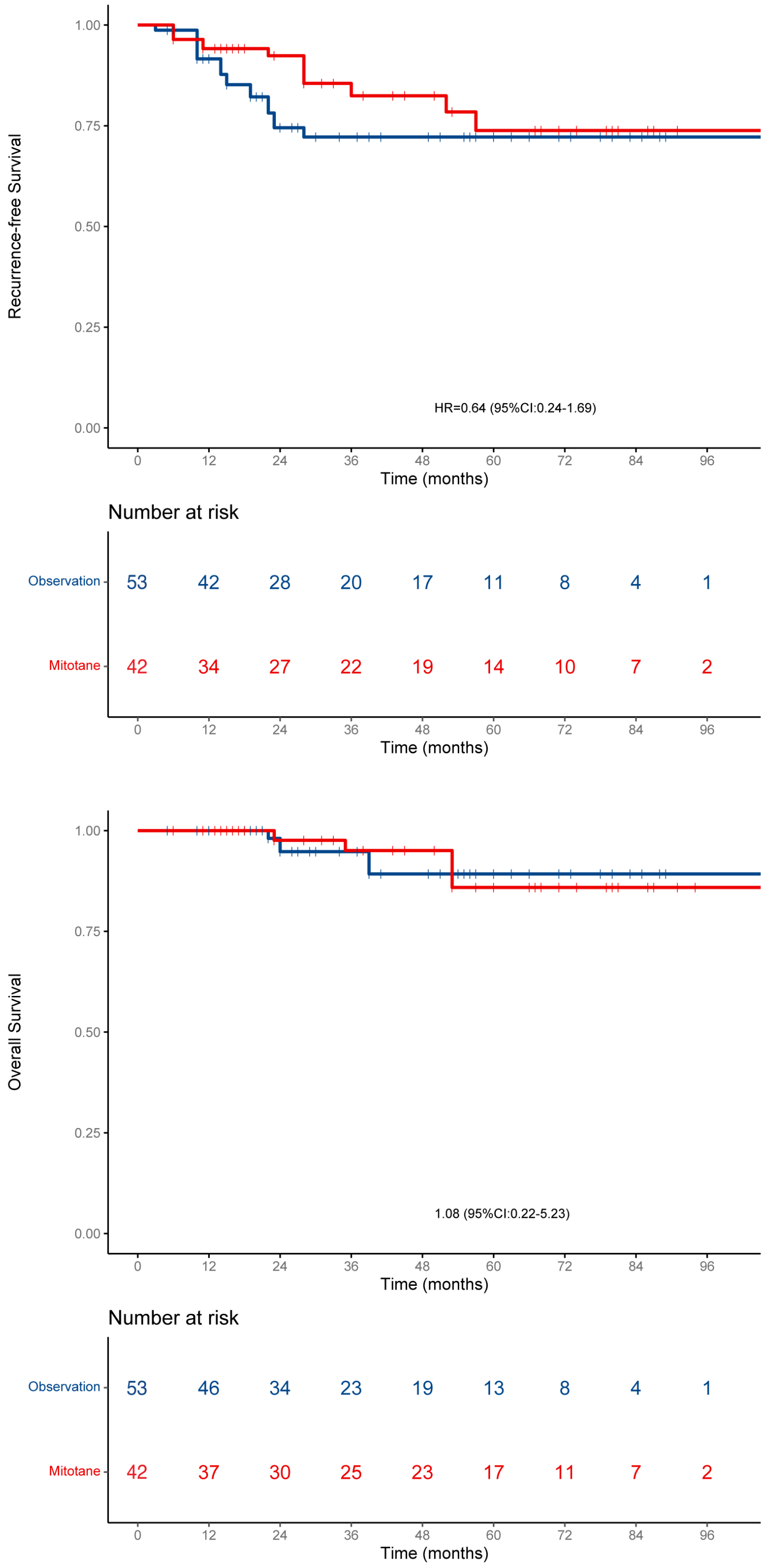

References to supplemental material

[1] Terzolo M, Angeli A, Fassnacht M, et al. Adjuvant mitotane treatment for adrenocortical carcinoma. N Engl J Med. 2007;356:2372-80.

[2] Haak HR, Hermans J, van de Velde CJ, et al. Optimal treatment of adrenocortical carcinoma with mitotane: results in a consecutive series of 96 patients. Br J Cancer. 1994;69:947-51.

[3] Baudin E, Pellegriti G, Bonnay M, et al. Impact of monitoring plasma 1,1-dichlorodiphenildichloroethane (o,p'DDD) levels on the treatment of patients with adrenocortical carcinoma. Cancer. 2001;92:1385-92.

[4] Hermsen IG, Fassnacht M, Terzolo M, et al. Plasma concentrations of o,p'DDD, o,p'DDA, and o,p'DDE as predictors of tumor response to mitotane in adrenocortical carcinoma: results of a retrospective ENS@T multicenter study. J Clin Endocrinol Metab. 2011;96:1844-51.

[5] Terzolo M, Baudin AE, Ardito A, et al. Mitotane levels predict the outcome of patients with adrenocortical carcinoma treated adjuvantly following radical resection. Eur J Endocrinol. 2013;169:263-70.

[6] Megerle F, Herrmann W, Schloetelburg W, et al. Mitotane Monotherapy in Patients With Advanced Adrenocortical Carcinoma. J Clin Endocrinol Metab. 2018;103:1686-95.

[7] Puglisi S, Calabrese A, Basile V, et al. Mitotane Concentrations Influence the Risk of Recurrence in Adrenocortical Carcinoma Patients on Adjuvant Treatment. J Clin Med. 2019;8.

[8] Puglisi S, Calabrese A, Basile V, et al. Mitotane Concentrations Influence Outcome in Patients with Advanced Adrenocortical Carcinoma. Cancers (Basel). 2020;12.

[9] Aaronson NK, Ahmedzai S, Bergman B, et al. The European Organization for Research and Treatment of Cancer QLQ-C30: a quality-of-life instrument for use in international clinical trials in oncology. J Natl Cancer Inst. 1993;85:365-76.

[10] Fayers PM, Aaronson NK, Bjordal K, et al. EORTC QLQ-C30 Scoring Manual (3rd edition). Brussels: EORTC. 2001.

[11] Cohen J. Statistical Power Analysis for the Behavioral Sciences. 2nd ed. Hillsdale, NJ: Lawrence Erlbaum Associates Publishers. 1988.

[12] Rosenbaum PR, Rubin DB. The central role of the propensity score in observational studies for causal effects. Biometrika. 1983;70:41-55.

[13] Rosenbaum PR. Model-based direct adjustment. J Am Stat Assoc. 1987;82:387-394.

[14] Cole SR, Hernán MA. Constructing inverse probability weights for marginal structural models. Am J Epidemiol. 2008;168:656-664.

[15] Austin PC. An introduction to propensity-score methods for reducing the effects of confounding in observational studies. Multivariate Behav Res. 2011;46:399-424.

[16] Austin PC, Stuart EA. Moving towards best practice when using inverse probability of treatment weighting (IPTW) using the propensity score to estimate causal treatment effects in observational studies. Stat Med. 2015;34:3661-3679.

<u>CLINICAL STUDY PROTOCOL</u>

<u>Title:</u> "EFFICACY OF ADJUVANT MITOTANE TREATMENT IN PROLONGING RECURRENCE-FREE SURVIVAL IN PATIENTS WITH ADRENOCORTICAL CARCINOMA AT LOW-INTERMEDIATE RISK OF RECURRENCE"

(Protocol ADIUVO version 2 15 July 2009)

On behalf of the European Network for the Study of Adrenal Tumors (ENS@T).

Coordinating Centers

Dipartimento di Scienze Cliniche e Biologiche Università di Torino

Medicina Interna I, ASO San Luigi, Orbassano (TO)

Massimo Terzolo, *MD* Tel. +39011 9026292; Fax +39011 9026992;

email: terzolo@usa.net

Oncologia Medica*,* ASO San Luigi, Orbassano (TO)

Alfredo Berruti, MD Tel. +39011 9026512; Fax +39011 9026992;

email: alfredo.berruti@gmail.com

Steering committee:

Study Chairs:

Massimo Terzolo

Medicina Interna

Dipartimento di Scienze Cliniche e Biologiche, Università di Torino

ASO San Luigi

Regione Gonzole, 10

10043 Orbassano

Italy

terzolo@usa.net

Alfredo Berruti

Oncologia Medica

Dipartimento di Scienze Cliniche e Biologiche, Università di Torino

ASO San Luigi

Regione Gonzole, 10

10043 Orbassano

Italy

alfredo.berruti@gmail.com

International Study Coordinator:

Martin Fassnacht

Dept. of Internal Medicine I

University of Wuerzburg

Josef-Schneider-Str. 2

D-97080 Wuerzburg

Germany

Fassnacht_m@medizin.uni-wuerzburg.de

European Study Coordinators:

Eric Baudin

Dept. of Nuclear Medicine and Encocrine Oncology

Institut Gustave-Roussy

Rue des Moulins 39

Villejuif

France

baudin@igr.fr

Harm Haak

Dept. of Internal Medicine

Maxima Medisch Centrum

5600 PD Eindhoven

The Netherlands

hrhaak@knmg.nl

Additional members of the Steering Committee

Bruno Allolio, Würzburg, Germany

Wiebke Arlt, Birminham, UK

Simon Aylwin, London, UK

Xavier Bertagna, Paris, France

Felix Beuschlein, Munich, Germany

Ashley Grossman, London, UK

Gabriel Dickstein, Haifa, Israel

Gary Hammer, Ann Arbor, USA

Barbara Jarzab, Poland

Andre Lacroix, Montreal, Canada

Dominique Maiter , Bruxelles, Belgium

Franco Mantero, Padua, Italy

Massimo Mannelli, Firence, Italy

Marcus Quinkler, Berlin

J Salvador, Pamplona, Spain

Britt Skogseid, Uppsala ,Sweden

Statistical Board

Responsible statistician:

Gianni Ciccone
Ufficio Trial, Centro Prevenzione Oncologica

ASO Molinette

10100 Torino

Italy

Tel +39011 6336857

gianni.ciccone@cpo.it

Statistical adviser

Hans-Helge Mueller,

Institute of Medical Biometry and Epidemiology

University of Marburg

35032 Marburg

Germany

+49-6421-286 6209

muellerh@med.uni-marburg.de

Data Manager:

Paola Perotti

Oncologia Medica

ASO San Luigi

10043 Orbassano

Italy

Tel +39011 011.9026643; Fax +39011 9026992;

oncotrial.sanluigi@gmail.com

Protocol committee:

Massimo Terzolo, Alfredo Berruti, Martin Fassnacht, Harm Haak, Eric Baudin, Gianni Ciccone

Independent Data Monitoring Committee

Ulrich Mannsmann, Germany

Bengt Simonssen, Sweden

Vincenzo Toscano, Italy

Table of Contents

1.0 Introduction
2.0 Rationale for performing the study
3.0 Study objectives
  3.1 Primary objective
  3.2 Secondary objectives
  3.3 Ancillary studies
4.0 Definition of study endpoints
  4.1 Primary end point
  4.2 Secondary end points
  4.3 Ancillary studies
5.0 Study population
  5.1 Inclusion criteria
  5.2 Exclusion criteria
6.0 Study design
7.0 Treatment schedule
8.0 Patient Informed Consent
9.0 Registration and randomization procedures
10.0 Drug information
11.0 Concomitant medication
12.0 Dose modification
13.0 Criteria for discontinuation of study drug administration
14.0 Trial procedures
  14.1 Initial clinical evaluation
  14.2 Evaluation visits
  14.3 Flow chart of examinations
15.0 Statistical Analysis
16.0 Safety assessment/ Reporting of Adverse Events
  16.1 Adverse Events
  16.2 Documentation and reporting of adverse events by investigator
  16.3 Immediate reporting by investigators to the Principal Investigator
17.0 Data handling

18.0 Study monitoring

19.0 Ethical considerations

20.0 References

Appendix 1: Data form for reporting the pathological examination (including the Weiss score)

Appendix 2: Data form for surgical report

Appendix 3: ENSAT staging classification

Appendix 4: Lysodren® Product Information

Appendix 5: ECOG index for Performance Status

Appendix 6: Quality of Life questionnaire (EORTC-QLQ-C30)

Appendix 7: NCI Common Toxicity Criteria

PROTOCOL SYNOPSIS

Title:	**EFFICACY OF ADJUVANT MITOTANE TREATMENT IN PROLONGING RECURRENCE-FREE SURVIVAL IN PATIENTS WITH ADRENOCORTICAL CARCINOMA AT LOW-INTERMEDIATE RISK OF RECURRENCE"**<br><br>**(Protocol ADIUVO version May 2009)**
**Study Rationale**	Adrenocortical carcinoma (ACC) is a very rare disease with a high risk of relapse after radical surgery. The efficacy of adjuvant mitotane treatment is suggested by a retrospective multicenter international study showing that postoperative mitotane treatment was associated with a significant reduction of the risk of relapse and death. However, these promising results need confirmation in a randomized prospective study. Caution should be adopted particularly in patients with low risk of disease relapse, in whom the benefit of therapy should be weighed against the side effects. Even if an adjuvant treatment seems justified in patients at high risk of relapse, a randomised prospective study is needed to assess whether such a treatment is efficacious in patients at low-intermediate risk.
Study Objectives	**Primary:**<br><br>To compare the efficacy of adjuvant mitotane treatment *vs* observation only in prolonging recurrence free survival (RFS) in patients with ACC at low-intermediate risk of recurrence after complete resection.<br><br>**Secondary:**<br><br>• Comparison of Overall Survival (OS)<br>• Comparison of time to recurrence (TTR)<br>• Comparison of disease-free survival (DFS)<br>• Comparison of quality of life<br>• Assessment of toxicity<br>• Assessment of the impact of mitotane plasma levels and time needed to reach the therapeutic interval on the efficacy of treatment<br>• Assessment of the efficacy of the mitotane administration on subgroups of patients stratified according to: type of hormone secretion, stage of disease, histopathologic characteristics.

Endpoints	**Primary:**<br><br>RFS, defined as the time between the date of randomization until documentation of any of the following failures (whichever occurs first):<br><br>-local or distant recurrence of ACC;<br><br>-death from any cause or completion of follow-up.<br><br>**Secondary:**<br><br>• OS, defined as the time interval between the date of randomization and the date of death from any cause<br>• TTR, defined as the time between the date of randomization until documentation of local or distant recurrence of ACC, or death from ACC<br>• DFS, defined as the time interval between the date of randomization until documentation of any relevant cancer disease, or death of any cause (whichever occurs first)<br>• Quality of life measured by EORTC-QLQ-C30<br>• Toxicity, graded according to the NCI-CTG criteria<br>• ·Incidence of second primary cancers.<br>• RFS, OS, TTR, and DSF in patients who do or do not achieve plasma mitotane concentrations ≥ 14 mg/L<br>• RFS, OS, TTR, and DSF between the 2 arms in patients subgroups stratified according to: type of hormone secretion, stage of disease, histopathologic characteristics.
Study Design	Prospective, randomized, controlled, open-label, multi-center phase III trial
Inclusion Criteria	• Histologically confirmed diagnosis of ACC<br>• Low-intermediate risk of relapse defined as:<br>• Stage I-III ACC (according to the ENS@T classification 2008)<br>• Microscopically complete resection, defined as no evidence of microscopic residual disease based on surgical reports, histopathology and post-operative imaging<br>• Ki 67 ≤ 10%<br>• Post-operative imaging (thoracic and whole abdominal CT with contrast medium or MRI) demonstrating no evidence of disease within 4 weeks before randomization<br>• Age ≥ 18 years<br>• ECOG performance status 0-2<br>• Adequate bone marrow reserve (neutrophils ≥ 1000/mm^3 and/or platelets≥ 80000/ mm^3)<br>• Ability to comply with the protocol procedures<br>• Written informed consent
Exclusion Criteria	• Time between primary surgery and randomization >3 months.<br>• Repeated surgery for recurrence of disease<br>• Persistence of autonomous adrenocortical hormone secretion following surgery

	• History of recent or active prior malignancy, except for cured non-melanoma skin cancer, cured *in situ* cervical carcinoma, or other treated malignancies where there has been no evidence of disease for at least three years<br>• Renal insufficiency (creatinine clearance < 40 ml/min) or liver insufficiency (serum bilirubin > 2 times the upper normal range and/or serum transaminases (AST, ALT) >3 times the upper normal range). Creatinine clearance may be calculated according to validated formulae (Crockoft's or MDRD)<br>• Pregnancy or breast feeding<br>• Previous or current treatment with mitotane or other antineoplastic drugs for ACC<br>• Previous radiotherapy for ACC<br>• Any other severe acute or chronic medical or psychiatric condition, or laboratory abnormality that would impart, in the judgment of the investigator, excess risk associated with study participation or study drug administration, or which, in the judgment of the investigator, would make the patient inappropriate for entry into this study.
Treatment administered	Patients will be randomly assigned to receive mitotane treatment or observational follow up only. The daily dose of the drug should be increased if plasma levels of the mitotane are below 14 mg/l. Reduction of mitotane dosage should be considered if plasma levels are over 20 mg/l or toxicity does occur. Mitotane will be administered until progression or unacceptable toxicity for a minimum of 2 years.
Concomitant treatments	All patients treated with mitotane will receive concomitant administration of glucocorticoids to prevent adrenal insufficiency. Fludrocortisone may be added depending on blood pressure, serum potassium levels and plasma renin activity/concentration.<br><br>The administration of any other anticancer agents including chemotherapy and active biological agents is NOT permitted.
Statistical analysis	The sample size is calculated using recurrence-free survival as the primary end point. The main statistical analysis of the primary end point will be based on the intention-to-treat (ITT) population. Based on the results of previous studies in patients with low-intermediate recurrence risk, a recurrence free survival rate after 2 years is estimated to be about 0.60 with surgery alone. The sample size is calculated to provide an 80% chance of detecting an increase of non- recurring patients at 2 years from 0.60 to 0.75. Taking 0.05 as the level of significance (alpha) and using a two-sided log rank test for analysis, it is calculated that approximately 184 patients should be randomized considering 4 years of accrual and 2 years of follow-up after entry of the last patient. Assuming a lost-to-follow-up rate of maximum of 10%, a total of 200 patients (100 per treatment arm) will be needed. Two interim analyses are planned at 20% and 60% of the expected events. Interim evaluations aimed at checking data quality and safety issues will be performed. The primary analysis on recurrence free survival will be conducted using the Kaplan-Meier method. The two-sided log rank test will be used to compare the survival times between the two arms. The Cox's proportional hazard model will be performed to estimate the hazard ratio (HR) with 95% confidence intervals. The final analysis should be conducted after 97 events with respect to the primary endpoint RFS have been

	observed. The primary analysis will be based on the ITT analysis set. However, a sensitivity analysis will be conducted on a per protocol analysis set. Toxicities will be compared between the two groups with usual statistical methods.
Sample size and Study duration	200 patients (100 per treatment arm) will be recruited. The duration of the study will comprise 6 years: recruitment period, 4 years and follow-up period, 2 years. At the end of the study patients will be asked to participate in a follow-up study to obtain data on long term OS.

1.0 INTRODUCTION

Adrenocortical carcinoma (ACC) is an extremely rare neoplasm characterized by a dismal prognosis since only 20-25% of patients with ACC survive for more than 5 years after diagnosis (1-4). Although the majority of patients have resectable disease at presentation (5) as many as 75-85% relapse after radical resection (3) and most of these patients finally die from ACC. This high recurrence rate of ACC has prompted investigators to consider the use of adjuvant therapy (5) and mitotane (o,p' DDD), an analogue of the insecticide DDT that has been widely employed in this setting (6), but debate continues as to whether mitotane may be beneficial as an adjuvant treatment following radical resection of the tumor (3, 4, 7, 8). However, the relevant studies reported such conflicting results that it is virtually impossible to conclude whether or not mitotane may be useful in the adjuvant setting. The main reason for these discrepancies is the difficulty in collecting adequate series of patients for a reliable assessment of treatment efficacy due to the low prevalence of ACC in the general population. Furthermore, it has to be considered that ACC is a heterogeneous disease and occasionally patients may survive more than 10 years experiencing exceptionally long relapse-free intervals (3, 9, 10). This observation points to the importance of stratifying patient cohorts for prognostic factors, implying that large numbers are needed for meaningful analysis.

The stage of the disease at diagnosis and a radical surgical resection are recognized as the strongest predictors of long-term survival (1, 3, 5, 11). Evidently, advanced ACC stage may contribute to a negative prognosis because it affects the chance of radical curative surgery. The histological algorithm of Weiss (12, 13) has a high predictive value for the diagnosis of ACC. However, there is limited evidence that it is predictive of long-term outcome in ACC (5, 14). The functional (hormone secretion) status of the tumor was usually found to be unrelated to prognosis although some reports indicated a longer survival in patients with androgen-secreting tumors (1, 3, 7, 10). In patients with advanced disease hypercortisolism may contribute to an unfavorable prognosis because of its negative effects on health status (15, 16). Mitotane treatment leads to control of hormone excess in the majority of patients due to its adrenolytic action. A recent analysis of the German ACC registry including more than 400 patients was able to establish for the first time prognostic factors that are independent of tumor stage in patients without distant metastases. This analysis demonstrated that the microscopically confirmed complete resection (R0) and the proliferation marker Ki67 (index ≤ 10%) are the strongest predictors for a relatively good prognosis (Fassnacht et al. unpublished results). However, even patients who fulfill these

criteria ("low/intermediate risk patients") have an unsatisfactory 5 year recurrence-free survival.
When analyzing the relevant literature on mitotane treatment in an adjuvant setting, amongst individuals with apparent curative resection, it is difficult to critically appraise the evidence of the efficacy of this approach since most available studies have limited power and lacked a concomitant control group of untreated patients with comparable baseline characteristics. Only four previous studies have reported the outcome of adjuvant mitotane treatment in series of more than 20 patients (10, 17-19).
In 2007 the results of a non-randomized controlled multicentric study conducted at several referral centers in Italy and Germany were published in the *New England Journal of Medicine* (20). The conclusions of the study were that adjuvant mitotane prolonged relapse-free survival in patients with radically resected adrenocortical carcinoma with limited toxicity. In this study, two independent groups of 55 Italian patients and 75 German patients who were left untreated after radical resection of ACC had a significantly higher risk of relapse than 47 Italian patients who were given mitotane adjuvantly (hazard ratio, 2.91; 95% confidence interval, 1.77 to 4.78; P<0.001 and hazard ratio, 1.97; 95% confidence interval, 1.21 to 3.20; P=0.005, respectively). A major advantage of this study compared to all previous studies is that mitotane was recommended on the basis of the treatment policy of the center independently on the characteristics of either tumors or patients. Conversely, it is very likely that in some of the previous series the treated patients were selected for unfavourable prognostic factors and this selection bias may have contributed to the lack of efficacy of adjuvant mitotane. However, the retrospective nature of the study warrants caution in the interpretation of the results.

The use of mitotane for patients with ACC has attracted many clinicians, but debate continues as to whether mitotane may be beneficial as an adjuvant treatment following radical resection of the tumor. Therefore, a collaborative prospective randomized study is crucial to establish the role of adjuvant mitotane treatment in patients with ACC after complete resection.

In patients at high risk of relapse, the evidence in the Terzolo paper (20) is considered by most authorities to represent strong evidence in favour of adjuvant therapy. Amongst patients at lower risk of relapse however, the benefit of mitotane has to be balanced against the unpleasant but reversible treatment side effects and also the irreversible development of adrenal insufficiency. Hypoadrenalism has a detrimental effect on quality of life and leads to an increased standardised mortality ratio (21). The recognized toxicity of mitotane is one

of the major limits to its use, the most common side effects being gastrointestinal and neurological. Gastrointestinal manifestations usually occur early in the course of treatment but subside afterwards and do not require discontinuation of the drug in most patients. Neurological toxicity is of central origin and reverses completely after mitotane withdrawal but may be very disabling. Luton and colleagues (18), Kasperlik-Zaluska and colleagues (19), Baudin and colleagues (22) reported that adverse effects were manageable and compliance was high. Conversely, severe and disabling toxicity was reported in other studies (2, 7). A different attitude towards the use of very high mitotane doses (more than 10 g/daily) may explain this discrepancy. More recently, the measurement of plasma mitotane was exploited aiming to avoid the severe toxicity associated with high concentrations (greater than 20 mg/L) (22, 23).

2.0 RATIONALE FOR PERFORMING THE STUDY

ACC is a very rare disease with a high risk of relapse after radical surgery. The efficacy of adjuvant mitotane treatment is supported by a retrospective multicenter international study showing that postoperative mitotane treatment was associated with a significant reduction of the risk of relapse and death. However, these promising results need confirmation in a randomized prospective study. Caution should be adopted particularly in patients with low/intermediate risk of disease relapse, in whom the benefit of therapy should be weighed against the side effects. Whereas adjuvant treatment is justified in patients at high risk of relapse, a randomised prospective study is needed to assess whether such a treatment is efficacious in low-risk patients.

The trial will be implemented on a multicentric and international basis following the fruitful experience of the FIRMACT study (First International Randomized trial in locally advanced and Metastatic Adrenocortical Carcinoma Treatment) an investigator-driven initiative that has been endorsed on a European scale by an international steering committee of which we are a part. This trial will take advantage of the existence of a number of collaborative networks that have been established in recent years in Europe (Collaborative group for Adrenocortical Carcinoma Therapy –COACT- European Network for the Study of Adrenal Tumors –ENS@T).

3.0 STUDY OBJECTIVES

3.1 Primary objective

To compare the efficacy of adjuvant mitotane treatment *vs observational follow-up only* in prolonging recurrence free survival (RFS) in patients with ACC after complete resection and low-intermediate risk for disease recurrence.

3.2 Secondary objectives

- Comparison of Overall Survival (OS)
- Comparison of time to recurrence (TTR)
- Comparison of disease-free survival (DFS)
- Quality of life assessment
- Assessment of toxicity
- Assessment of the impact of mitotane plasma levels and time needed to reach the therapeutic interval on the efficacy of treatment
- Assessment of the efficacy of the mitotane administration in predefined subgroups of patients stratified according to:
  - type of hormone secretion,
  - stage of disease,
  - histopathologic characteristics.

3.3 Ancillary studies

- Molecular biology studies will be subsequently performed on collected biomaterial. All centers will be asked to collect tumor samples, blood, saliva and urine samples from patients enrolled in the study. The tumor specimens will be reviewed by a reference pathologist for each country and archived. These samples will be used *a posteriori* for molecular and biological assessment to identify new prognostic and predictive parameters.

4.0 DEFINITION OF STUDY ENDPOINTS

4.1 Primary end point

RFS, defined as the time between the date of randomization until documentation of either of the following events (whichever occurs first):

- local or distant recurrence of ACC
- death from any cause.

4.2 Secondary end points

- OS, defined as the time interval between the date of randomization and the date of death from any cause.
- TTR, defined as the time between the date of randomization until documentation of local or distant recurrence of ACC, or death from ACC (whichever occurs first).
- DFS, defined as the time interval between the date of randomization until documentation of any relevant cancer disease, or death of any cause (whichever occurs first).
- Quality of life
  Quality of life will be measured by the standardized EORTC-QLQ-C30 (see Appendix 6) questionnaires. The EORTC-QLQ-C30 is a standardized questionnaire developed to assess the quality of life of cancer patients. It incorporates nine multi-item scales: five functional scales (physical, role, cognitive, emotional, and social); three symptom scales (fatigue, pain, nausea and vomiting); and a global health and quality-of-life scale. Two measures for the change are dealt with, the change in global quality of life at the time of first evaluation and the average change in global quality of life within the first two years or up to recurrence (whichever occurs first) both in reference to the baseline value.
- Toxicity. Safety measures that will be used in the study include physical examination and clinical laboratory tests (hematology, blood chemistries, and creatinine clearance). Patients will be rated for toxicity using the National Cancer Institute Common Toxicity Criteria (NCI CTC) scale (Appendix 7).
- Incidence of second primary cancers.
- RFS, TTR, DFS and OS in patients who achieve or not plasma mitotane concentrations $\geq$ 14 mg/L.

- RFS TTR, DFS and OS in patient subgroups stratified according to:
  - type of hormone secretion (cortisol secreting tumors *vs* purely sex-hormone secreting tumors *vs* non secreting tumors),
  - stage of disease (according to the ENSAT classification 2008, see Appendix 3),
  - histopathological characteristics (see Appendix 1).

5.0 STUDY POPULATION

5.1 Inclusion Criteria

- Histologically confirmed diagnosis of ACC according to Weiss system (see Appendix 1).
- Low-intermediate risk of relapse defined as:
  - Stage I-III (according to ENSAT classification 2008; see Appendix 3)
  - Microscopically complete resection, defined as no evidence of microscopic residual disease based on surgical reports, histopathology and post-operative imaging. Detailed pathological and surgical reports prepared according to guidelines detailed in appendix 1 and 2 should be available for assessment.
  - Ki 67 ≤ 10%
- Post-operative imaging (thoracic and whole abdominal CT with contrast medium or MRI) demonstrating no evidence of disease within 4 weeks before randomization
- Age ≥ 18 years
- ECOG performance status 0-2 (Appendix 5)
- Adequate bone marrow reserve (neutrophils ≥ 1000/mm^3 and/or platelets ≥ 80000/ mm^3)
- Ability to comply with the protocol procedures
- Written informed consent

5.2 Exclusion criteria

- Time between primary surgery and randomization > 3 months.
- Repeated surgery for recurrence of disease
- Persistence of autonomous adrenocortical hormone secretion following surgery

- History of prior malignancy, except for cured non-melanoma skin cancer, cured in situ cervical carcinoma, or other treated malignancies with no evidence of disease for at least three years
- Renal insufficiency (creatinine clearance < 40 ml/min) or liver insufficiency (serum bilirubin > 2 times the upper normal range and/or serum transaminases (AST/SGOT, ALT/SGPT) >3 times the upper normal range). Creatinine clearence may be calculted according to validated formulas (Crockoft’s or MDRD)
- Pregnancy or breast feeding
- Previous or current treatment with mitotane or other antineoplastic drugs for ACC
- Previous radiotherapy for ACC
- Any other severe acute or chronic medical or psychiatric condition, or laboratory abnormality that would impart, in the judgment of the investigator, excess risk associated with study participation or study drug administration, or which, in the judgment of the investigator, would make the patient inappropriate for entry into this study.

6.0 STUDY DESIGN

The study is designed as a prospective, randomized, open-label, controlled multi-center international phase III trial for patients with ACC after radical resection fulfilling the inclusion criteria and in whom no exclusion criteria are met. In a parallel group design, patients will be randomized 1:1 to receive adjuvant therapy with mitotane or observational follow-up only.

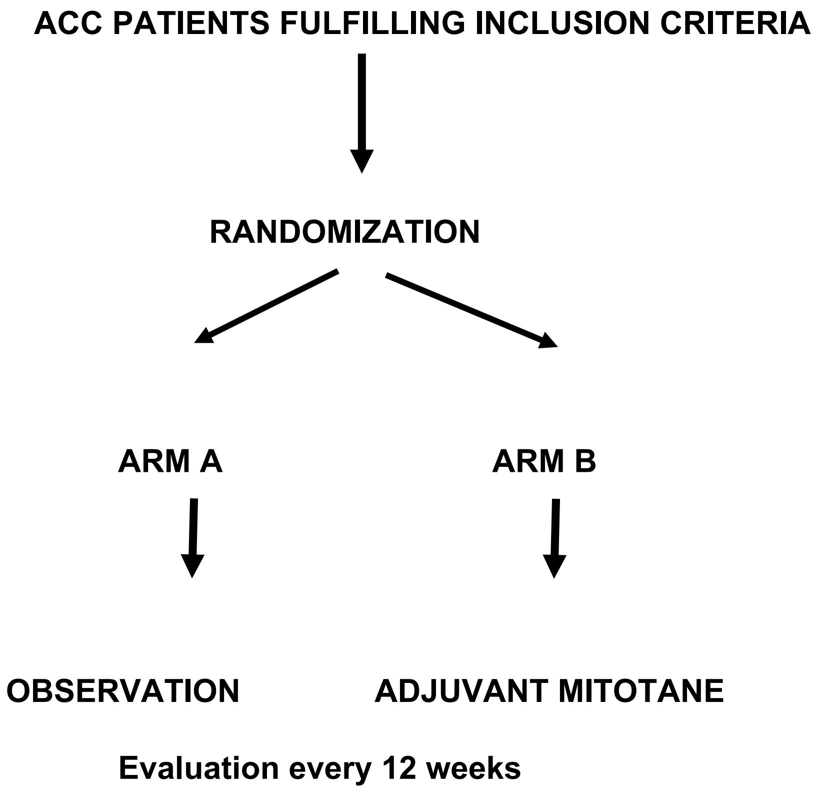

Study duration. In this study, 4 years of recruitment and 2 years of follow-up are expected. The study started in Italy in October 2008 and will start in other countries in 2009. The study will end approximately in the year 2015. The final report is expected in 2016.

7.0 Treatment schedule

Mitotane will be administered orally aiming to achieve a plasma level of 14-20 mg/l (or the maximum tolerated dose). Treatment should start as soon as possible after randomization (at latest 7 days thereafter). Analysis of circulating mitotane levels will be performed by a central laboratory (organized by the Lysosafe® service provided by HRA Pharma, France). Circulating plasma mitotane levels will be assessed every month until plateau; thereafter, monitoring will be performed every 3 months. Additional blood samplings may be performed at the discretion of the investigator.

The daily dose of the drug should be increased if plasma levels of the mitotane are below 14 mg/l. Reduction of mitotane dosage should be considered if plasma levels are over 20 mg/l or toxicity occurs. In the event of high levels in combination with side effects it might be necessary to temporarily discontinue treatment.

All patients will receive concomitant glucocorticoid replacement (see section 11.0).

The dosage scheme is the responsibility of the local investigator but a “high dose regimen” is recommended to avoid too long a delay in reaching plasma mitotane levels ≥ 14 mg/l. Briefly, mitotane will be administered at a starting dose of 1.5 g/day and increased providing good gastrointestinal tolerance on day 2 to 3 g/day, on day 3 to 4.5 g/day, and on day 4 to 6 g/day. A dose of 6 g/day will be administered until the first mitotane blood level is assessed. Further adjustment of dosage will be performed according to blood concentrations and tolerability.

In patients with reduced performance status or gastrointestinal or other problems a “low starting dose approach” may be considered: mitotane will be administered at a starting dose of 1 g/day and increased providing good gastrointestinal tolerance every 3-7 days by 0.5 g up to a total dose of 4.0 g/day and then adjusted according to blood concentrations and tolerability.

Mitotane toxicity will be assessed according to the NCI CTC criteria (Appendix 7).

Mitotane will be administered until ACC relapse, intolerable toxicity or for a period of 2 years. After the end of this period study treatment is stopped the local investigator is free to continue or stop mitotane according to his/her best clinical judgment and patient preference.

At the end of the study patients will be asked to participate in a follow-up study to obtain data on long term OS.

8.0 PATIENT INFORMED CONSENT

A consent document including patient information upon the nature, scope and possible consequence of the trial must have been approved by the Institutional Review Board. Patients amenable for inclusion in the trial will be given sufficient time to study the written information, as well as possibility to ask questions before signing the consent document.

9.0 REGISTRATION AND RANDOMIZATION PROCEDURES

The randomisation procedure will be performed online and implemented with the electronic CRF at the web-site *http://www.adiuvo-trial.org*. All centers participating in the study will receive a password to the internet-based database. After registering the patient by means of day of birth, patient's sex, participating center, and name of the local investigator responsible for the patient, confirming inclusion and exclusion criteria the patient will automatically be randomized to either mitotane treatment or observational follow-up only. The computer will immediately give the results of the randomization and this page should be printed and stored. The web-based randomization procedure, developed centrally by the Trial Center of the Piedmont Oncology Network, will be continuously accessible (24/24h a day). The procedure, after completing the entry of baseline data, will allocate each new patient to mitotane treatment or observational follow-up only using a randomization stratified for ACC stage (I-II vs. III), availability of FDG-PET during evalution procedures, and nationality of centers enrolling patients. This procedure developed by statisticians at the Trial Centre, will be completely concealed to researchers.

10.0 DRUG INFORMATION

The study drug (mitotane) will be provided by HRA Pharma (Paris, France) to centers in European countries, where HRA Pharma is the marketing authorisation holder. Centers in other countries have to get in contact with HRA Pharma or have to purchase commercially available mitotane (Lysodren®). Lysodren® product information is given in Appendix 4.

10.1 Biochemistry

Mitotane (1,1 dichloro-2(o-chlorophenyl)-2-(p-chloro-phenyl)ethane) is an isomer of the insecticide p,p'-DDD and a chemical congener of the insecticide DDT.

10.2 Pharmacokinetics

The oral bioavailability after oral intake is about 40 %. It is mainly metabolized in the liver. Mitotane is hydroxylated in the mitochondria at the β-carbon and further transformed into an acylchloride. It has been reported that the active metabolites cause toxicity by oxygen activation with superoxide formation or by covalent binding to specific proteins. The

elimination half-life of the parent compounds ranges between 18 and 159 days. There is a significant distribution of mitotane and its metabolites to fatty tissue. The renal excretion is about 10%.

10.3 Pharmaceutical Data

Formulation: mitotane is available as a 500 mg tablet.

Storage and Stability: the tablets may be stored at room temperature. Mitotane is insoluble in water and approximately 10 % soluble in oil or fat.

10.4 Human Toxicology

The most frequently observed adverse reactions consist of gastrointestinal, neurological, metabolic and skin reactions. The thresholds for gastrointestinal and neuropsychological toxicities have been reported to be about 5 mg/l and 15 mg/l, respectively. Frequently mitotane increases the hormone binding globulins, interferes with thyroid function and increases metabolism of glucocorticoids and other steroids.

Gastro-intestinal disorders.

These consist of anorexia, nausea or vomiting, and diarrhoea, occurring in about 80% of patients. Increases in hepatic gamma glutamyl transaminase levels are frequent and in most cases do not require withdrawal of the drug. However, serious hepatotoxicity has been described.

Nervous system disorders.

Adverse reactions affecting the central nervous system occur in 40% of the patients. These consist primarily of depression as manifested by lethargy and somnolence (25%) or dizziness and vertigo (15%). At high doses and after prolonged utilisation, cognitive impairment can occur which appears reversible at treatment cessation.

Metabolic disorders such as increases in plasma cholesterol or triglycerides are very common. Mitotane increases serum cholesterol mainly by increasing LDL-cholesterol.

Hormone alterations.

Mitotane strongly increases hormone binding globulins (e.g. cortisol-binding globulin, sex hormone binding globulin) leading to impaired bioavailability of the free hormones. Total thyroxine levels may be reduced as mitotane competes with endogenous thyroxine for thyroxine-bindin globulin binding sites. In some patients free thyroid hormone concentrations decrease and thyroxine replacement may become necessary.

Theoretically every patient will develop adrenal insufficiency requiring a high dose glucocorticoid replacement due to the increased metabolic clearance of glucocorticoids.

Gynecomastia is observed in a number of patients.

Skin and subcutaneous tissue disorders.

Skin toxicity has been observed in about 15% of the cases. The skin changes consist primarily of transient skin rashes, which do not seem to be dose related. In some instances, this side effect subsided while the patients were maintained on the drug without a change of dose.

Hematological toxicity.

Mild leukopenia, and prolonged bleeding times are relatively frequently observed. Anemia and thrombocytopenia may occasionally occur.

Eye disorders.

Visual blurring, diplopia, lens opacity, toxic retinopathy have been occasionally described;

Renal and urinary disorders

Hematuria, hemorrhagic cystitis, and albuminuria;

Cardiovascular disorders.

Orthostatic hypotension and hypertension have been reported as infrequent adverse effect; prolongation of QT interval has been described during mitotane treatment.

	**Undesirable effect (frequency)**		
**System Organ Class**	***Very common (≥ 1/10)***	***Common (≥ 1/100 , <1/10)***	***Rare (≥ 1/10,000, <1/1,000) or very rare (<1/10,000), including isolated reports***
Infections and infestations			Opportunistic mycoses
Blood and lymphatic system disorders	Mild leucopenia<br>Bleeding time prolonged	Thrombocytopenia<br>Anaemia	
Endocrine disorders	Adrenal insufficiency	Hypogonadism	Secondary Hypothyroidism
Metabolism and nutrition disorders	Hypercholesterolemia<br>Hypertriglyceridemia		
Nervous system and psychiatric disorders	Ataxia<br>Confusion<br>Myasthenia<br>Paresthesia<br>Anorexia<br>Asthenia<br>Vertigo<br>Fatigue	Polyneuropathy<br>Mental impairment<br>Movement disorder<br>Dizziness<br>Headache	
Eye disorders			Maculopathy<br>Retinal toxicity<br>Diplopia<br>Lens opacity<br>Visual impairment<br>Vision blurred
Cardiac and vascular disorders			Hypertension<br>Orthostatic hypotension<br>Flushing
Gastrointestinal disorders	Vomiting<br>Diarrhoea<br>Nausea		Salivary hypersecretion

	Epigastric discomfort		
Hepato-biliary disorders			Liver damage (hepatocellular/cholestatic/mixed)
Skin and subcutaneous tissue disorders	Skin rash		
Renal and urinary disorders			Haemorrhagic cystitis<br>Haematuria<br>Proteinuria
Reproductive system and breast disorders	Gynecomastia		
General disorders and administration site conditions			Hyperpyrexia
Laboratory findings	Serum GGT ↑<br>Plasma cholesterol ↑<br>Plasma triglycerides ↑<br>Liver transaminases ↑<br>Plasma hormone binding globulins (CBG, SHBG, TBG, vitamin D binding protein) ↑.<br>Disturbance of thyroid parameters (total or free T4↓)<br>Blood uric acid ↓		Albuminuria ↑

10.5 ***Interaction with other medicinal products and other forms of interaction***

Spironolactone: Mitotane should not be given in combination with spironolactone since this medicinal product may block the action of mitotane.

Warfarin and coumarin-like anticoagulants: Mitotane has been reported to accelerate the metabolism of warfarin by the mechanism of hepatic microsomal enzyme induction, leading to an increase in dosage requirements for warfarin. Therefore, physicians should closely monitor patients for a change in anticoagulant dosage requirements when administering mitotane to patients on coumarin-like anticoagulants.

Substances metabolised through cytochrome P450: Mitotane has been shown to have an inductive effect on cytochrome P450 enzymes. Therefore, the plasma concentrations of the products metabolised via cytochrome P450 may be modified. In the absence of information on the specific P450 isoenzymes involved, caution should be taken when co-prescribing active substances metabolised by this route such as, among others, anticonvulsants, rifabutin, rifampicin, statins, griseofulvin and St. John's Wort (*Hypericum perforatum*).

Medicinal products active on central nervous system: Mitotane can give rise to central nervous system undesirable effects at high concentrations. Although no specific information on pharmacodynamic interactions in the central nervous system is available, this should be considered when co-prescribing medicinal products with central nervous system depressant action.

Food and oil: Data with various mitotane formulations suggest that administration with food and/or oil enhance absorption.

11.0 CONCOMITANT MEDICATION

Concomitant medication and therapies deemed necessary for the supportive care of the patients are allowed. Due to the adrenolytic activity of mitotane, all patients treated with mitotane will receive concomitant administration of glucocorticoids to prevent adrenal

insufficiency. Due to an increased metabolic clearance rate of glucocorticoids by mitotane therapy, high-dose glucocorticoid replacement is typically required. Hydrocortisone or cortisone acetate are the steroids of choice and glucocorticoid replacement is monitored best with careful clinical assessment. A total daily dose of 50 mg hydrocortisone (divided as 20-20-10mg) or 75 mg cortisone acetate is typical and more may be needed. Fludrocortisone may be added depending on blood pressure, serum potassium levels and plasma renin activity/concentration at the discretion of the investigator.

Thyroxine replacement will be initiated if deemed necessary on the basis of clinical and laboratory findings.

The administration of any other anticancer agents including chemotherapy and active biological agents is NOT permitted.

12.0 DOSE MODIFICATION

Dose modification should be performed according to circulating drug levels aiming at concentrations between 14 and 20 mg/L (target range).

Since patients achieving very high mitotane blood concentration are at high risk of toxicity (neurological in particular), reduction of mitotane dosage should be considered if plasma levels are over 20 mg/l and mitotane concentrations should be monitored more frequently. In the event of high levels in combination with side effects it might be necessary to temporarily discontinue the treatment.

In patients developing toxicities despite mitotane levels within or below the therapeutic range the following recommendations should be considered.

12.1 Gastrointestinal toxicity

Gastrointestinal disorders are very frequent in patients under mitotane treatment and are generally reversible when the dose is reduced. Mild (grade I or II) gastrointestinal toxicity can be managed by use of palliative treatment and eventual dose reduction with subsequent re-introduction of previous dose in case of recovery. In case of severe gastrointestinal toxicity (grade III or IV) mitotane administration should be interrupted until symptom recovery. In both cases, subsequent mitotane dose should be adjusted as to limit as much as possible this adverse event.

In case of nausea and vomiting, partial adrenal insufficiency has to be considered and treated with higher doses of glucocorticoids. A potent antiemetic treatment (e.g. 5-HT_3 antagonists) is strongly recommended as concurrent treatment.

It should be noted that some of gastrointestinal symptoms, anorexia in particular, may also indicate early central nervous system toxicity.

12.2 Neurological toxicity

Since mitotane can frequently induce neurological toxicity, behavioural and neurological assessments should be made at regular intervals when continuous mitotane treatment is administered for a long time period. In addition, since sedation, lethargy, vertigo, and other central nervous system side effects can occur, ambulatory patients should be cautioned about driving, operating machinery, and other hazardous pursuits requiring mental and physical alertness. In case of mild-severe toxicity, mitotane administration will be stopped until recovery occurs and restarted at a lower dose (50-75% of the most recent dose).

12.3 Hematological toxicity

In case of severe leucocytopenia (neutrophils $<1000/mm^3$) and/or thrombocytopenia (platelets $< 40.000/mm^3$), mitotane administration will be stopped until recovery occurs and restarted at a lower dose (50-75% of the most recent dose).

Since mitotane can prolong the bleeding time, this should be analyzed when surgery is scheduled.

12.4 Hepatic toxicity

Mitotane therapy frequently leads to GGT elevation. Elevated GGT usually do not require mitotane dose modification unless exceedingly high levels are observed (>100-fold the upper normal range). In case of an increase of AST/SGOT, ALT/SGPT > 3-fold the upper normal range and > 3-fold of the individual baseline value, mitotane administration should be stopped until recovery and restarted with a lower dose (50-75% of the most recent dose).

Mitotane administration must temporarily be discontinued if the patient experienced other unexpected grade III toxicities.

13.0 CRITERIA FOR DISCONTINUATION OF STUDY DRUG ADMINISTRATION

Subjects may withdraw from the study treatment at any time and for any reason, without affecting their right to treatment by the investigator.

Administration of the study drug should be stopped if one or more of the following events occur:

• disease recurrence. However, mitotane (not as study drug) can be continued at the discretion of the investigator.

• unexpected NCI CTC grade IV toxicity related to drug administration

• refusal of the patient to continue treatment

• unsatisfactory compliance with study procedures.

The investigator must make every effort to continue follow-up after discontinuation or end of study treatment and contact subjects lost to follow-up.

14.0 TRIAL PROCEDURES

14.1 Initial clinical evaluation

The baseline evaluation should be performed as closely as possible to the beginning of the study and never more than one week before randomization.

Visit 0 includes:

- Complete history and physical examination
- Report on concomitant medications and treatments
- Review of surgical and pathological reports
  All tumor specimens will be reviewed *a posteriori* by a national reference pathologist, who should be identified before study initiation.

- Full blood count (CBC) with differential and platelet count
- Serum biochemistry profile including Na, K, Ca, creatinine clearance (measured or calculated), Glucose, AST/SGOT, ALT/SGPT, gamma Glutamyl Transpeptidase, Alkaline Phosphates, Total Bilirubin, Albumin, LDH, serum cholesterol, LDL cholesterol, HDL cholesterol, Triglycerides
- Endocrine assessment:
  - serum (and possibly salivary) cortisol, testosterone, DHEAS, androstenedione, 17-OH progesterone, aldosterone, estradiol (in males and postmenopausal women), ACTH, PRA or renin
  - FT3, FT4, TSH
- Collection of blood, saliva and 24-h urine samples for anxillary studies whenever possible
- Pregnancy test in women of childbearing potential
- Baseline ECG
- Baseline post-operative radiological imaging studies including thoracic + whole abdominal CT (MRI) with contrast medium should be performed no more than 4 weeks prior to randomization
- Quality of Life questionnaire (EORTC QLQ C-30)
- Written Informed Consent

After the completion of baseline procedures the patient will be randomized.

14.2 Evaluation visits

1) <u>Every 12 weeks after randomization (in both study arms) until recurrence</u>

- Physical exam
- Report of concomitant medications and treatments
- Report of side effects
- CBC with differential and platelet count
- Serum chemistry profile
- Endocrine assessment
- Plasma mitotane evaluation (in patients randomized in the mitotane arm only).
- Radiological imaging studies (MRI or CT with contrast medium) of thorax and whole abdomen.

- Quality of Life questionnaire (EORTC QLQ C-30)

After 6 months from randomization, FDG-PET or FDG-PET-CT should be performed in addition to the scheduled imaging evaluation (optional).

After two years, the time interval of visits will be increased to 6 months and after 5 years to 12 months.

After end of study treatment, the investigator must make every effort to continue follow-up of patients after the study end as long as possible and, theoretically, until the patient's death.

2) Additional *visits in the mitotane arm include:*

In all patients treated with mitotane, assessment of liver enzymes and plasma mitotane level will be performed after four weeks of treatment and then every month until mitotane concentrations plateau at target levels; thereafter, assessment will be performed every 3 months.

ECG will be performed every 6 months.

3) Follow-up visits

Although patients with recurrence will be not anymore evaluated within the study, these patients will be contacted every 6 months seeking information about their survival status. After the end of the study patients will be asked to participate in a follow-up study to get data on long term OS.

14.3 Flow chart of examinations

FLOW CHART OF EXAMINATIONS

	At baseline	every month[1]	every 3 months[2]
Physical Examination	*	*	*
Lab Tests	*	*	*
Endocrine Tests	*		*
ECG[3]	*		
Evaluation of adverse events	*	*	*
Abdominal + thoracic CT scan[4]	*		*
Quality of Life	*		*
Mitotane Monitoring[5]	*	*	*

[1] only in the mitotane arm until mitotane levels plateau

[2] time interval will be increased after 2 years to 6 months and after 5 years to 12 months

[3] ECG will be repeated every 6 months in patients on mitotane

[4] MRI can be performed as alternative

[5] only in the mitotane arm

15.0 STATISTICAL ANALYSIS

The sample size is calculated using the primary end point relapse-free survival. The main statistical analysis of the primary endpoint will be based on the intention-to-treat (ITT) population. Based on the results of previous studies and data from the German ACC Registry (Fassnacht, personal comunication), in patients with low relapse risk a recurrence free survival (RFS) rate after 2 years is estimated to be about 0.60 with surgery only. The sample size is calculated to provide an 80% chance of detecting an increase of non-recurring patients at 2 years from 0.60 to 0.75. According to O'Brien and Fleming sequential design with maximum three stages, taking 0.05 as the level of significance (alpha) and using a two-sided log rank test for analysis, it is calculated that 97 events in the primary endpoint RFS should be observed and approximately 184 patients should be randomized considering 4 years of accrual and 2 years of follow-up after entry of the last patient. Assuming a lost-to-follow-up rate of maximum 10%, a total of 200 patients (100 per treatment arm) will be needed. The two interim analyses will be carried out at 20% and 60% of the information rate (that under constant accrual rate correspond to about 2 years observation time and 19 expected events and, 4 years observation time and 58 expected events, respectively) Critical p-values for stopping are determined at p = 0.00001 for the first interim analysis, p = 0.01202 for the second and p = 0.0464 for the final analysis. Interim evaluations aimed at checking data quality and safety issues will be performed.

The primary analysis on RFS will be conducted as follows: for each treatment group, the RFS distribution and the median RFS time will be estimated using the Kaplan-Meier method. The two-sided logrank test will be used to compare the survival times between the two arms. The Cox's proportional hazard model will be performed to estimate the HR with 95% confidence intervals. To assess the proportional hazard assumption both graphical checks and statistical tests (as introducing into the model an interaction term between the treatment variable and a function of time) will be applied. The stratification employed to randomize the patients should prevent unbalances between the two arms, however an adjusted HR, standardised for the most important prognostic factors, will also be estimated. The final analysis should be conducted after 97 failures in the primary endpoint have been observed. The primary analysis will be based on the ITT analysis set. However, a sensitivity analysis will be conducted on a per protocol analysis set. The latter only serves to confirm the robustness of the results. Toxicities will be compared between the two arms with usual statistical methods, however, to take into account competitive risks (due to death or disease relapse), a cumulative incidence proportion of severe toxicities will be calculated using the

method described by Gooley et al. (28) and compared with Gray's test (29). Further analyses of the secondary endpoints are sensitivity analyses, or they are descriptive or explorative. Subgroup analyses will be performed by including in the Cox model interaction terms between the treatment and subgroup variables.

An extended follow-up period to get robust data on overall survival will be planned in more detail at the end of the study.

16.0 SAFETY ASSESSMENT / Reporting of Adverse Events

The collection of information on safety in clinical studies must be carried out efficiently and consistently so that analysis within and across studies is possible. Serious adverse events must be identified and reported rapidly so that potential hazards to patients can be identified and regulatory reporting requirements can be met. When an adverse event occurs, it should be graded according to the NCI-CTG criteria (version 3.0) (Appendix 7). Although the NCI-CTG criteria have been developed for discontinuous cytotoxic therapy, and is therefore not ideal for a continous oral therapy like mitotane, up to now no other generally accepted adverse event recording system has been established.

16.1 Adverse Events

Definition of Adverse Event

Any unfavorable symptom, sign, illness, or experience which develops or worsen in severity during the course of the study whether or not considered related to the medical treatment. Intercurrent illnesses or injuries should be regarded as adverse events. Abnormal results of diagnostic procedures including abnormal laboratory findings are considered to be adverse events if the abnormality:

• results in study withdrawal

• is associated with a serious adverse event

• is considered by the investigator to be of clinical significance.

Worsening of the disease under study will normally be measured by efficacy parameters, and should only be recorded as an AE if the outcome is serious or if specified in the protocol.

Definition of a Serious Adverse Events (SAE)

A SAE is any event that is:

• fatal

• life-threatening

• results in persistent or significant disability or incapacity

• requires or prolongs hospitalization

• a congenital anomaly or birth defect

• an important medical event.

Death as a consequence of worsening of the relapse will not be recorded as SAE.

Life-threatening means that the patient was at immediate risk of death from event as it occurred. It does not include an event that, had it occurred in more serious form, might have been life threatening (i.e. asymptomatic febrile neutropenia, laboratory findings of impaired liver function resulting in a dose modification or delay of therapy less than four weeks).

Requires patient hospitalisation or prolongation of existing hospitalisation should be defined as hospital admission required for treatment of the AE or occurred as a consequence of the event. Hospital admission for scheduled elective surgery would not be a SAE.

Important medical events are those which may not result in death or be immediately life threatening or result in hospitalization, but may jeopardize patients and may require intervention to prevent one of the other serious outcomes listed above. Examples of such events are intensive treatment for allergic bronchospasm, development of drug dependency, occurrence of a second malignancy.

All AEs which do not meet any of the criteria for serious should be regarded as **non-serious AE.**

16.2 Documentation and reporting of adverse events by investigator

AE and SAE have to be recorded in the CRF.

At each contact with the patient, the investigator must seek information on AEs by specific questioning and, as appropriate, by examination. Information on all AEs should be recorded promptly in the adverse event module of the CRF. All clearly related signs, symptoms, and

abnormal diagnostic procedures should be grouped together and recorded as a single diagnosis in the CRFs. The component parts of the diagnosis may be listed for verification.

All AEs occurring during the study period must be recorded. The clinical course of each event should be followed until resolution, stabilization or until it has been determined that study treatment or participation is not the cause. For SAEs, the Serious Adverse Event Report Form (SAERF) must also be completed. SAEs which are still ongoing at the end of the study period must be followed up to determine the final outcome.

Any SAE which occurs after the study period and is considered to be possibly or probably related to study treatment or study participation should be recorded and reported immediately.

The following definitions will be used to assess causality:

Code	Descriptor	Definition
5	Definite	The adverse event is *clearly related* to the investigational agent
4	Probable	The adverse event is *likely related* to the investigational agent
3	Possible	The adverse event *may be related* to the investigational agent
2	Unlikely	The adverse event is *doubtfully related* to the investigational agent
1	Unrelated	The adverse event is *clearly not related* to the investigational agent

Causality with the study drug will be considered as *it could not be ruled out* for codes 3, 4 and 5.

Toxicities will be recorded as they occur and graded according to the Common Toxicity Criteria (CTC) (Appendix 6). Toxicities that cannot be graded using the NCIC-CTG Common Toxicity Criteria will be graded as mild (asymptomatic), moderate (symtpomatic but not interfering significantly with function) or severe (causing significant interference with function).

16.3 Immediate reporting by investigators to the Principal Investigator

Any adverse event that is considered SERIOUS must be reported immediately (within 48 hours or, at the latest, on the following working day) by the investigator to the Study Chair

(Massimo Terzolo, Medicina Interna, ASO San Luigi, Regione Gonzole, 10, 10043 Orbassano, Italy, tel. +39 011 9026292, fax +39 011 9038655, e-mail terzolo@usa.net).

In addition, Serious Adverse Events that are considered *unexpected* according to the European Lysodren Summary of Product Characteristics and for which a *relation with the study drug* cannot be ruled out shall be reported in writing to the local regulatory authorities by the investigator according the national rules. The Study Chair will be responsible for reporting SAERF to the EMEA.

Relevant information about unexpected serious adverse events that are *fatal* or *life threatening* shall be recorded and reported as soon as possible but not later than *7 days* after first knowledge by the investigator. Relevant follow-up information is subsequently communicated within an additional 8 days.

All other unexpected serious adverse events shall be reported to the regulatory authorities and the ethical committee as soon as possible but not later than *15 days* after first knowledge by the investigator.

It is the responsibility of the Study Chair to inform all National Study Coordinators of Unexpected Serious Adverse Events that might have relevance for the conduct of the study and it is the responsibility of the National Study Coordinators to forward this information to the local investigators and to notify the local regulatory authorities and ethical committees.

17 Data handling and record keeping

17.1 Case Report Forms (CRF)

As used in this protocol, the term case report form (CRF) refers to the electronic data record. A CRF is required and should be completed for each included subject. The completed original CRFs will be archived for at least 10 years and should not be made available in any form to third parties, except for authorized representatives appropriate regulatory authorities. It is the investigator's responsibility to ensure completion and to review and approve all CRFs. At all times, the investigator has final personal responsibility for the accuracy and authenticity of all clinical and laboratory data entered on the CRFs. Subject source documents are the physician's subject records maintained at the trial site. In most cases, the source documents will be the hospital's or the physician's chart. In cases, in which the source documents are the hospital or the physician's chart, the information collected on the CRFs must match those charts.

In some cases, the CRF may also serve as the source document. In these cases, the investigator must prospectively document which items will be recorded in the source documents and for which items the CRF will stand as the source document.

17.2 Record retention

To enable evaluations and/or audits from an authorized monitoring crew or regulatory authorities, the investigator agrees to keep records, including the identity of all participating subjects (sufficient information to link records, eg, CRFs and hospital records), all original signed informed consent forms, copies of all CRFs, serious adverse event forms, source documents, and detailed records of treatment disposition. The records should be retained by the investigator according to ICH, local regulations, or as specified in the Clinical Study Agreement, whichever is longer.

If the investigator relocates, retires, or for any reason withdraws from the trial, the principal investigator should be prospectively notified. The trial records must be transferred to an acceptable designee, such as another investigator, another institution, or to the principal investigator.

18 Study monitoring and auditing

Standard monitoring and auditing procedures will be followed according ICH-GCP.

18.1 Study monitoring

The study will be monitored centrally by the Data Management Center in Orbassano (Torino). The responsible Data Manager will be not involved in patient care and will function as independent Monitor. He/she will review all e-CRF within 4 weeks. By frequent communications (letter, telephone, e-mail), the monitor will ensure that the investigations is conducted according to the protocol design and regulatory requirements. In addition, monitoring will be done by personal visit monitors (site monitoring), which are not involved in patient care of the study patients. The site monitoring will review the patient's informed consent, the case report forms, and source documents.
The investigator and institution will allow monitors and appropriate regulatory authorities direct access to source documents to perform this verification. It is important that the investigator(s) and their relevant personnel are available during the monitoring visits and possible audits or inspections and that sufficient time is devoted to the process.

18.2 Source data verification and on-site audits

The Data Monitors, the members of the Independent Data Monitoring Committee, regulatory authorities, and member of the independent Ethic Committees may request access to all source documents, case report forms, and other study documentation for on-site audit or inspection. Direct access to these documents must be guaranteed by the principal investigator and the local investigators, who must provide support at all time for these activities.

19 Ethical considerations

19.1 Independent Ethics Committee (IEC) / Institutional Review Boards (IRB) and Health Authorities

It is the responsibility of the investigator to have prospective approval of the trial protocol, protocol amendments, informed consent forms, and other relevant documents, eg,

advertisements, if applicable, from the IEC/IRB and the national Health Authorities. All correspondence with the IEC/IRB should be retained in the Investigator File.

19.2 Ethical conduct of the trial

The trial will be performed in accordance with the protocol, International Conference on Harmonization Good Clinical Practice guidelines, and applicable local regulatory requirements and laws.

19.3 Subject information and consent

A consent document including patient information upon the nature, scope and possible consequence of the trial must have been approved by the Independent Ethics Committee. The informed consent form must be in compliance with ICH GCP, local regulatory requirements, and legal requirements. Patients amenable for inclusion in the trial will be given sufficient time to study the written information, as well as possibility to ask questions before signing the consent document.

The investigator will obtain written informed consent from each subject or the subject's legally acceptable representative before any trial-specific activity is performed. The investigator will retain the original of each subject's signed consent form.

20.0 REFERENCES

1) Wajhenberg B, Albergaria PM, Medonca B, et al. Adrenocortical carcinoma: clinical and laboratory observations. Cancer 2000;88:711-736.

2) Dackiw AP, Lee JE, Gagel RF, Evans DB. Adrenal cortical carcinoma. World J Surg 2001;25:914-926.

3) Allolio B, Fassnacht M. Adrenocortical carcinoma: clinical update. J Clin Endocrinol Metab 2006;60:273-287.

4) Libe´ R, Fratticci A, Bertherat J. Adrenocortical cancer: pathophysiology and clinical management. Endocrine-Related Cancer 2007; 14:13–28.

5) Schteingart DE, Doherty GM, Gauger PG, et al. Management of patients with adrenal cancer: recommendations of an international consensus conference. Endocrine-Related Cancer 2005; 12:667–680.

6) Hahner S & Fassnacht M. Mitotane for adrenocortical carcinoma treatment. Current Opinion on Investigational Drugs 2005; 6: 386–394.

7) Pommier RF, Brennan MF. An eleven-year experience with adrenocortical carcinoma. Surgery 1992;112:963-970.

8) Huang H, Fojo T. Commentary: adjuvant mitotane for adrenocortical cancer—a recurring controversy. J Clin Endocrinol Metab 2008;93: 3730–3732.

9) Schteingart DE, Motazedi A, Noonan RA, Thompson NW. Treatment of adrenal carcinomas. Arch Surg 1982;117:1142-1146.

10) Venkatesh S, Hickey RC, Sellin RV, Fernandez JF, Samaan NA. Adrenal cortical carcinoma. Cancer 1989;64:765-769.

11) Bodie B, Novick AC, Pontes JE, et al. The Cleveland Clinic experience with adrenal cortical carcinoma. J Urol 1989;141:257-260.

12) Weiss LM. Comparative histologic study of 43 metastasizing and nonmetastasizing adrenocortical tumors. American Journal of Surgical Pathology 1984; 8:163-169.

13) Weiss LM, Medeiros LJ, Vickery AL, Jr. Pathologic features of prognostic significance in adrenocortical carcinoma. American Journal of Surgical Pathology 1989; 13:202-206.

14) Stojadinovic A, Ghossein RA, Hoos A, et al. Adrenocortical carcinoma: clinical, morphologic, and molecular characterization. J Clin Oncol 2002;20:941-950.

15) Abiven G, Coste J, Groussin L, et al. Clinical and biological features in the prognosis of adrenocortical cancer: poor outcome of cortisol-secreting tumors in a series of 202 consecutive patients. J Clin Endocrinol Metab 2006; 91:2650–2655.

16) Berruti A, Terzolo M, Sperone P, et al. Etoposide, doxorubicin and cisplatin plus mitotane

in the treatment of advanced adrenocortical carcinoma: a large prospective phase II trial. Endocr Relat Cancer 2005;12:657-666.
17) Icard P, Goudet P, Charpenay C, et al. Adrenocortical carcinomas: surgical trends and results of a 253-patient series from the French Association of Endocrine Surgeons study group. World J Surg 2001;25:891-897.
18) Luton JP, Cerdas S, Billaud L, et al. Clinical features of adrenocortical carcinoma, prognostic factors, and the effect of mitotane therapy. N Engl J Med 1990;322:1195-2001.
19) Kasperlik-Zaluska AA, Migdalska BM, Zgliczynski S, Makowska AM. Adrenocortical carcinoma. A clinical study and treatment results of 52 patients. Cancer 1995;75: 2587-2591.
20) Terzolo M, Angeli A, Fassnacht M et al. Adjuvant mitotane treatment for adrenocortical carcinoma. N Engl J Med 2007; 7; 356 (23): 2372-80.
21) Arlt W. The approach to the adult with newly diagnosed adrenal insufficiency. J Clin Endocrinol Metab. 2009; 94(4):1059-67.
22) Baudin E, Pellegriti G, Bonnay M, et al. Impact of monitoring plasma 1,1-dichlorodiphenildichloroethane (o,p'DDD) levels on the treatment of patients with adrenocortical carcinoma. Cancer 2001;92:1385-1392.
23) Haak HR, Hermans J, van de Velde CJ, et al. Optimal treatment of adrenocortical carcinoma with mitotane: results in a consecutive series of 96 patients. Br J Cancer 1994;69: 947-951.
24) Fassnacht M, Johanssen S, Quinkler M, et al. for the German Adrenocortical Carcinoma Registry Group and the European Network for the Study of Adrenal Tumors Members of the German ACC Registry Group 2008 Limited prognostic value of the 2004 International Union Against Cancer staging classification for adrenocortical carcinoma: proposal for a Revised TNM Classification. Cancer. 2009;115(2):243-50

Appendix 1. Pathological report

General information

- Patient identification:
- Date of surgery:
- Name of pathologist:

Pathological examination

Tumor of □ right □ left adrenal

Macroscopic investigation

Weight of adrenalectomy specimen…………….. g

Size of tumor …… cm

Margin of tumor □ sharp, □ irregular

□ Necrosis, □ Hemorrhage, □ Scars

Microscopic investigation

peritumoral adrenal cortex: □ normal, □ atrophic, □ hyperplastic, □ tumor invasion

Resection status (mandatory):

□ R0 □ R1 □ R2

□ RX (resection status is not determined, please specify: ____)

Weiss score (mandatory)

Criterion	Degree/Definition	Present*
Nuclear atypia	moderate to strong	
Mitoses	> 5 / 50 HPF	
Atypical mitoses	Present	
Spongiocytic tumor cells	< 25 % of tumor volume	
Architecture	Diffuse pattern	
Venous invasion	tumor cells within lumen	
Sinus invasion	tumor cells within lumen	
Capsular invasion	nests of tumor cells within the capsule	
Necroses	Area of > 2 HPF in diameter	
**TOTAL**		

*** each present criterion will be counted with a value of 1 leading to a maximum total score of 9**

Ki67 assessment: ____% positive cells (mandatory; for instructions see below)

**Immunostaining /molecular markers (optional)***			
Marker	**Tumor**	**adrenal cortex**	**adrenal medulla**
Keratin KL 1			
Epithelial membrane antigen			
Vimentin			

Chromogranin A			
Synaptophysin			
Neuron specific enolase			
p 53 protein			
Melan A			
Inhibin			
Cathepsin B			
Loss of heterozygosity for 17p13			
IGF-II overexpression			

* (+) very weak or uncertain; + weak or single cells; ++ moderate or 10 - 50 % positive cells; +++ strong or > 50 % pos. cells

Final Diagnosis:

□ adrenocortical adenoma

□ adrenocortical carcinoma

□ metastasis in adrenal

□ pheocromocytoma

□ Others: ________________________

Instructions for Ki67 assessments

- Select at low power view the tumor areas having the highest labeling density for Ki67.
- Only unequivocal staining of the nucleus should be interpreted as positive.
- Use a high magnification (400x) for quantification.
- Either: Estimate the percentage of KI67 positive nuclei by analyzing 50 high power fields
- Or: Estimate the percentage of Ki67 positive nuclei by randomly counting 1000 cancer cells at high magnification in those areas

Appendix 2. Surgical report

- Patient identification
- Date of operation

General information

- Name of surgeon's involved (team effort):
- Procedure:
    - Conventional (open) approach
    - Laparoscopic approach:
        - Transabdominal
        - Retroperitoneal
- Duration of operation:
- Blood loss: _____________ml

Specific information

- Describe the tumor characteristics:
    - Size
    - Consistency: □ firm, □ cystic, □ weak, □ suspected regional lymph nodes
    - Liver palpation – metastases
    - Ingrowth
        - renal vein
        - caval vein
        - diaphragm
        - kidney
        - liver
        - spleen
        - mesentery bowel
        - bowel
        - abdominal wall
    - Macroscopic radical resection
    - Macroscopically most narrow resection boundary of the tumor: ____________mm

- Use of drain: □ yes □ no

- Rupture of the tumor? □ yes □ no

- Complications?

Appendix 3. ENSAT ACC staging classification 2008 (Ref. 24)

Stage	ENSAT 2008
I	T1, N0, M0
II	T2, N0, M0
III	T1-2, N1, M0
	T3-4, N0, M0
IV	T1-4, N0-1, M1

T1 tumor ≤ 5cm

T2 tumor > 5cm

T3 tumor infiltration in surrounding tissue (histologically proven)

T4 tumor invasion in adjacent organs or venous tumor thrombus in vena cava or renal vein

N0 no positive lymph nodes

N1 positive lymph nodes

M0 no distant metastases

M1 presence of distant metastases

Appendix 4. Lysodren® Product Information

1. NAME OF THE MEDICINAL PRODUCT

Lysodren 500 mg tablets

2. QUALITATIVE AND QUANTITATIVE COMPOSITION

Each tablet contains 500 mg of mitotane.

For a full list of excipients, see section 6.1.

3. PHARMACEUTICAL FORM

Tablet. White, biconvex, round, scored tablets.

4. CLINICAL PARTICULARS

4.1 Therapeutic indications

Symptomatic treatment of advanced (unresectable, metastatic or relapsed) adrenal cortical carcinoma. The effect of Lysodren on non-functional adrenal cortical carcinoma is not established.

4.2 Posology and method of administration

Treatment should be initiated and followed by a suitably experienced specialist.

Posology

Treatment in adults should be started with 2 - 3 g mitotane per day and increased progressively (e.g. at two-week intervals) until mitotane plasma levels reach the therapeutic window 14 – 20 mg/l. If it is urgent to control Cushing's symptoms in highly symptomatic patients, higher starting doses between 4 - 6 g daily could be necessary and daily dose increased more rapidly (e.g. every week). A starting dose higher than 6 g/day is generally not recommended.

Dose adjustments, monitoring and discontinuation

Dose adjustment is aimed to reach a therapeutic window (mitotane plasma levels between 14 and 20 mg/l) which ensures optimal use of Lysodren with acceptable safety. Indeed, neurologic toxicity has been associated with levels above 20 mg/l and therefore this threshold should not be reached. Weaker evidence has suggested that mitotane plasma levels above 14 mg/l may result in enhanced efficacy. Mitotane plasma levels higher than 20 mg/l may be associated with severe undesirable effects and offer no further benefit in terms of efficacy. Mitotane plasma levels should therefore be monitored in order to adjust the Lysodren dose and to avoid reaching toxic levels. Dosing should be individually adjusted based on mitotane plasma levels monitoring and clinical tolerance until mitotane plasma levels reach the therapeutic window 14 – 20 mg/l. The target plasma concentration is usually reached within a period of 3 to 5 months.

Mitotane plasma levels should be assessed after each dose adjustment and at frequent (e.g. every two weeks) intervals, until the optimal maintenance dose is reached. Monitoring should be more frequent (e.g. every week) when a high starting dose has been used. It should be taken into account that dose adjustments do not produce immediate changes in plasma levels of mitotane (section 4.4). In addition, because of tissue accumulation, mitotane plasma levels should be monitored regularly (e.g. monthly) once the maintenance dose has been reached. Regular monitoring (e.g. every two months) of mitotane plasma levels is also necessary after interruption of treatment. Treatment can be resumed when mitotane plasma levels will be rangedbetween 14 - 20 mg/l. Due to the prolonged half-life, significant serum concentrations may persist for weeks after cessation of therapy.

If serious adverse reactions occur, such as neurotoxicity, treatment with mitotane may need to be transiently interrupted. In case of mild toxicity, the dose should be reduced until the maximum tolerated dose is attained. Treatment with Lysodren should be continued as long as clinical benefits are observed. If no clinical benefits are observed after 3 months at optimal dose, treatment should be permanently discontinued.

Special populations

Paediatric patients

The experience in children is limited. The paediatric posology of mitotane has not been well characterised but appears equivalent to that of adults after correction for body surface. Treatment should be initiated at 1.5 to 3.5 g/m2/day in children and adolescents with the objective of reaching 4 g/m2/day. Mitotane plasma levels should be monitored as for adults, with particular attention when plasma levels reach 10 mg/l as a quick increase in plasma levels may be observed. Dose may be reduced after 2 or 3 months according to the mitotane plasma levels or in case of serious toxicity.

Hepatic impairment

Since mitotane is mainly metabolised through the liver, mitotane plasma levels are expected to increase if liver function is impaired. There is no experience in the use of mitotane in patients with hepatic impairment, so data are insufficient to give a dose recommendation in this group. The use of mitotane in patients with severe hepatic impairment is not recommended. In patients with mild or moderate hepatic impairment, caution should be exercised and monitoring of liver biochemistry should be performed. Monitoring of mitotane plasma levels is specially recommended in these patients
(see section 4.4).

Renal impairment

There is no experience in the use of mitotane in patients with renal impairment, so data are insufficient to give a dose recommendation in this group. The use of mitotane in patients with severe renal impairment is not recommended and, in cases of mild to moderate renal impairment, caution should be exercised. Monitoring of mitotane plasma levels is specially recommended in these patients (see section 4.4).

Elderly patients (≥ 65 years old)

There is no experience on the use of mitotane in elderly patients, so data are insufficient to give a dose recommendation in this group. Caution should be exercised and frequent monitoring of mitotane plasma levels is highly recommended.

Method of administration

The total daily dose may be divided in two or three doses according to patient's convenience. Tablets should be taken with water during meals containing fat-rich food (see section 4.5). Patients should be advised not to use any tablets showing signs of deterioration, and caregivers to wear disposable gloves when handling the tablets.

4.3 Contraindications

Hypersensitivity to the active substance or to any of the excipients

Lactation (see section 4.6)

Concomitant use with spironolactone (see section 4.5)

4.4 Special warnings and precautions for use

Before the initiation of the treatment: Large metastatic masses should be surgically removed as far as possible before starting mitotane treatment, in order to minimise the risk of infarction and haemorrhage in the tumour due to a rapid cytotoxic effect of mitotane.

Risk of adrenal insufficiency: All patients with non functional tumour and 75% of patients with functional tumour show signs of adrenal insufficiency. Therefore, steroid replacement may benecessary in these patients. Since mitotane increases plasma levels of steroid binding proteins, free cortisol and corticotropin (ACTH) determinations are necessary for optimal dosing of steroid substitution (see section 4.8).

Shock, severe trauma or infection: Mitotane should be temporarily discontinued immediately following shock, severe trauma or infection, since adrenal suppression is its prime action. Exogenous steroids should be administered in such circumstances, since the depressed adrenal gland may not immediately start to secrete steroids. Because of an increased risk of acute adrenocortical insufficiency, patients should be instructed to contact their physician immediately if injury, infection, or any other concomitant illness occurs. Patients should carry with them the Lysodren Patient Card provided with the package leaflet indicating that they are prone to adrenal insufficiency and that, in
case of emergency care, adequate precautionary measures should be taken.

Monitoring of plasma levels: Mitotane plasma levels should be monitored in order to adjust the mitotane dose, particularly if high starting doses are considered necessary. Dose adjustments may be necessary to achieve the desired therapeutic levels in the window between 14 and 20 mg/l and avoid specific adverse reactions (see section 4.2).

Hepatic or renal impairment: There are insufficient data to support the use of mitotane in patients with severe hepatic or renal impairment. In patients with mild or moderate hepatic or renal impairment, caution should be exercised and monitoring of mitotane plasma levels is particularly recommended (see section 4.2).

Mitotane tissue accumulation: Fat tissue can act as a reservoir for mitotane, resulting in a prolonged half-life and potential accumulation of mitotane. Consequently, despite a constant dose, mitotane levels may increase. Therefore, monitoring of mitotane plasma levels (e.g. every two months) is also necessary after interruption of treatment, as prolonged release of mitotane can occur. Caution and close monitoring of mitotane plasma levels are highly recommended when treating overweight patients.

Central nervous system disorders: Long-term continuous administration of high doses of mitotane may lead to reversible brain damage and impairment of function. Behavioural and neurological assessments should be made at regular intervals, especially when mitotane plasma levels exceed 20 mg/l (see section 4.8).

Bleeding time: Prolonged bleeding time has been reported in patients treated with mitotane and this should be taken into account when surgery is considered (see section 4.8).

Warfarin and coumarin-like anticoagulants: Physicians should closely monitor patients for a change in anticoagulant dose requirements when administering mitotane to patients on coumarin-like anticoagulants (see section 4.5).

Substances metabolised through cytochrome P450: Mitotane is a hepatic enzyme inducer and it should be used with caution in case of concomitant use of medicinal products influenced by hepatic enzyme induction (see section 4.5).

Women of childbearing potential: Women of childbearing potential must use effective contraception during treatment with mitotane (see section 4.6).

Paediatric patients: In children and adolescents, neuro-psychological retardation can be observed during mitotane treatment. In such cases, thyroid function should be investigated in order to identify a possible thyroid impairment linked to mitotane treatment.

4.5 Interaction with other medicinal products and other forms of interaction

Spironolactone: Mitotane must not be given in combination with spironolactone, since this active substance may block the action of mitotane (see section 4.3).

Warfarin and coumarin-like anticoagulants: Mitotane has been reported to accelerate the metabolism of warfarin through hepatic microsomal enzyme induction, leading to an increase in dose requirements for warfarin. Therefore, physicians should closely monitor patients for a change in anticoagulant dose requirements when administering mitotane to patients on coumarin-like anticoagulants.

Substances metabolised through cytochrome P450: Mitotane has been shown to have an inductive effect on cytochrome P450 enzymes. Therefore, the plasma concentrations of the substances metabolised via cytochrome P450 may be modified. In the absence of information on the specific P450 isoenzymes involved, caution should be taken when co-prescribing active substances metabolised by this route such as, among others, anticonvulsants, rifabutin, rifampicin, griseofulvin and St. John's wort (Hypericum perforatum).

Medicinal products active on central nervous system: Mitotane can cause central nervous system undesirable effects at high concentrations (see section 4.8). Although no specific information on pharmacodynamic interactions in the central nervous system is available, this should be borne in mind when co-prescribing medicinal products with central nervous system depressant action.

Fat-rich food: Data with various mitotane formulations suggest that administration with fat-rich food enhances absorption of mitotane.

Hormone binding protein: Mitotane has been shown to increase plasma levels of hormone binding proteins (e.g. sex hormone-binding globulin (SHBG) and corticosteroid-binding globulin (CBG)). This should be taken into account when interpreting the results of hormonal assays and may result in gynaecomastia.

4.6 Pregnancy and lactation

Pregnancy

Data on a limited number of exposed pregnancies indicate abnormalities on the adrenals of the foetus after exposure to mitotane. Animal reproduction studies have not been conducted with mitotane. Animal studies with similar substances have shown reproductive toxicity (see section 5.3). Lysodren should be given to a pregnant woman only if clearly needed and if the clinical benefit clearly

outweighs any potential risk to the foetus. Women of childbearing potential must use effective contraception during treatment and after discontinuation of treatment as long as mitotane plasma levels are detectable. The prolonged elimination of mitotane from the body after discontinuation of Lysodren should be considered.

Lactation

Due to the lipophilic nature of mitotane, it is likely to be excreted in breast milk. Breast-feeding is contraindicated while taking mitotane (see section 4.3) and after treatment discontinuation as long as mitotane plasma levels are detectable.

4.7 Effects on ability to drive and use machines

Lysodren has a major influence on the ability to drive and use machines. Ambulatory patients should be warned not to drive or use machines.

4.8 Undesirable effects

Safety data are based on literature (mainly retrospective studies). More than 80 % of patients treated with mitotane have shown at least one type of undesirable effect. Adverse reactions listed below are classified according to frequency and system organ class. Frequency groupings are defined according to the following convention: Very common (≥1/10), Common (≥1/100 to <1/10), Uncommon (≥1/1,000 to <1/100), Rare (≥1/10,000 to <1/1,000), Very rare (<1/10,000), Not known (cannot be estimated from the available data). Within each frequency grouping, undesirable effects are presented in order of decreasing seriousness.

Gastrointestinal disorders are the most frequently reported (10 to 100 % of patients) and are reversible when the dose is reduced. Some of these effects (anorexia) may constitute the hallmark of initial central nervous system impairment.

Nervous system undesirable effects occur in approximately 40 % of patients. Other undesirable centralnervous effects have been reported in literature such as memory defects, aggressiveness, central vestibular syndrome, dysarthria, or Parkinson syndrome. Serious undesirable effects appear linked to the cumulative exposure to mitotane and are most likely to occur when mitotane plasma levels are at 20 mg/l or above. At high doses and after prolonged utilization, brain function impairment can occur. Nervous system undesirable effects appear reversible after cessation of mitotane treatment and decrease in plasma levels (see section 4.4).

Skin rashes which have been reported in 5 to 25 % of patients do not seem to be dose related.

Leucopoenia has been reported in 8 to 12 % of patients.

Prolonged bleeding time appears a frequent finding (90 %): although the exact mechanism of such an effect is unknown and its relation with mitotane or with the underlying disease is uncertain, it should be taken into account when surgery is considered.

The activity of liver enzymes (gamma-GT, aminotransferase, alkaline phosphatase) is commonly increased. Autoimmune hepatitis has been reported in 7 % of patients with no other information on mechanism. Liver enzymes levels normalize when the mitotane dose is decreased. A case of cholestatic hepatitis has been reported. Therefore, the possibility of mitotane-induced liver damage cannot be excluded.

Paediatric patients

Neuro-psychological retardation may be observed during mitotane treatment. In such cases, thyroid function should be investigated in order to identify a possible thyroid impairment linked to mitotane treatment. Hypothyroidism and growth retardation may be also observed.

4.9 Overdose

Mitotane overdose may lead to central nervous system impairment especially if mitotane plasma levels are above 20 mg/l. No proven antidotes have been established for mitotane overdose. The patient should be followed closely, taking into account that impairment is reversible, but given the long half-life and the lipophilic nature of mitotane, it may take weeks to return to normal. Other effects

should be treated symptomatically. Because of its lipophilic nature, mitotane is not likely to be dialysable. It is recommended to increase frequency of mitotane plasma level monitoring (e.g. every two weeks) in patients at risk of overdose (e.g. in case of renal or hepatic impairment, obese patients or patients with a recent weight loss).

5. PHARMACOLOGICAL PROPERTIES

5.1 Pharmacodynamic properties

Pharmacotherapeutic group: Other antineoplastic agents, ATC code: L01XX23

Mechanism of action

Mitotane is an adrenal cytotoxic active substance, although it can apparently also cause adrenal inhibition without cellular destruction. Its biochemical mechanism of action is unknown. Available data suggest that mitotane modifies the peripheral metabolism of steroids and that it also directly suppresses the adrenal cortex. The administration of mitotane alters the extra-adrenal metabolism of cortisol in humans, leading to a reduction in measurable 17-hydroxy corticosteroids, even though plasma levels of corticosteroids do not fall. Mitotane apparently causes increased formation of 6-betahydroxycholesterol.

Clinical efficacy

Mitotane has not been studied in a comprehensive clinical development program. Available clinical information comes mainly from published data in patients with inoperable or metastatic adrenal carcinoma. In terms of overall survival, four studies conclude that mitotane treatment does not increase the survival rate whereas five find an increase in the survival rate. Among the latter, three studies find such an increase only in patients in whom mitotane plasma is above 14 mg/l. In terms of total or partial tumour and/or metastasis regression, eleven studies have shown some degree of improvement and sometimes occasional prolonged remissions. However, in several studies, the objective criteria for evaluating tumour response are missing or not reported. There are nevertheless some studies which provide accurate information on tumour regression or disappearance and demonstrate that the threshold of 14 mg/l appears necessary to induce an objective tumour regression. In addition, mitotane induces a state of adrenal insufficiency which leads to the disappearance of Cushing syndrome in patients with secreting adrenal carcinoma and necessitates substitution hormonotherapy.

Paediatric patients

Clinical information comes mainly from a prospective trial (n= 24 patients) in children and adolescents aged at diagnosis from 5 months to 16 years (median age: 4 years) who had an unresectable primary tumour or who presented a tumour recurrence or a metastasic disease; most of the children (75%) presented with endocrine symptoms. Mitotane was given alone or combined with chemotherapy with various agents. Overall, the disease-free interval was 7 months (2 to 16 months). There were recurrences in 40% of children; the survival rate at 5 years was 49%.

5.2 Pharmacokinetic properties

Absorption

In a study performed in 8 patients with adrenal carcinoma treated with 2 to 3 g daily of mitotane, a highly significant correlation was found between plasma mitotane concentration and the total mitotane dose. The target plasma mitotane concentration (14 mg/l) was reached in all patients within 3 to 5 months and the total mitotane dose ranged between 283 and 387 g (median value: 363 g). The threshold of 20 mg/l was reached for cumulative amounts of mitotane of approximately 500 g. In another study, 3 patients with adrenal carcinoma received Lysodren according to a precise protocol allowing fast introduction of a high dose if the product was well tolerated: 3 g (as 3 intakes) on day 1, 4.5 g on day 2, 6 g on day 3, 7.5 g on day 4 and 9 g on day 5. This dose of Lysodren was continued or decreased in function of side effects and plasma mitotane levels. There was a positive linear correlation between the cumulative dose of Lysodren and the plasma levels of mitotane. In two of the 3 patients, plasma levels of more than 14 mg/l were achieved within 15 days and in one of them levels above 20 mg/l were achieved within approximately 30 days. In addition, in both studies, in some patients, the plasma mitotane levels continued to rise despite maintenance or a decrease of the daily dose of mitotane.

Distribution

Autopsy data from patients show that mitotane is found in most tissues of the body, with fat as the primary site of storage.

Metabolism

Metabolism studies in man have identified the corresponding acid, 1,1-(o,p'-dichlorodiphenyl) acetic acid (o,p'-DDA), as the major circulating metabolite, together with smaller quantities of the 1,1-(o,p'-dichlorodiphenyl)-2,2 dichloroethene (o,p'-DDE) analogue of mitotane. No unchanged mitotane has been found in bile or in urine, where o,p'-DDA predominates, together with several of its hydroxylated metabolites. For induction with cytochrome P450, see section 4.5.

Excretion

After intravenous administration, 25% of the dose was excreted as metabolites within 24 hours. Following discontinuation of mitotane treatment, it is slowly released from storage sites in fat, leading to reported terminal plasma half-lives ranging from 18 to 159 days.

5.3 Preclinical safety data

Non-clinical data on the general toxicity of mitotane is limited.

Reproductive toxicity studies have not been performed with mitotane. However, dichlorodiphenyltrichlorethane (DDT) and other polychlorinated biphenyl analogues are known to have deleterious effects on fertility, pregnancy and development, and mitotane could be expected to share these properties.

The genotoxic and carcinogenic potential of mitotane has not been investigated.

6. PHARMACEUTICAL PARTICULARS

6.1 List of excipients

Maize starch

Microcrystalline cellulose (E 460)

Macrogol 3350

Silica colloidal anhydrous

6.2 Incompatibilities

Not applicable.

6.3 Shelf life

3 years.

After opening: 1 year.

6.4 Special precautions for storage

Store in the original container.

6.5 Nature and contents of container

Square opaque white HDPE bottle containing 100 tablets.

Pack size of 1 bottle.

6.6 Special precautions for disposal and other handling

This medicinal product should not be handled by persons other than the patient and his/her caregivers, and especially not by pregnant women. Caregivers should wear disposable gloves when handling the tablets. Any unused product or waste material should be disposed of in accordance with local requirements for cytotoxic medicinal products.

7. MARKETING AUTHORISATION HOLDER

Laboratoire HRA Pharma

15 rue Béranger

75003 Paris

France

8. MARKETING AUTHORISATION NUMBER(S)

EU/1/04/273/001

9. DATE OF FIRST AUTHORISATION/RENEWAL OF THE AUTHORISATION

28/04/2004

10. DATE OF REVISION OF THE TEXT

Detailed information on this medicinal product is available on the website of the European Medicines Agency (EMEA) http://www.emea.europa.eu/

Appendix 5. ECOG index for Performance Status

Eastern Cooperative Oncology Group (Zubrod-ECOG)[1,2]	
**Description**	**Grade**
Fully active, able to carry on all pre-disease activities without restriction.	0
Restricted in physically strenuous activity but ambulatory and able to carry out work of a light or sedentary nature e.g. light house work, office work.	1
Ambulatory and capable of all self care but unable to carry out any work activities. Up and about more than 50% of waking hours.	2
Capable of only limited self care, confirmed to bed or chair more than 50% of waking hours.	3
Completely disabled. Cannot carry on any self care. Totally confined to bed or chair.	4

1 Zubrod, C.G., et al. *Appraisal of Methods for the Study of Chemotherapy of Cancer in Man*. Journal of Chronic Diseases, **11**:7-33, 1960.

2 Oken, M.M., et al. *Toxicity and response criteria of the Eastern Cooperative Oncology Group*. Am J Clin Oncol (CCT) 5: 649-655, 1982

Appendix 6. Quality of Life questionnaire

EORTC QLQ-C30 (version 3)

We are interested in some things about you and your health. Please answer all of the questions yourself by circling the number that best applies to you. There are no "right" or "wrong" answers. The information that you provide will remain strictly confidential.

Please fill in your initials: |_|_|_|_|
Your birthdate (Day, Month, Year): |_|_|_|_|_|_|_|_|
Today's date (Day, Month, Year): 31 |_|_|_|_|_|_|_|_|

		Not at All	A Little	Quite a Bit	Very Much
1.	Do you have any trouble doing strenuous activities, like carrying a heavy shopping bag or a suitcase?	1	2	3	4
2.	Do you have any trouble taking a long walk?	1	2	3	4
3.	Do you have any trouble taking a short walk outside of the house?	1	2	3	4
4.	Do you need to stay in bed or a chair during the day?	1	2	3	4
5.	Do you need help with eating, dressing, washing yourself or using the toilet?	1	2	3	4

**During the past week:**		Not at All	A Little	Quite a Bit	Very Much
6.	Were you limited in doing either your work or other daily activities?	1	2	3	4
7.	Were you limited in pursuing your hobbies or other leisure time activities?	1	2	3	4
8.	Were you short of breath?	1	2	3	4
9.	Have you had pain?	1	2	3	4
10.	Did you need to rest?	1	2	3	4
11.	Have you had trouble sleeping?	1	2	3	4
12.	Have you felt weak?	1	2	3	4
13.	Have you lacked appetite?	1	2	3	4
14.	Have you felt nauseated?	1	2	3	4
15.	Have you vomited?	1	2	3	4

**During the past week:**	Not at All	A Little	Quite a Bit	Very Much
16. Have you been constipated?	1	2	3	4
17. Have you had diarrhea?	1	2	3	4
18. Were you tired?	1	2	3	4
19. Did pain interfere with your daily activities?	1	2	3	4
20. Have you had difficulty in concentrating on things, like reading a newspaper or watching television?	1	2	3	4
21. Did you feel tense?	1	2	3	4
22. Did you worry?	1	2	3	4
23. Did you feel irritable?	1	2	3	4
24. Did you feel depressed?	1	2	3	4
25. Have you had difficulty remembering things?	1	2	3	4
26. Has your physical condition or medical treatment interfered with your family life?	1	2	3	4
27. Has your physical condition or medical treatment interfered with your social activities?	1	2	3	4
28. Has your physical condition or medical treatment caused you financial difficulties?	1	2	3	4

For the following questions please circle the number between 1 and 7 that best applies to you

29. How would you rate your overall health during the past week?

1 2 3 4 5 6 7

Very poor Excellent

30. How would you rate your overall quality of life during the past week?

1 2 3 4 5 6 7

Very poor Excellent

Appendix 7. NCI Common Toxicity Criteria (CTC) version 3.0

The following table provides a selection of the NCI CTCAE (Version 3.0 published 9. August 2006) criteria. The full version may be reviewed on-line at the following NCI website: http://ctep.cancer.gov/reporting/ctc.html

ALLERGY/IMMUNOLOGY

		Grade				
**Adverse Event**	**Short Name**	**1**	**2**	**3**	**4**	**5**
Allergic reaction/ hypersensitivity (including drug fever)	Allergic reaction	Transient flushing or rash; drug fever <38°C (<100.4°F)	Rash; flushing; urticaria; dyspnea; drug fever ≥38°C (≥100.4°F)	Symptomatic bronchospasm, with or without urticaria; parenteral medication(s) indicated; allergy-related edema/angioedema; hypotension	Anaphylaxis	Death
REMARK: Urticaria with manifestations of allergic or hypersensitivity reaction is graded as Allergic reaction/hypersensitivity (including drug fever).						
ALSO CONSIDER: Cytokine release syndrome/acute infusion reaction.						
Allergic rhinitis (including sneezing, nasal stuffiness, postnasal drip)	Rhinitis	Mild, intervention not indicated	Moderate, intervention indicated	—	—	—
REMARK: Rhinitis associated with obstruction or stenosis is graded as Obstruction/stenosis of airway – *Select* in the PULMONARY/UPPER RESPIRATORY CATEGORY.						
Autoimmune reaction	Autoimmune reaction	Asymptomatic and serologic or other evidence of autoimmune reaction, with normal organ function and intervention not indicated	Evidence of autoimmune reaction involving a non-essential organ or function (e.g., hypothyroidism)	Reversible autoimmune reaction involving function of a major organ or other adverse event (e.g., transient colitis or anemia)	Autoimmune reaction with life-threatening consequences	Death
ALSO CONSIDER: Colitis; Hemoglobin; Hemolysis (e.g., immune hemolytic anemia, drug-related hemolysis); Thyroid function, low (hypothyroidism).						
Serum sickness	Serum sickness	—	—	Present	—	Death
NAVIGATION NOTE: Splenic function is graded in the BLOOD/BONE MARROW CATEGORY.						
NAVIGATION NOTE: Urticaria as an isolated symptom is graded as Urticaria (hives, welts, wheals) in the DERMATOLOGY/SKIN CATEGORY.						
Vasculitis	Vasculitis	Mild, intervention not indicated	Symptomatic, non-steroidal medical intervention indicated	Steroids indicated	Ischemic changes; amputation indicated	Death
Allergy/Immunology – Other (Specify, __)	Allergy – Other (Specify)	Mild	Moderate	Severe	Life-threatening; disabling	Death

BLOOD/BONE MARROW

		Grade				
**Adverse Event**	**Short Name**	**1**	**2**	**3**	**4**	**5**
Bone marrow cellularity	Bone marrow cellularity	Mildly hypocellular or ≤25% reduction from normal cellularity for age	Moderately hypocellular or >25 – ≤50% reduction from normal cellularity for age	Severely hypocellular or >50 – ≤75% reduction cellularity from normal for age	—	Death
CD4 count	CD4 count	<LLN – 500/mm^3<br><LLN – 0.5 x 10^9 /L	<500 – 200/mm^3<br><0.5 – 0.2 x 10^9 /L	<200 – 50/mm^3<br><0.2 x 0.05 – 10^9 /L	<50/mm^3<br><0.05 x 10^9 /L	Death
Haptoglobin	Haptoglobin	<LLN	—	Absent	—	Death
Hemoglobin	Hemoglobin	<LLN – 10.0 g/dL<br><LLN – 6.2 mmol/L<br><LLN – 100 g/L	<10.0 – 8.0 g/dL<br><6.2 – 4.9 mmol/L<br><100 – 80g/L	<8.0 – 6.5 g/dL<br><4.9 – 4.0 mmol/L<br><80 – 65 g/L	<6.5 g/dL<br><4.0 mmol/L<br><65 g/L	Death
Hemolysis (e.g., immune hemolytic anemia, drug-related hemolysis)	Hemolysis	Laboratory evidence of hemolysis only (e.g., direct antiglobulin test [DAT, Coombs'] schistocytes)	Evidence of red cell destruction and ≥2 gm decrease in hemoglobin, no transfusion	Transfusion or medical intervention (e.g., steroids) indicated	Catastrophic consequences of hemolysis (e.g., renal failure, hypotension, bronchospasm, emergency splenectomy)	Death
ALSO CONSIDER: Haptoglobin; Hemoglobin.						
Iron overload	Iron overload	—	Asymptomatic iron overload, intervention not indicated	Iron overload, intervention indicated	Organ impairment (e.g., endocrinopathy, cardiopathy)	Death
Leukocytes (total WBC)	Leukocytes	<LLN – 3000/mm^3<br><LLN – 3.0 x 10^9 /L	<3000 – 2000/mm^3<br><3.0 – 2.0 x 10^9 /L	<2000 – 1000/mm^3<br><2.0 – 1.0 x 10^9 /L	<1000/mm^3<br><1.0 x 10^9 /L	Death
Lymphopenia	Lymphopenia	<LLN – 800/mm^3<br><LLN x 0.8 – 10^9 /L	<800 – 500/mm^3<br><0.8 – 0.5 x 10^9 /L	<500 – 200 mm^3<br><0.5 – 0.2 x 10^9 /L	<200/mm^3<br><0.2 x 10^9 /L	Death
Myelodysplasia	Myelodysplasia	—	—	Abnormal marrow cytogenetics (marrow blasts ≤5%)	RAEB or RAEB-T (marrow blasts >5%)	Death
Neutrophils/granulocytes (ANC/AGC)	Neutrophils	<LLN – 1500/mm^3<br><LLN – 1.5 x 10^9 /L	<1500 – 1000/mm^3<br><1.5 – 1.0 x 10^9 /L	<1000 – 500/mm^3<br><1.0 – 0.5 x 10^9 /L	<500/mm^3<br><0.5 x 10^9 /L	Death
Platelets	Platelets	<LLN – 75,000/mm^3<br><LLN – 75.0 x 10^9 /L	<75,000 – 50,000/mm^3<br><75.0 – 50.0 x 10^9 /L	<50,000 – 25,000/mm^3<br><50.0 – 25.0 x 10^9 /L	<25,000/mm^3<br><25.0 x 10^9 /L	Death
Splenic function	Splenic function	Incidental findings (e.g., Howell-Jolly bodies)	Prophylactic antibiotics indicated	—	Life-threatening consequences	Death
Blood/Bone Marrow – Other (Specify, __)	Blood – Other (Specify)	Mild	Moderate	Severe	Life-threatening; disabling	Death

CARDIAC ARRHYTHMIA

Page 1 of 2

		Grade				
**Adverse Event**	**Short Name**	**1**	**2**	**3**	**4**	**5**
Conduction abnormality/ atrioventricular heart block – *Select*: – Asystole – AV Block-First degree – AV Block-Second degree Mobitz Type I (Wenckebach) – AV Block-Second degree Mobitz Type II – AV Block-Third degree (Complete AV block) – Conduction abnormality NOS – Sick Sinus Syndrome – Stokes-Adams Syndrome – Wolff-Parkinson-White Syndrome	Conduction abnormality – *Select*	Asymptomatic, intervention not indicated	Non-urgent medical intervention indicated	Incompletely controlled medically or controlled with device (e.g., pacemaker)	Life-threatening (e.g., arrhythmia associated with CHF, hypotension, syncope, shock)	Death
Palpitations	Palpitations	Present	Present with associated symptoms (e.g., lightheadedness, shortness of breath)	—	—	—
REMARK: Grade palpitations only in the absence of a documented arrhythmia.						
Prolonged QTc interval	Prolonged QTc	QTc >0.45 – 0.47 second	QTc >0.47 – 0.50 second; ≥0.06 second above baseline	QTc >0.50 second	QTc >0.50 second; life-threatening signs or symptoms (e.g., arrhythmia, CHF, hypotension, shock syncope); Torsade de pointes	Death
Supraventricular and nodal arrhythmia – *Select*: – Atrial fibrillation – Atrial flutter – Atrial tachycardia/Paroxysmal Atrial Tachycardia – Nodal/Junctional – Sinus arrhythmia – Sinus bradycardia – Sinus tachycardia – Supraventricular arrhythmia NOS – Supraventricular extrasystoles (Premature Atrial Contractions; Premature Nodal/Junctional Contractions) – Supraventricular tachycardia	Supraventricular arrhythmia – *Select*	Asymptomatic, intervention not indicated	Non-urgent medical intervention indicated	Symptomatic and incompletely controlled medically, or controlled with device (e.g., pacemaker)	Life-threatening (e.g., arrhythmia associated with CHF, hypotension, syncope, shock)	Death
NAVIGATION NOTE: Syncope is graded as Syncope (fainting) in the NEUROLOGY CATEGORY.						

CARDIAC ARRHYTHMIA

Page 2 of 2

		Grade				
**Adverse Event**	**Short Name**	**1**	**2**	**3**	**4**	**5**
Vasovagal episode	Vasovagal episode	—	Present without loss of consciousness	Present with loss of consciousness	Life-threatening consequences	Death
Ventricular arrhythmia – *Select*: – Bigeminy – Idioventricular rhythm – PVCs – Torsade de pointes – Trigeminy – Ventricular arrhythmia NOS – Ventricular fibrillation – Ventricular flutter – Ventricular tachycardia	Ventricular arrhythmia – *Select*	Asymptomatic, no intervention indicated	Non-urgent medical intervention indicated	Symptomatic and incompletely controlled medically or controlled with device (e.g., defibrillator)	Life-threatening (e.g., arrhythmia associated with CHF, hypotension, syncope, shock)	Death
Cardiac Arrhythmia – Other (Specify, __)	Cardiac Arrhythmia – Other (Specify)	Mild	Moderate	Severe	Life-threatening; disabling	Death

CARDIAC GENERAL

		Grade				
**Adverse Event**	**Short Name**	**1**	**2**	**3**	**4**	**5**
NAVIGATION NOTE: Angina is graded as Cardiac ischemia/infarction in the CARDIAC GENERAL CATEGORY.						
Cardiac ischemia/infarction	Cardiac ischemia/infarction	Asymptomatic arterial narrowing without ischemia	Asymptomatic and testing suggesting ischemia; stable angina	Symptomatic and testing consistent with ischemia; unstable angina; intervention indicated	Acute myocardial infarction	Death
Cardiac troponin I (cTnI)	cTnI	—	—	Levels consistent with unstable angina as defined by the manufacturer	Levels consistent with myocardial infarction as defined by the manufacturer	Death
Cardiac troponin T (cTnT)	cTnT	0.03 – <0.05 ng/mL	0.05 – <0.1 ng/mL	0.1 – <0.2 ng/mL	0.2 ng/mL	Death
Cardiopulmonary arrest, cause unknown (non-fatal)	Cardiopulmonary arrest	—	—	—	Life-threatening	—
REMARK: Grade 4 (non-fatal) is the only appropriate grade. CTCAE provides three alternatives for reporting Death:<br>1. A CTCAE term associated with Grade 5.<br>2. A CTCAE 'Other (Specify, __)' within any CATEGORY.<br>3. Death not associated with CTCAE term – *Select* in the DEATH CATEGORY.						
NAVIGATION NOTE: Chest pain (non-cardiac and non-pleuritic) is graded as Pain – *Select* in the PAIN CATEGORY.						
NAVIGATION NOTE: CNS ischemia is graded as CNS cerebrovascular ischemia in the NEUROLOGY CATEGORY.						
Hypertension	Hypertension	Asymptomatic, transient (<24 hrs) increase by >20 mmHg (diastolic) or to >150/100 if previously WNL; intervention not indicated<br><br>Pediatric:<br>Asymptomatic, transient (<24 hrs) BP increase >ULN; intervention not indicated	Recurrent or persistent (≥24 hrs) or symptomatic increase by >20 mmHg (diastolic) or to >150/100 if previously WNL; monotherapy may be indicated<br><br>Pediatric:<br>Recurrent or persistent (≥24 hrs) BP >ULN; monotherapy may be indicated	Requiring more than one drug or more intensive therapy than previously<br><br>Pediatric:<br>Same as adult	Life-threatening consequences (e.g., hypertensive crisis)<br><br>Pediatric:<br>Same as adult	Death
REMARK: Use age and gender-appropriate normal values >95th percentile ULN for pediatric patients.						

CARDIAC GENERAL

		Grade				
**Adverse Event**	**Short Name**	**1**	**2**	**3**	**4**	**5**
Hypotension	Hypotension	Changes, intervention not indicated	Brief (<24 hrs) fluid replacement or other therapy; no physiologic consequences	Sustained (≥24 hrs) therapy, resolves without persisting physiologic consequences	Shock (e.g., acidemia; impairment of vital organ function)	Death
ALSO CONSIDER: Syncope (fainting).						
Left ventricular diastolic dysfunction	Left ventricular diastolic dysfunction	Asymptomatic diagnostic finding; intervention not indicated	Asymptomatic, intervention indicated	Symptomatic CHF responsive to intervention	Refractory CHF, poorly controlled; intervention such as ventricular assist device or heart transplant indicated	Death
Left ventricular systolic dysfunction	Left ventricular systolic dysfunction	Asymptomatic, resting ejection fraction (EF) <60 – 50%; shortening fraction (SF) <30 – 24%	Asymptomatic, resting EF <50 – 40%; SF <24 – 15%	Symptomatic CHF responsive to intervention; EF <40 – 20% SF <15%	Refractory CHF or poorly controlled; EF <20%; intervention such as ventricular assist device, ventricular reduction surgery, or heart transplant indicated	Death
NAVIGATION NOTE: Myocardial infarction is graded as Cardiac ischemia/infarction in the CARDIAC GENERAL CATEGORY.						
Myocarditis	Myocarditis	—	—	CHF responsive to intervention	Severe or refractory CHF	Death
Pericardial effusion (non-malignant)	Pericardial effusion	Asymptomatic effusion	—	Effusion with physiologic consequences	Life-threatening consequences (e.g., tamponade); emergency intervention indicated	Death
Pericarditis	Pericarditis	Asymptomatic, ECG or physical exam (rub) changes consistent with pericarditis	Symptomatic pericarditis (e.g., chest pain)	Pericarditis with physiologic consequences (e.g., pericardial constriction)	Life-threatening consequences; emergency intervention indicated	Death
NAVIGATION NOTE: Pleuritic pain is graded as Pain – *Select* in the PAIN CATEGORY.						
Pulmonary hypertension	Pulmonary hypertension	Asymptomatic without therapy	Asymptomatic, therapy indicated	Symptomatic hypertension, responsive to therapy	Symptomatic hypertension, poorly controlled	Death
Restrictive cardiomyopathy	Restrictive cardiomyopathy	Asymptomatic, therapy not indicated	Asymptomatic, therapy indicated	Symptomatic CHF responsive to intervention	Refractory CHF, poorly controlled; intervention such as ventricular assist device, or heart transplant indicated	Death

CARDIAC GENERAL

Page 3 of 3

		Grade				
**Adverse Event**	**Short Name**	**1**	**2**	**3**	**4**	**5**
Right ventricular dysfunction (cor pulmonale)	Right ventricular dysfunction	Asymptomatic without therapy	Asymptomatic, therapy indicated	Symptomatic cor pulmonale, responsive to intervention	Symptomatic cor pulmonale poorly controlled; intervention such as ventricular assist device, or heart transplant indicated	Death
Valvular heart disease	Valvular heart disease	Asymptomatic valvular thickening with or without mild valvular regurgitation or stenosis; treatment other than endocarditis prophylaxis not indicated	Asymptomatic; moderate regurgitation or stenosis by imaging	Symptomatic; severe regurgitation or stenosis; symptoms controlled with medical therapy	Life-threatening; disabling; intervention (e.g., valve replacement, valvuloplasty) indicated	Death
Cardiac General – Other (Specify, __)	Cardiac General – Other (Specify)	Mild	Moderate	Severe	Life-threatening; disabling	Death

CONSTITUTIONAL SYMPTOMS

Page 1 of 2

		Grade				
**Adverse Event**	**Short Name**	**1**	**2**	**3**	**4**	**5**
Fatigue (asthenia, lethargy, malaise)	Fatigue	Mild fatigue over baseline	Moderate or causing difficulty performing some ADL	Severe fatigue interfering with ADL	Disabling	—
Fever (in the absence of neutropenia, where neutropenia is defined as ANC <1.0 x 10^9/L)	Fever	38.0 – 39.0°C (100.4 – 102.2°F)	>39.0 – 40.0°C (102.3 – 104.0°F)	>40.0°C (>104.0°F) for ≤24 hrs	>40.0°C (>104.0°F) for >24 hrs	Death
REMARK: The temperature measurements listed are oral or tympanic. ALSO CONSIDER: Allergic reaction/hypersensitivity (including drug fever).						
NAVIGATION NOTE: Hot flashes are graded as Hot flashes/flushes in the ENDOCRINE CATEGORY.						
Hypothermia	Hypothermia	—	35 – >32°C 95 – >89.6°F	32 – >28°C 89.6 – >82.4° F	≤28 °C 82.4°F or life-threatening consequences (e.g., coma, hypotension, pulmonary edema, acidemia, ventricular fibrillation)	Death
Insomnia	Insomnia	Occasional difficulty sleeping, not interfering with function	Difficulty sleeping, interfering with function but not interfering with ADL	Frequent difficulty sleeping, interfering with ADL	Disabling	—
REMARK: If pain or other symptoms interfere with sleep, do NOT grade as insomnia. Grade primary event(s) causing insomnia.						
Obesity[2]	Obesity	—	BMI 25 – 29.9 kg/m^2	BMI 30 – 39.99 kg/m^2	BMI ≥40 kg/m^2	—
REMARK: BMI = (weight [kg]) / (height [m])^2						
Odor (patient odor)	Patient odor	Mild odor	Pronounced odor	—	—	—
Rigors/chills	Rigors/chills	Mild	Moderate, narcotics indicated	Severe or prolonged, not responsive to narcotics	—	—

DERMATOLOGY/SKIN

Adverse Event	Short Name	Grade 1	Grade 2	Grade 3	Grade 4	Grade 5
Atrophy, skin	Atrophy, skin	Detectable	Marked	—	—	—
Atrophy, subcutaneous fat	Atrophy, subcutaneous fat	Detectable	Marked	—	—	—
ALSO CONSIDER: Induration/fibrosis (skin and subcutaneous tissue).						
Bruising (in absence of Grade 3 or 4 thrombocytopenia)	Bruising	Localized or in a dependent area	Generalized	—	—	—
Burn	Burn	Minimal symptoms; intervention not indicated	Medical intervention; minimal debridement indicated	Moderate to major debridement or reconstruction indicated	Life-threatening consequences	Death
REMARK: Burn refers to all burns including radiation, chemical, etc.						
Cheilitis	Cheilitis	Asymptomatic	Symptomatic, not interfering with ADL	Symptomatic, interfering with ADL	—	—
Dry skin	Dry skin	Asymptomatic	Symptomatic, not interfering with ADL	Interfering with ADL	—	—
Flushing	Flushing	Asymptomatic	Symptomatic	—	—	—
Hair loss/alopecia (scalp or body)	Alopecia	Thinning or patchy	Complete	—	—	—
Hyperpigmentation	Hyperpigmentation	Slight or localized	Marked or generalized	—	—	—
Hypopigmentation	Hypopigmentation	Slight or localized	Marked or generalized	—	—	—
Induration/fibrosis (skin and subcutaneous tissue)	Induration	Increased density on palpation	Moderate impairment of function not interfering with ADL; marked increase in density and firmness on palpation with or without minimal retraction	Dysfunction interfering with ADL; very marked density, retraction or fixation	—	—
ALSO CONSIDER: Fibrosis-cosmesis; Fibrosis-deep connective tissue.						
Injection site reaction/ extravasation changes	Injection site reaction	Pain; itching; erythema	Pain or swelling, with inflammation or phlebitis	Ulceration or necrosis that is severe; operative intervention indicated	—	—
ALSO CONSIDER: Allergic reaction/hypersensitivity (including drug fever); Ulceration.						

DERMATOLOGY/SKIN

Adverse Event	Short Name	Grade 1	Grade 2	Grade 3	Grade 4	Grade 5
Nail changes	Nail changes	Discoloration; ridging (koilonychias); pitting	Partial or complete loss of nail(s); pain in nailbed(s)	Interfering with ADL	—	—
NAVIGATION NOTE: Petechiae is graded as Petechiae/purpura (hemorrhage/bleeding into skin or mucosa) in the HEMORRHAGE/BLEEDING CATEGORY.						
Photosensitivity	Photosensitivity	Painless erythema	Painful erythema	Erythema with desquamation	Life-threatening; disabling	Death
Pruritus/itching	Pruritus	Mild or localized	Intense or widespread	Intense or widespread and interfering with ADL	—	—
ALSO CONSIDER: Rash/desquamation.						
Rash/desquamation	Rash	Macular or papular eruption or erythema without associated symptoms	Macular or papular eruption or erythema with pruritus or other associated symptoms; localized desquamation or other lesions covering <50% of body surface area (BSA)	Severe, generalized erythroderma or macular, papular or vesicular eruption; desquamation covering ≥50% BSA	Generalized exfoliative, ulcerative, or bullous dermatitis	Death
REMARK: Rash/desquamation may be used for GVHD.						
Rash: acne/acneiform	Acne	Intervention not indicated	Intervention indicated	Associated with pain, disfigurement, ulceration, or desquamation	—	Death
Rash: dermatitis associated with radiation – *Select*: – Chemoradiation – Radiation	Dermatitis – *Select*	Faint erythema or dry desquamation	Moderate to brisk erythema; patchy moist desquamation, mostly confined to skin folds and creases; moderate edema	Moist desquamation other than skin folds and creases; bleeding induced by minor trauma or abrasion	Skin necrosis or ulceration of full thickness dermis; spontaneous bleeding from involved site	Death
Rash: erythema multiforme (e.g., Stevens-Johnson syndrome, toxic epidermal necrolysis)	Erythema multiforme	—	Scattered, but not generalized eruption	Severe (e.g., generalized rash or painful stomatitis); IV fluids, tube feedings, or TPN indicated	Life-threatening; disabling	Death
Rash: hand-foot skin reaction	Hand-foot	Minimal skin changes or dermatitis (e.g., erythema) without pain	Skin changes (e.g., peeling, blisters, bleeding, edema) or pain, not interfering with function	Ulcerative dermatitis or skin changes with pain interfering with function	—	—

DERMATOLOGY/SKIN

Page 3 of 3

		Grade				
**Adverse Event**	**Short Name**	**1**	**2**	**3**	**4**	**5**
Skin breakdown/ decubitus ulcer	Decubitus	—	Local wound care; medical intervention indicated	Operative debridement or other invasive intervention indicated (e.g., hyperbaric oxygen)	Life-threatening consequences; major invasive intervention indicated (e.g., tissue reconstruction, flap, or grafting)	Death
REMARK: Skin breakdown/decubitus ulcer is to be used for loss of skin integrity or decubitus ulcer from pressure or as the result of operative or medical intervention.						
Striae	Striae	Mild	Cosmetically significant	—	—	—
Telangiectasia	Telangiectasia	Few	Moderate number	Many and confluent	—	—
Ulceration	Ulceration	—	Superficial ulceration <2 cm size; local wound care; medical intervention indicated	Ulceration ≥2 cm size; operative debridement, primary closure or other invasive intervention indicated (e.g., hyperbaric oxygen)	Life-threatening consequences; major invasive intervention indicated (e.g., complete resection, tissue reconstruction, flap, or grafting)	Death
Urticaria (hives, welts, wheals)	Urticaria	Intervention not indicated	Intervention indicated for <24 hrs	Intervention indicated for ≥24 hrs	—	—
ALSO CONSIDER: Allergic reaction/hypersensitivity (including drug fever).						
Wound complication, non-infectious	Wound complication, non-infectious	Incisional separation of ≤25% of wound, no deeper than superficial fascia	Incisional separation >25% of wound with local care; asymptomatic hernia	Symptomatic hernia without evidence of strangulation; fascial disruption/dehiscence without evisceration; primary wound closure or revision by operative intervention indicated; hospitalization or hyperbaric oxygen indicated	Symptomatic hernia with evidence of strangulation; fascial disruption with evisceration; major reconstruction flap, grafting, resection, or amputation indicated	Death
REMARK: Wound complication, non-infectious is to be used for separation of incision, hernia, dehiscence, evisceration, or second surgery for wound revision.						
Dermatology/Skin – Other (Specify, __)	Dermatology – Other (Specify)	Mild	Moderate	Severe	Life-threatening; disabling	Death

ENDOCRINE

Page 1 of 2

		Grade				
**Adverse Event**	**Short Name**	**1**	**2**	**3**	**4**	**5**
Adrenal insufficiency	Adrenal insufficiency	Asymptomatic, intervention not indicated	Symptomatic, intervention indicated	Hospitalization	Life-threatening; disabling	Death
REMARK: Adrenal insufficiency includes any of the following signs and symptoms: abdominal pain, anorexia, constipation, diarrhea, hypotension, pigmentation of mucous membranes, pigmentation of skin, salt craving, syncope (fainting), vitiligo, vomiting, weakness, weight loss. Adrenal insufficiency must be confirmed by laboratory studies (low cortisol frequently accompanied by low aldosterone).<br>ALSO CONSIDER: Potassium, serum-high (hyperkalemia); Thyroid function, low (hypothyroidism).						
Cushingoid appearance (e.g., moon face, buffalo hump, centripetal obesity, cutaneous striae)	Cushingoid	—	Present	—	—	—
ALSO CONSIDER: Glucose, serum-high (hyperglycemia); Potassium, serum-low (hypokalemia).						
Feminization of male	Feminization of male	—	—	Present	—	—
NAVIGATION NOTE: Gynecomastia is graded in the SEXUAL/REPRODUCTIVE FUNCTION CATEGORY.						
Hot flashes/flushes[3]	Hot flashes	Mild	Moderate	Interfering with ADL	—	—
Masculinization of female	Masculinization of female	—	—	Present	—	—
Neuroendocrine: ACTH deficiency	ACTH	Asymptomatic	Symptomatic, not interfering with ADL; intervention indicated	Symptoms interfering with ADL; hospitalization indicated	Life-threatening consequences (e.g., severe hypotension)	Death
Neuroendocrine: ADH secretion abnormality (e.g., SIADH or low ADH)	ADH	Asymptomatic	Symptomatic, not interfering with ADL; intervention indicated	Symptoms interfering with ADL	Life-threatening consequences	Death
Neuroendocrine: gonadotropin secretion abnormality	Gonadotropin	Asymptomatic	Symptomatic, not interfering with ADL; intervention indicated	Symptoms interfering with ADL; osteopenia; fracture; infertility	—	—
Neuroendocrine: growth hormone secretion abnormality	Growth hormone	Asymptomatic	Symptomatic, not interfering with ADL; intervention indicated	—	—	—
Neuroendocrine: prolactin hormone secretion abnormality	Prolactin	Asymptomatic	Symptomatic, not interfering with ADL; intervention indicated	Symptoms interfering with ADL; amenorrhea; galactorrhea	—	Death

ENDOCRINE						Page 2 of 2
		Grade				
**Adverse Event**	**Short Name**	**1**	**2**	**3**	**4**	**5**
Pancreatic endocrine: glucose intolerance	Diabetes	Asymptomatic, intervention not indicated	Symptomatic; dietary modification or oral agent indicated	Symptoms interfering with ADL; insulin indicated	Life-threatening consequences (e.g., ketoacidosis, hyperosmolar non-ketotic coma)	Death
Parathyroid function, low (hypoparathyroidism)	Hypoparathyroidism	Asymptomatic, intervention not indicated	Symptomatic; intervention indicated	—	—	—
Thyroid function, high (hyperthyroidism, thyrotoxicosis)	Hyperthyroidism	Asymptomatic, intervention not indicated	Symptomatic, not interfering with ADL; thyroid suppression therapy indicated	Symptoms interfering with ADL; hospitalization indicated	Life-threatening consequences (e.g., thyroid storm)	Death
Thyroid function, low (hypothyroidism)	Hypothyroidism	Asymptomatic, intervention not indicated	Symptomatic, not interfering with ADL; thyroid replacement indicated	Symptoms interfering with ADL; hospitalization indicated	Life-threatening myxedema coma	Death
Endocrine – Other (Specify, __)	Endocrine – Other (Specify)	Mild	Moderate	Severe	Life-threatening; disabling	Death

GASTROINTESTINAL						Page 1 of 10
		Grade				
**Adverse Event**	**Short Name**	**1**	**2**	**3**	**4**	**5**
NAVIGATION NOTE: Abdominal pain or cramping is graded as Pain – *Select* in the PAIN CATEGORY.						
Anorexia	Anorexia	Loss of appetite without alteration in eating habits	Oral intake altered without significant weight loss or malnutrition; oral nutritional supplements indicated	Associated with significant weight loss or malnutrition (e.g., inadequate oral caloric and/or fluid intake); IV fluids, tube feedings or TPN indicated	Life-threatening consequences	Death
ALSO CONSIDER: Weight loss.						
Ascites (non-malignant)	Ascites	Asymptomatic	Symptomatic, medical intervention indicated	Symptomatic, invasive procedure indicated	Life-threatening consequences	Death
REMARK: Ascites (non-malignant) refers to documented non-malignant ascites or unknown etiology, but unlikely malignant, and includes chylous ascites.						
Colitis	Colitis	Asymptomatic, pathologic or radiographic findings only	Abdominal pain; mucus or blood in stool	Abdominal pain, fever, change in bowel habits with ileus; peritoneal signs	Life-threatening consequences (e.g., perforation, bleeding, ischemia, necrosis, toxic megacolon)	Death
ALSO CONSIDER: Hemorrhage, GI – *Select*.						
Constipation	Constipation	Occasional or intermittent symptoms; occasional use of stool softeners, laxatives, dietary modification, or enema	Persistent symptoms with regular use of laxatives or enemas indicated	Symptoms interfering with ADL; obstipation with manual evacuation indicated	Life-threatening consequences (e.g., obstruction, toxic megacolon)	Death
ALSO CONSIDER: Ileus, GI (functional obstruction of bowel, i.e., neuroconstipation); Obstruction, GI – *Select*.						
Dehydration	Dehydration	Increased oral fluids indicated; dry mucous membranes; diminished skin turgor	IV fluids indicated <24 hrs	IV fluids indicated ≥24 hrs	Life-threatening consequences (e.g., hemodynamic collapse)	Death
ALSO CONSIDER: Diarrhea; Hypotension; Vomiting.						
Dental: dentures or prosthesis	Dentures	Minimal discomfort, no restriction in activities	Discomfort preventing use in some activities (e.g., eating), but not others (e.g., speaking)	Unable to use dentures or prosthesis at any time	—	—

GASTROINTESTINAL						Page 2 of 10
		Grade				
**Adverse Event**	**Short Name**	**1**	**2**	**3**	**4**	**5**
Dental: periodontal disease	Periodontal	Gingival recession or gingivitis; limited bleeding on probing; mild local bone loss	Moderate gingival recession or gingivitis; multiple sites of bleeding on probing; moderate bone loss	Spontaneous bleeding; severe bone loss with or without tooth loss; osteonecrosis of maxilla or mandible	—	—
REMARK: Severe periodontal disease leading to osteonecrosis is graded as Osteonecrosis (avascular necrosis) in the MUSCULOSKELETAL CATEGORY.						
Dental: teeth	Teeth	Surface stains; dental caries; restorable, without extractions	Less than full mouth extractions; tooth fracture or crown amputation or repair indicated	Full mouth extractions indicated	—	—
Dental: teeth development	Teeth development	Hypoplasia of tooth or enamel not interfering with function	Functional impairment correctable with oral surgery	Maldevelopment with functional impairment not surgically correctable	—	—
Diarrhea	Diarrhea	Increase of <4 stools per day over baseline; mild increase in ostomy output compared to baseline	Increase of 4 – 6 stools per day over baseline; IV fluids indicated <24hrs; moderate increase in ostomy output compared to baseline; not interfering with ADL	Increase of ≥7 stools per day over baseline; incontinence; IV fluids ≥24 hrs; hospitalization; severe increase in ostomy output compared to baseline; interfering with ADL	Life-threatening consequences (e.g., hemodynamic collapse)	Death
REMARK: Diarrhea includes diarrhea of small bowel or colonic origin, and/or ostomy diarrhea.						
ALSO CONSIDER: Dehydration; Hypotension.						
Distension/bloating, abdominal	Distension	Asymptomatic	Symptomatic, but not interfering with GI function	Symptomatic, interfering with GI function	—	—
ALSO CONSIDER: Ascites (non-malignant); Ileus, GI (functional obstruction of bowel, i.e., neuroconstipation); Obstruction, GI – *Select*.						

GASTROINTESTINAL						Page 3 of 10
		Grade				
**Adverse Event**	**Short Name**	**1**	**2**	**3**	**4**	**5**
Dry mouth/salivary gland (xerostomia)	Dry mouth	Symptomatic (dry or thick saliva) without significant dietary alteration; unstimulated saliva flow >0.2 ml/min	Symptomatic and significant oral intake alteration (e.g., copious water, other lubricants, diet limited to purees and/or soft, moist foods); unstimulated saliva 0.1 to 0.2 ml/min	Symptoms leading to inability to adequately aliment orally; IV fluids, tube feedings, or TPN indicated; unstimulated saliva <0.1 ml/min	—	—
REMARK: Dry mouth/salivary gland (xerostomia) includes descriptions of grade using both subjective and objective assessment parameters. Record this event consistently throughout a patient's participation on study. If salivary flow measurements are used for initial assessment, subsequent assessments must use salivary flow.						
ALSO CONSIDER: Salivary gland changes/saliva.						
Dysphagia (difficulty swallowing)	Dysphagia	Symptomatic, able to eat regular diet	Symptomatic and altered eating/swallowing (e.g., altered dietary habits, oral supplements); IV fluids indicated <24 hrs	Symptomatic and severely altered eating/swallowing (e.g., inadequate oral caloric or fluid intake); IV fluids, tube feedings, or TPN indicated ≥24 hrs	Life-threatening consequences (e.g., obstruction, perforation)	Death
REMARK: Dysphagia (difficulty swallowing) is to be used for swallowing difficulty from oral, pharyngeal, esophageal, or neurologic origin. Dysphagia requiring dilation is graded as Stricture/stenosis (including anastomotic), GI – *Select*.						
ALSO CONSIDER: Dehydration; Esophagitis.						
Enteritis (inflammation of the small bowel)	Enteritis	Asymptomatic, pathologic or radiographic findings only	Abdominal pain; mucus or blood in stool	Abdominal pain, fever, change in bowel habits with ileus; peritoneal signs	Life-threatening consequences (e.g., perforation, bleeding, ischemia, necrosis)	Death
ALSO CONSIDER: Hemorrhage, GI – *Select*; Typhlitis (cecal inflammation).						
Esophagitis	Esophagitis	Asymptomatic pathologic, radiographic, or endoscopic findings only	Symptomatic; altered eating/swallowing (e.g., altered dietary habits, oral supplements); IV fluids indicated <24 hrs	Symptomatic and severely altered eating/swallowing (e.g., inadequate oral caloric or fluid intake); IV fluids, tube feedings, or TPN indicated ≥24 hrs	Life-threatening consequences	Death
REMARK: Esophagitis includes reflux esophagitis.						
ALSO CONSIDER: Dysphagia (difficulty swallowing).						

		Grade				
Adverse Event	Short Name	1	2	3	4	5
Fistula, GI<br>– *Select*:<br>– Abdomen NOS<br>– Anus<br>– Biliary tree<br>– Colon/cecum/appendix<br>– Duodenum<br>– Esophagus<br>– Gallbladder<br>– Ileum<br>– Jejunum<br>– Oral cavity<br>– Pancreas<br>– Pharynx<br>– Rectum<br>– Salivary gland<br>– Small bowel NOS<br>– Stomach	Fistula, GI – *Select*	Asymptomatic, radiographic findings only	Symptomatic; altered GI function (e.g., altered dietary habits, diarrhea, or GI fluid loss); IV fluids indicated <24 hrs	Symptomatic and severely altered GI function (e.g., altered dietary habits, diarrhea, or GI fluid loss); IV fluids, tube feedings, or TPN indicated ≥24 hrs	Life-threatening consequences	Death
REMARK: A fistula is defined as an abnormal communication between two body cavities, potential spaces, and/or the skin. The site indicated for a fistula should be the site from which the abnormal process is believed to have originated. For example, a tracheo-esophageal fistula arising in the context of a resected or irradiated esophageal cancer is graded as Fistula, GI – esophagus.						
Flatulence	Flatulence	Mild	Moderate	—	—	—
Gastritis (including bile reflux gastritis)	Gastritis	Asymptomatic radiographic or endoscopic findings only	Symptomatic; altered gastric function (e.g., inadequate oral caloric or fluid intake); IV fluids indicated <24 hrs	Symptomatic and severely altered gastric function (e.g., inadequate oral caloric or fluid intake); IV fluids, tube feedings, or TPN indicated ≥24 hrs	Life-threatening consequences; operative intervention requiring complete organ resection (e.g., gastrectomy)	Death
ALSO CONSIDER: Hemorrhage, GI – *Select*; Ulcer, GI – *Select*.						
NAVIGATION NOTE: Head and neck soft tissue necrosis is graded as Soft tissue necrosis – *Select* in the MUSCULOSKELETAL/SOFT TISSUE CATEGORY.						
Heartburn/dyspepsia	Heartburn	Mild	Moderate	Severe	—	—
Hemorrhoids	Hemorrhoids	Asymptomatic	Symptomatic; banding or medical intervention indicated	Interfering with ADL; interventional radiology, endoscopic, or operative intervention indicated	Life-threatening consequences	Death

		Grade				
Adverse Event	Short Name	1	2	3	4	5
Mucositis/stomatitis (clinical exam)<br>– *Select*:<br>– Anus<br>– Esophagus<br>– Large bowel<br>– Larynx<br>– Oral cavity<br>– Pharynx<br>– Rectum<br>– Small bowel<br>– Stomach<br>– Trachea	Mucositis (clinical exam) – *Select*	Erythema of the mucosa	Patchy ulcerations or pseudomembranes	Confluent ulcerations or pseudomembranes; bleeding with minor trauma	Tissue necrosis; significant spontaneous bleeding; life-threatening consequences	Death
REMARK: Mucositis/stomatitis (functional/symptomatic) may be used for mucositis of the upper aero-digestive tract caused by radiation, agents, or GVHD.						
Mucositis/stomatitis (functional/symptomatic)<br>– *Select*:<br>– Anus<br>– Esophagus<br>– Large bowel<br>– Larynx<br>– Oral cavity<br>– Pharynx<br>– Rectum<br>– Small bowel<br>– Stomach<br>– Trachea	Mucositis (functional/ symptomatic) – *Select*	Upper aerodigestive tract sites: Minimal symptoms, normal diet; minimal respiratory symptoms but not interfering with function<br><br>Lower GI sites: Minimal discomfort, intervention not indicated	Upper aerodigestive tract sites: Symptomatic but can eat and swallow modified diet; respiratory symptoms interfering with function but not interfering with ADL<br><br>Lower GI sites: Symptomatic, medical intervention indicated but not interfering with ADL	Upper aerodigestive tract sites: Symptomatic and unable to adequately aliment or hydrate orally; respiratory symptoms interfering with ADL<br><br>Lower GI sites: Stool incontinence or other symptoms interfering with ADL	Symptoms associated with life-threatening consequences	Death
Nausea	Nausea	Loss of appetite without alteration in eating habits	Oral intake decreased without significant weight loss, dehydration or malnutrition; IV fluids indicated <24 hrs	Inadequate oral caloric or fluid intake; IV fluids, tube feedings, or TPN indicated ≥24 hrs	Life-threatening consequences	Death
ALSO CONSIDER: Anorexia; Vomiting.						

GASTROINTESTINAL

Page 10 of 10

Adverse Event	Short Name	Grade 1	Grade 2	Grade 3	Grade 4	Grade 5
Ulcer, GI<br>– *Select*:<br>– Anus<br>– Cecum<br>– Colon<br>– Duodenum<br>– Esophagus<br>– Ileum<br>– Jejunum<br>– Rectum<br>– Small bowel NOS<br>– Stoma<br>– Stomach	Ulcer, GI – *Select*	Asymptomatic, radiographic or endoscopic findings only	Symptomatic; altered GI function (e.g., altered dietary habits, oral supplements); IV fluids indicated <24 hrs	Symptomatic and severely altered GI function (e.g., inadequate oral caloric or fluid intake); IV fluids, tube feedings, or TPN indicated ≥24 hrs	Life-threatening consequences	Death
ALSO CONSIDER: Hemorrhage, GI – *Select*.						
Vomiting	Vomiting	1 episode in 24 hrs	2 – 5 episodes in 24 hrs; IV fluids indicated <24 hrs	≥6 episodes in 24 hrs; IV fluids, or TPN indicated ≥24 hrs	Life-threatening consequences	Death
ALSO CONSIDER: Dehydration.						
Gastrointestinal – Other (Specify, __)	GI – Other (Specify)	Mild	Moderate	Severe	Life-threatening; disabling	Death

HEMORRHAGE/BLEEDING

Page 1 of 4

Adverse Event	Short Name	Grade 1	Grade 2	Grade 3	Grade 4	Grade 5
Hematoma	Hematoma	Minimal symptoms, invasive intervention not indicated	Minimally invasive evacuation or aspiration indicated	Transfusion, interventional radiology, or operative intervention indicated	Life-threatening consequences; major urgent intervention indicated	Death
REMARK: Hematoma refers to extravasation at wound or operative site or secondary to other intervention. Transfusion implies pRBC.						
ALSO CONSIDER: Fibrinogen; INR (International Normalized Ratio of prothrombin time); Platelets; PTT (Partial Thromboplastin Time).						
Hemorrhage/bleeding associated with surgery, intra-operative or postoperative	Hemorrhage with surgery	—	—	Requiring transfusion of 2 units non-autologous (10 cc/kg for pediatrics) pRBCs beyond protocol specification; postoperative interventional radiology, endoscopic, or operative intervention indicated	Life-threatening consequences	Death
REMARK: Postoperative period is defined as ≤72 hours after surgery. Verify protocol-specific acceptable guidelines regarding pRBC transfusion.						
ALSO CONSIDER: Fibrinogen; INR (International Normalized Ratio of prothrombin time); Platelets; PTT (Partial Thromboplastin Time).						
Hemorrhage, CNS	CNS hemorrhage	Asymptomatic, radiographic findings only	Medical intervention indicated	Ventriculostomy, ICP monitoring, intraventricular thrombolysis, or operative intervention indicated	Life-threatening consequences; neurologic deficit or disability	Death
ALSO CONSIDER: Fibrinogen; INR (International Normalized Ratio of prothrombin time); Platelets; PTT (Partial Thromboplastin Time).						

HEMORRHAGE/BLEEDING

Page 2 of 4

Adverse Event	Short Name	Grade 1	Grade 2	Grade 3	Grade 4	Grade 5
Hemorrhage, GI<br>– *Select*:<br>– Abdomen NOS<br>– Anus<br>– Biliary tree<br>– Cecum/appendix<br>– Colon<br>– Duodenum<br>– Esophagus<br>– Ileum<br>– Jejunum<br>– Liver<br>– Lower GI NOS<br>– Oral cavity<br>– Pancreas<br>– Peritoneal cavity<br>– Rectum<br>– Stoma<br>– Stomach<br>– Upper GI NOS<br>– Varices (esophageal)<br>– Varices (rectal)	Hemorrhage, GI – *Select*	Mild, intervention (other than iron supplements) not indicated	Symptomatic and medical intervention or minor cauterization indicated	Transfusion, interventional radiology, endoscopic, or operative intervention indicated; radiation therapy (i.e., hemostasis of bleeding site)	Life-threatening consequences; major urgent intervention indicated	Death
REMARK: Transfusion implies pRBC.						
ALSO CONSIDER: Fibrinogen; INR (International Normalized Ratio of prothrombin time); Platelets; PTT (Partial Thromboplastin Time).						

HEMORRHAGE/BLEEDING						Page 3 of 4
		Grade				
**Adverse Event**	**Short Name**	**1**	**2**	**3**	**4**	**5**
Hemorrhage, GU<br>– *Select*:<br>– Bladder<br>– Fallopian tube<br>– Kidney<br>– Ovary<br>– Prostate<br>– Retroperitoneum<br>– Spermatic cord<br>– Stoma<br>– Testes<br>– Ureter<br>– Urethra<br>– Urinary NOS<br>– Uterus<br>– Vagina<br>– Vas deferens	Hemorrhage, GU – *Select*	Minimal or microscopic bleeding; intervention not indicated	Gross bleeding, medical intervention, or urinary tract irrigation indicated	Transfusion, interventional radiology, endoscopic, or operative intervention indicated; radiation therapy (i.e., hemostasis of bleeding site)	Life-threatening consequences; major urgent intervention indicated	Death
REMARK: Transfusion implies pRBC.						
ALSO CONSIDER: Fibrinogen; INR (International Normalized Ratio of prothrombin time); Platelets; PTT (Partial Thromboplastin Time).						
Hemorrhage, pulmonary/ upper respiratory<br>– *Select*:<br>– Bronchopulmonary NOS<br>– Bronchus<br>– Larynx<br>– Lung<br>– Mediastinum<br>– Nose<br>– Pharynx<br>– Pleura<br>– Respiratory tract NOS<br>– Stoma<br>– Trachea	Hemorrhage pulmonary – *Select*	Mild, intervention not indicated	Symptomatic and medical intervention indicated	Transfusion, interventional radiology, endoscopic, or operative intervention indicated; radiation therapy (i.e., hemostasis of bleeding site)	Life-threatening consequences; major urgent intervention indicated	Death
REMARK: Transfusion implies pRBC.						
ALSO CONSIDER: Fibrinogen; INR (International Normalized Ratio of prothrombin time); Platelets; PTT (Partial Thromboplastin Time).						
Petechiae/purpura (hemorrhage/bleeding into skin or mucosa)	Petechiae	Few petechiae	Moderate petechiae; purpura	Generalized petechiae or purpura	—	—
ALSO CONSIDER: Fibrinogen; INR (International Normalized Ratio of prothrombin time); Platelets; PTT (Partial Thromboplastin Time).						

HEMORRHAGE/BLEEDING						Page 4 of 4
		Grade				
**Adverse Event**	**Short Name**	**1**	**2**	**3**	**4**	**5**
NAVIGATION NOTE: Vitreous hemorrhage is graded in the OCULAR/VISUAL CATEGORY.						
Hemorrhage/Bleeding – Other (Specify, __)	Hemorrhage – Other (Specify)	Mild without transfusion	—	Transfusion indicated	Catastrophic bleeding, requiring major non-elective intervention	Death

INFECTION						Page 1 of 3
		Grade				
**Adverse Event**	**Short Name**	**1**	**2**	**3**	**4**	**5**
Colitis, infectious (e.g., Clostridium difficile)	Colitis, infectious	Asymptomatic, pathologic or radiographic findings only	Abdominal pain with mucus and/or blood in stool	IV antibiotics or TPN indicated	Life-threatening consequences (e.g., perforation, bleeding, ischemia, necrosis or toxic megacolon); operative resection or diversion indicated	Death
ALSO CONSIDER: Hemorrhage, GI – *Select*; Typhlitis (cecal inflammation).						
Febrile neutropenia (fever of unknown origin without clinically or microbiologically documented infection) (ANC <1.0 x 10^9/L, fever ≥38.5°C)	Febrile neutropenia	—	—	Present	Life-threatening consequences (e.g., septic shock, hypotension, acidosis, necrosis)	Death
ALSO CONSIDER: Neutrophils/granulocytes (ANC/AGC).						
Infection (documented clinically or microbiologically) with Grade 3 or 4 neutrophils (ANC <1.0 x 10^9/L) – *Select*<br>'*Select*' AEs appear at the end of the CATEGORY.	Infection (documented clinically) with Grade 3 or 4 ANC – *Select*	—	Localized, local intervention indicated	IV antibiotic, antifungal, or antiviral intervention indicated; interventional radiology or operative intervention indicated	Life-threatening consequences (e.g., septic shock, hypotension, acidosis, necrosis)	Death
REMARK: Fever with Grade 3 or 4 neutrophils in the absence of documented infection is graded as Febrile neutropenia (fever of unknown origin without clinically or microbiologically documented infection).<br>ALSO CONSIDER: Neutrophils/granulocytes (ANC/AGC).						
Infection with normal ANC or Grade 1 or 2 neutrophils – *Select*<br>'*Select*' AEs appear at the end of the CATEGORY.	Infection with normal ANC – *Select*	—	Localized, local intervention indicated	IV antibiotic, antifungal, or antiviral intervention indicated; interventional radiology or operative intervention indicated	Life-threatening consequences (e.g., septic shock, hypotension, acidosis, necrosis)	Death

INFECTION						Page 2 of 3
		Grade				
**Adverse Event**	**Short Name**	**1**	**2**	**3**	**4**	**5**
Infection with unknown ANC – *Select*<br>'*Select*' AEs appear at the end of the CATEGORY.	Infection with unknown ANC – *Select*	—	Localized, local intervention indicated	IV antibiotic, antifungal, or antiviral intervention indicated; interventional radiology or operative intervention indicated	Life-threatening consequences (e.g., septic shock, hypotension, acidosis, necrosis)	Death
REMARK: Infection with unknown ANC – *Select* is to be used in the rare case when ANC is unknown.						
Opportunistic infection associated with ≥Grade 2 Lymphopenia	Opportunistic infection	—	Localized, local intervention indicated	IV antibiotic, antifungal, or antiviral intervention indicated; interventional radiology or operative intervention indicated	Life-threatening consequences (e.g., septic shock, hypotension, acidosis, necrosis)	Death
ALSO CONSIDER: Lymphopenia.						
Viral hepatitis	Viral hepatitis	Present; transaminases and liver function normal	Transaminases abnormal, liver function normal	Symptomatic liver dysfunction; fibrosis by biopsy; compensated cirrhosis	Decompensated liver function (e.g., ascites, coagulopathy, encephalopathy, coma)	Death
REMARK: Non-viral hepatitis is graded as Infection – *Select*.<br>ALSO CONSIDER: Albumin, serum-low (hypoalbuminemia); ALT, SGPT (serum glutamic pyruvic transaminase); AST, SGOT (serum glutamic oxaloacetic transaminase); Bilirubin (hyperbilirubinemia); Encephalopathy.						
Infection – Other (Specify, __)	Infection – Other (Specify)	Mild	Moderate	Severe	Life-threatening; disabling	Death

INFECTION – *SELECT*

AUDITORY/EAR
- External ear (otitis externa)
- Middle ear (otitis media)

CARDIOVASCULAR
- Artery
- Heart (endocarditis)
- Spleen
- Vein

DERMATOLOGY/SKIN
- Lip/perioral
- Peristomal
- Skin (cellulitis)
- Ungual (nails)

GASTROINTESTINAL
- Abdomen NOS
- Anal/perianal
- Appendix
- Cecum
- Colon
- Dental-tooth
- Duodenum
- Esophagus
- Ileum
- Jejunum
- Oral cavity-gums (gingivitis)
- Peritoneal cavity
- Rectum
- Salivary gland
- Small bowel NOS
- Stomach

GENERAL
- Blood
- Catheter-related
- Foreign body (e.g., graft, implant, prosthesis, stent)
- Wound

HEPATOBILIARY/PANCREAS
- Biliary tree
- Gallbladder (cholecystitis)
- Liver
- Pancreas

LYMPHATIC
- Lymphatic

MUSCULOSKELETAL
- Bone (osteomyelitis)
- Joint
- Muscle (infection myositis)
- Soft tissue NOS

NEUROLOGY
- Brain (encephalitis, infectious)
- Brain + Spinal cord (encephalomyelitis)
- Meninges (meningitis)
- Nerve-cranial
- Nerve-peripheral
- Spinal cord (myelitis)

OCULAR
- Conjunctiva
- Cornea
- Eye NOS
- Lens

PULMONARY/UPPER RESPIRATORY
- Bronchus
- Larynx
- Lung (pneumonia)
- Mediastinum NOS
- Mucosa
- Neck NOS
- Nose
- Paranasal
- Pharynx
- Pleura (empyema)
- Sinus
- Trachea
- Upper aerodigestive NOS
- Upper airway NOS

RENAL/GENITOURINARY
- Bladder (urinary)
- Kidney
- Prostate
- Ureter
- Urethra
- Urinary tract NOS

SEXUAL/REPRODUCTIVE FUNCTION
- Cervix
- Fallopian tube
- Pelvis NOS
- Penis
- Scrotum
- Uterus
- Vagina
- Vulva

METABOLIC/LABORATORY

		Grade				
**Adverse Event**	**Short Name**	**1**	**2**	**3**	**4**	**5**
Acidosis (metabolic or respiratory)	Acidosis	pH <normal, but ≥7.3	—	pH <7.3	pH <7.3 with life-threatening consequences	Death
Albumin, serum-low (hypoalbuminemia)	Hypoalbuminemia	<LLN – 3 g/dL<br><LLN – 30 g/L	<3 – 2 g/dL<br><30 – 20 g/L	<2 g/dL<br><20 g/L	—	Death
Alkaline phosphatase	Alkaline phosphatase	>ULN – 2.5 x ULN	>2.5 – 5.0 x ULN	>5.0 – 20.0 x ULN	>20.0 x ULN	—
Alkalosis (metabolic or respiratory)	Alkalosis	pH >normal, but ≤7.5	—	pH >7.5	pH >7.5 with life-threatening consequences	Death
ALT, SGPT (serum glutamic pyruvic transaminase)	ALT	>ULN – 2.5 x ULN	>2.5 – 5.0 x ULN	>5.0 – 20.0 x ULN	>20.0 x ULN	—
Amylase	Amylase	>ULN – 1.5 x ULN	>1.5 – 2.0 x ULN	>2.0 – 5.0 x ULN	>5.0 x ULN	—
AST, SGOT (serum glutamic oxaloacetic transaminase)	AST	>ULN – 2.5 x ULN	>2.5 – 5.0 x ULN	>5.0 – 20.0 x ULN	>20.0 x ULN	—
Bicarbonate, serum-low	Bicarbonate, serum-low	<LLN – 16 mmol/L	<16 – 11 mmol/L	<11 – 8 mmol/L	<8 mmol/L	Death
Bilirubin (hyperbilirubinemia)	Bilirubin	>ULN – 1.5 x ULN	>1.5 – 3.0 x ULN	>3.0 – 10.0 x ULN	>10.0 x ULN	—
REMARK: Jaundice is not an AE, but may be a manifestation of liver dysfunction/failure or elevated bilirubin. If jaundice is associated with elevated bilirubin, grade bilirubin.						
Calcium, serum-low (hypocalcemia)	Hypocalcemia	<LLN – 8.0 mg/dL<br><LLN – 2.0 mmol/L<br><br>Ionized calcium:<br><LLN – 1.0 mmol/L	<8.0 – 7.0 mg/dL<br><2.0 – 1.75 mmol/L<br><br>Ionized calcium:<br><1.0 – 0.9 mmol/L	<7.0 – 6.0 mg/dL<br><1.75 – 1.5 mmol/L<br><br>Ionized calcium:<br><0.9 – 0.8 mmol/L	<6.0 mg/dL<br><1.5 mmol/L<br><br>Ionized calcium:<br><0.8 mmol/L	Death
REMARK: Calcium can be falsely low if hypoalbuminemia is present. Serum albumin is <4.0 g/dL, hypocalcemia is reported after the following corrective calculation has been performed: Corrected Calcium (mg/dL) = Total Calcium (mg/dL) – 0.8 [Albumin (g/dL) – 4] [4]. Alternatively, direct measurement of ionized calcium is the definitive method to diagnose metabolically relevant alterations in serum calcium.						

METABOLIC/LABORATORY

Adverse Event	Short Name	Grade 1	Grade 2	Grade 3	Grade 4	Grade 5
Calcium, serum-high (hypercalcemia)	Hypercalcemia	>ULN – 11.5 mg/dL<br>>ULN – 2.9 mmol/L<br><br>Ionized calcium:<br>>ULN – 1.5 mmol/L	>11.5 – 12.5 mg/dL<br>>2.9 – 3.1 mmol/L<br><br>Ionized calcium:<br>>1.5 – 1.6 mmol/L	>12.5 – 13.5 mg/dL<br>>3.1 – 3.4 mmol/L<br><br>Ionized calcium:<br>>1.6 – 1.8 mmol/L	>13.5 mg/dL<br>>3.4 mmol/L<br><br>Ionized calcium:<br>>1.8 mmol/L	Death
Cholesterol, serum-high (hypercholesteremia)	Cholesterol	>ULN – 300 mg/dL<br>>ULN – 7.75 mmol/L	>300 – 400 mg/dL<br>>7.75 – 10.34 mmol/L	>400 – 500 mg/dL<br>>10.34 – 12.92 mmol/L	>500 mg/dL<br>>12.92 mmol/L	Death
CPK (creatine phosphokinase)	CPK	>ULN – 2.5 x ULN	>2.5 x ULN – 5 x ULN	>5 x ULN – 10 x ULN	>10 x ULN	Death
Creatinine	Creatinine	>ULN – 1.5 x ULN	>1.5 – 3.0 x ULN	>3.0 – 6.0 x ULN	>6.0 x ULN	Death
REMARK: Adjust to age-appropriate levels for pediatric patients.<br>ALSO CONSIDER: Glomerular filtration rate.						
GGT (γ-Glutamyl transpeptidase)	GGT	>ULN – 2.5 x ULN	>2.5 – 5.0 x ULN	>5.0 – 20.0 x ULN	>20.0 x ULN	—
Glomerular filtration rate	GFR	<75 – 50% LLN	<50 – 25% LLN	<25% LLN, chronic dialysis not indicated	Chronic dialysis or renal transplant indicated	Death
ALSO CONSIDER: Creatinine.						
Glucose, serum-high (hyperglycemia)	Hyperglycemia	>ULN – 160 mg/dL<br>>ULN – 8.9 mmol/L	>160 – 250 mg/dL<br>>8.9 – 13.9 mmol/L	>250 – 500 mg/dL<br>>13.9 – 27.8 mmol/L	>500 mg/dL<br>>27.8 mmol/L or acidosis	Death
REMARK: Hyperglycemia, in general, is defined as fasting unless otherwise specified in protocol.						
Glucose, serum-low (hypoglycemia)	Hypoglycemia	<LLN – 55 mg/dL<br><LLN – 3.0 mmol/L	<55 – 40 mg/dL<br><3.0 – 2.2 mmol/L	<40 – 30 mg/dL<br><2.2 – 1.7 mmol/L	<30 mg/dL<br><1.7 mmol/L	Death
Hemoglobinuria	Hemoglobinuria	Present	—	—	—	Death
Lipase	Lipase	>ULN – 1.5 x ULN	>1.5 – 2.0 x ULN	>2.0 – 5.0 x ULN	>5.0 x ULN	—
Magnesium, serum-high (hypermagnesemia)	Hypermagnesemia	>ULN – 3.0 mg/dL<br>>ULN – 1.23 mmol/L	—	>3.0 – 8.0 mg/dL<br>>1.23 – 3.30 mmol/L	>8.0 mg/dL<br>>3.30 mmol/L	Death
Magnesium, serum-low (hypomagnesemia)	Hypomagnesemia	<LLN – 1.2 mg/dL<br><LLN – 0.5 mmol/L	<1.2 – 0.9 mg/dL<br><0.5 – 0.4 mmol/L	<0.9 – 0.7 mg/dL<br><0.4 – 0.3 mmol/L	<0.7 mg/dL<br><0.3 mmol/L	Death
Phosphate, serum-low (hypophosphatemia)	Hypophosphatemia	<LLN – 2.5 mg/dL<br><LLN – 0.8 mmol/L	<2.5 – 2.0 mg/dL<br><0.8 – 0.6 mmol/L	<2.0 – 1.0 mg/dL<br><0.6 – 0.3 mmol/L	<1.0 mg/dL<br><0.3 mmol/L	Death
Potassium, serum-high (hyperkalemia)	Hyperkalemia	>ULN – 5.5 mmol/L	>5.5 – 6.0 mmol/L	>6.0 – 7.0 mmol/L	>7.0 mmol/L	Death

METABOLIC/LABORATORY

Adverse Event	Short Name	Grade 1	Grade 2	Grade 3	Grade 4	Grade 5
Potassium, serum-low (hypokalemia)	Hypokalemia	<LLN – 3.0 mmol/L	—	<3.0 – 2.5 mmol/L	<2.5 mmol/L	Death
Proteinuria	Proteinuria	1+ or<br>0.15 – 1.0 g/24 hrs	2+ to 3+ or<br>>1.0 – 3.5 g/24 hrs	4+ or<br>>3.5 g/24 hrs	Nephrotic syndrome	Death
Sodium, serum-high (hypernatremia)	Hypernatremia	>ULN – 150 mmol/L	>150 – 155 mmol/L	>155 – 160 mmol/L	>160 mmol/L	Death
Sodium, serum-low (hyponatremia)	Hyponatremia	<LLN – 130 mmol/L	—	<130 – 120 mmol/L	<120 mmol/L	Death
Triglyceride, serum-high (hypertriglyceridemia)	Hypertriglyceridemia	>ULN – 2.5 x ULN	>2.5 – 5.0 x ULN	>5.0 – 10 x ULN	>10 x ULN	Death
Uric acid, serum-high (hyperuricemia)	Hyperuricemia	>ULN – 10 mg/dL<br>≤0.59 mmol/L without physiologic consequences	—	>ULN – 10 mg/dL<br>≤0.59 mmol/L with physiologic consequences	>10 mg/dL<br>>0.59 mmol/L	Death
ALSO CONSIDER: Creatinine; Potassium, serum-high (hyperkalemia); Renal failure; Tumor lysis syndrome.						
Metabolic/Laboratory – Other (Specify, __)	Metabolic/Lab – Other (Specify)	Mild	Moderate	Severe	Life-threatening; disabling	Death

VASCULAR						Page 2 of 2
		Grade				
**Adverse Event**	**Short Name**	**1**	**2**	**3**	**4**	**5**
Vessel injury-vein – *Select*: – Extremity-lower – Extremity-upper – IVC – Jugular – Other NOS – SVC – Viscera	Vein injury – *Select*	Asymptomatic diagnostic finding; intervention not indicated	Symptomatic (e.g., claudication); not interfering with ADL; repair or revision not indicated	Symptomatic interfering with ADL; repair or revision indicated	Life-threatening; disabling; evidence of end organ damage	Death
NAVIGATION NOTE: Vessel injury to a vein intra-operatively is graded as Intra-operative injury – *Select Organ or Structure* in the SURGERY/INTRA-OPERATIVE INJURY CATEGORY.						
Visceral arterial ischemia (non-myocardial)	Visceral arterial ischemia	—	Brief (<24 hrs) episode of ischemia managed medically and without permanent deficit	Prolonged (≥24 hrs) or recurring symptoms and/or invasive intervention indicated	Life-threatening; disabling; evidence of end organ damage	Death
ALSO CONSIDER: CNS cerebrovascular ischemia.						
Vascular – Other (Specify, __)	Vascular – Other (Specify)	Mild	Moderate	Severe	Life-threatening; disabling	Death

Statistical Analysis Plan

ADIUVO Study

TRIAL FULL TITLE	EFFICACY OF ADJUVANT MITOTANE TREATMENT IN PROLONGING RECURRENCE-FREE SURVIVAL IN PATIENTS WITH ADRENOCORTICAL CARCINOMA AT LOW-INTERMEDIATE RISK OF RECURRENCE. (ADIUVO Study)
ClinicalTrials.gov Identifier	NCT00777244
SAP VERSION	1.1
SAP VERSION DATE	10.1.2019
PROTOCOL VERSION	2
PROTOCOL VERSION DATE	15.7.2009
TRIAL STATISTICIANS	Until 31.12.2010 Dr. Gianni Ciccone<br>Since 1.1.2011 Dr Paola Berchialla, Dr Paolo Bruzzi
TRIAL CHIEF INVESTIGATOR	Dr. Massimo Terzolo
SAP AUTHOR	Dr. Paola Berchialla

SAP Signatures

Chief Investigator

Name: Massimo Terzolo

Signature:

Date: 10.1.2019

Senior Statisticians

Name: Paola Berchialla Paolo Bruzzi

Signature:

Date: 10.1.2019

1 Abbreviations

ACC	Adrenocortical Cancer
AE	Adverse Event
ALT	Alanine aminotransferase
AST	Aspartate aminotransferase
COACT	Collaborative group for Adrenocortical Carcinoma Therapy
CT	Computed Tomography
ECG	Electrocardiogram
ECOG	Eastern Cooperative Oncology Group
ENSAT	European Network for the Study of Adrenal Tumors
FIRMACT	First International Randomized trial in locally advanced and Metastatic Adrenocortical Carcinoma Treatment
GOT	Glutamic oxaloacetic transaminase
GPT	Glutamic pyruvic transaminase
HR	Hazard Ratio
IQR	Interquartile range
ITT	Intention to treat
MDRD	Modification of Diet in Renal Disease
MRI	Magnetic Resonance Imagine
OS	Overall Survival
RFS	Recurrence-free Survival
TTR	Time to recurrence

2 Amendment history

Date	Summary of changes	Reason for amendment
January 2019	Addition of summary of changes to the protocol.<br><br>Assessment of histopathological variants removed from secondary endpoints.	Updated based on Steering Committee decision to stop patient enrolment.<br><br>Due to the reduced number of patients included.

3 Introduction

3.1 Rationale

ACC is a very rare disease with a high risk of relapse after radical surgery. The efficacy of adjuvant mitotane treatment is supported by a retrospective multicenter international study showing that postoperative mitotane treatment was associated with a significant reduction of the risk of relapse and death. However, these promising results need confirmation in a randomized prospective study. Caution should be adopted particularly in patients with low/intermediate risk of disease relapse, in whom the benefit of therapy should be weighed against the side effects. Whereas, based on the available evidence, adjuvant treatment is justified in patients at high risk of relapse, a randomized prospective study is needed to assess whether such a treatment is efficacious in low-risk patients.

The aim of the trial is to test whether adjuvant mitotane treatment following complete tumor removal may prolong RFS in patients with ACC at low-intermediate risk of recurrence. The trial was implemented on a multicentric and international basis following the fruitful experience of the FIRMACT study, an investigator-driven initiative that has been endorsed on a European scale by an international steering committee of which we are a part. This trial took advantage of the existence of a number of collaborative networks that have been established in recent years in Europe (COACT, ENSAT).

3.2 Purpose of the analyses

The study analysis will be based on the null hypothesis that there is no difference in RFS between patients who were treated with adjuvant mitotane and those left on active surveillance only. The alternative hypotheses are that there are differences between the two groups.

4 Study Objectives and Endpoints

4.1 Study Objectives

The objective of the study is the evaluation of efficacy and safety of adjuvant mitotane treatment or surveillance only on RFS in patients with ACC at low-intermediate risk of recurrence after complete tumor resection.

4.2 Primary Endpoint

Recurrence Free Survival (RFS), to be compared between patients with ACC assigned to adjuvant mitotane and those assigned to active surveillance only following complete tumor removal.

RFS is defined as the time between the date of randomization until documentation of either of the following events (whichever occurs first):

- local or distant recurrence of ACC
- death from any cause

Censoring at the date of the last assessment for the patients who had not experienced an event.

4.3 Secondary Endpoints

- Overall Survival (OS), defined as the time interval between the date of randomization and the date of death from any cause.
- Time To Recurrence (TTR), defined as the time interval between the date of randomization until documentation of local or distant recurrence of ACC, or death from ACC (whichever occurs first).
- Quality of life, measured by the standardized EORTC-QLQ-C30 questionnaire. The EORTC-QLQ-C30 is a standardized questionnaire developed to assess the quality of life of cancer patients. It incorporates nine multi-item scales: five functional scales (physical, role, cognitive, emotional, and social); three symptom scales (fatigue, pain, nausea and vomiting); and a global health and quality-of-life scale. Two measures for the change are dealt with, the change in global quality of life at the time of first evaluation and the average change in global quality of life within the first two years or

up to recurrence (whichever occurs first) both in reference to the baseline value.

- Safety analysis. Safety measures that will be used in the study include physical examination and clinical laboratory tests (hematology, blood chemistries, and creatinine clearance).
- Incidence of second primary cancers.
- Assessment of the impact of plasma mitotane concentrations and time needed to reach the therapeutic concentrations on the efficacy of treatment. Therapeutic concentrations are defined as plasma mitotane levels $\geq$ 14 mg/L, and we will assess RFS, TTR, and OS in patients who achieve or not plasma mitotane concentrations $\geq$ 14 mg/L.
- Assessment of the efficacy of mitotane administration in predefined subgroups of patients stratified according to:
    - type of hormone secretion (cortisol secreting tumors *vs* purely sex-hormone secreting tumors *vs* non secreting tumors),
    - stage of disease (according to the 2008 ENSAT classification).

5 Study Methods

5.1 General Study Design and Plan

The study is designed as a prospective, randomized, controlled, open-label, multi-center, international, phase III trial for patients with ACC after radical resection fulfilling the inclusion criteria and in whom no exclusion criteria are met. In a parallel group design, patients are randomized 1:1 to receive post-operative adjuvant therapy with mitotane or observational follow-up only.

Figure 1. Scheme of the ADIUVO trial.

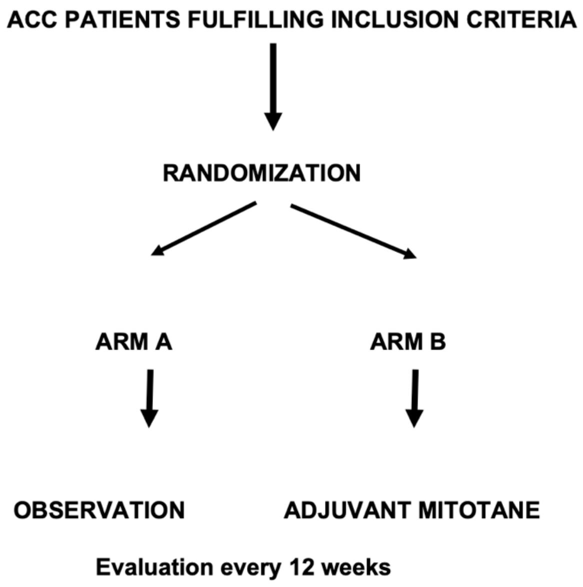

5.2 Study procedures

The following table provides the frequency and timing of the different study investigations:

	Baseline	Every month[1]	Every 3 months[2]
Physical Examination	x	x	x
Laboratory tests	x	x	x
Endocrine tests	x		x
ECG[3]	x		
Evaluation of AEs	x	x	x
Abdominal + thoracic CT scan[4]	x		x
Quality of Life	x		x
Mitotane Monitoring[5]	x	x	x

[1] only in the mitotane arm until mitotane levels plateau

[2] time interval will be increased after 2 years to 6 months and after 5 years to 12 months

[3] ECG will be repeated every 6 months in patients on mitotane

[4] MRI can be performed as alternative

[5] only in the mitotane arm

5.3 Inclusion-Exclusion Criteria and General Study Population

5.3.1 Inclusion criteria

Subjects must satisfy all of the following criteria to be included in the study:

- Histologically confirmed diagnosis of ACC according to Weiss system.
- Low-intermediate risk of recurrence defined as:
  - o Stage I-III (ENSAT staging system).
  - o Microscopically complete resection, defined as no evidence of microscopic residual disease based on surgical reports, histopathology and post-operative imaging.
  - o Ki 67 $\leq$ 10%.

- Post-operative imaging (thoracic and whole abdominal CT with contrast medium or MRI) demonstrating no evidence of disease within 4 weeks before randomization.
- Age $\geq$ 18 years.
- ECOG performance status 0-2.
- Adequate bone marrow reserve (neutrophils $\geq$ 1000/mm3 and/or platelets $\geq$ 80000/ mm3).
- Ability to comply with the protocol procedures.
- Written informed consent.

5.3.2 Exclusion criteria

Subjects who meet any of the following criteria are disqualified from entering the study:

- Time between primary surgery and randomization > 3 months.
- Repeated surgery for recurrence of disease.
- Persistence of autonomous adrenocortical hormone secretion following surgery.
- History of prior malignancy, except for cured non-melanoma skin cancer, cured in situ cervical carcinoma, or other treated malignancies with no evidence of disease for at least three years.
- Renal insufficiency (creatinine clearance < 40 ml/min) or liver insufficiency (serum bilirubin > 2 times the upper normal range and/or serum transaminases (AST/GOT, ALT/GPT) >3 times the upper normal range). Creatinine clearance may be calculated according to validated formulas (Crockoft's or MDRD).
- Pregnancy or breast feeding.
- Previous or current treatment with mitotane or other antineoplastic drugs for ACC.
- Previous radiotherapy for ACC.
- Any other severe acute or chronic medical or psychiatric condition, or laboratory abnormality that would impart, in the judgment of the investigator, excess risk associated with study participation or study drug administration, or which, in the judgment of the investigator, would make the patient inappropriate for entry into this study.

5.4 Randomization

After consent, patients are randomly assigned to receive either adjuvant mitotane or active surveillance only with the use of a 1:1 randomization stratified for stage (I-II vs. III). A

centralized randomization procedure running online and implemented with the electronic CRF at the web-site http://www.adiuvo-trial.org is used throughout the study. The technique of randomly permuted balanced blocks and random block size was used to prepare the 2 random lists, one for stage I-II and the other for stage III. After registering the patient and confirming inclusion and exclusion criteria, the patient is randomized to either mitotane treatment or surveillance only through logging to the web system. The web-based randomization procedure, developed by the Trial Center of the Piedmont Oncology Network, is continuously accessible (24/24h a day). The procedure is concealed to researchers and accessible only to the Clinical Research Coordinator.

5.5 Blinding

The trial is conducted unblinded. A blind placebo-controlled study is unfeasible since patients treated with mitotane are at risk of adrenal insufficiency and need preventive steroid coverage, which may have detrimental consequences in patients who are not treated with mitotane and are not at risk of adrenal insufficiency. Also masking the steroid treatment is unfeasible, due to the need to modulate steroid dosage based on clinical assessment of signs, symptoms and laboratory data, to prevent adrenal insufficiency.

6 Sample Size

The sample size was calculated using the primary endpoint RFS. Based on the results of previous studies and data from the German ACC Registry, in patients with low recurrence risk a RFS rate after 2 years is estimated to be about 0.60 with surgery only. The sample size was calculated to provide an 80% chance of detecting an increase in the probability of being alive and free from recurrence at 2 years from 0.60 to 0.75. According to O'Brien and Fleming sequential design with maximum three stages, setting 0.05 as the level of significance (alpha) and using a two-sided log rank test for analysis, it was calculated that 97 events in the primary endpoint RFS should be observed and approximately 184 patients should be randomized considering 4 years of accrual and 2 years of follow-up after entry of the last patient. Assuming a lost-to-follow-up rate of maximum 10%, a total of 200 patients (100 per treatment arm) will be needed.

7 General Considerations

7.1 Framework

The trial uses a superiority hypothesis testing framework between treatment groups.

7.2 Interim analyses

The two interim analyses are planned at 20% and 60% of the information rate (that under constant accrual rate correspond to about 2 years observation time and 19 expected events and, 4 years observation time and 58 expected events, respectively). Critical p-values for stopping the study are determined at p = 0.00001 for the first interim analysis, p = 0.01202 for the second, and p = 0.0464 for the final analysis. Interim evaluations aimed at checking data quality and safety issues are planned.

7.3 Planned sample size adjustment

N/A

7.4 Stopping rules

See 7.2 (interim analyses)

7.5 Timing of Analyses

Lock of database is planned after cleaning the database and solving all relevant queries regarding the co-primary endpoints. The final analysis will be conducted when all data will be collected. The final analysis will be performed on data transferred to the file “FAP-Final”, having been documented as meeting the cleaning and approval requirements and after the finalization and approval of this SAP document.

7.6 Analysis Populations

7.6.1 Intention-to-treat Population

All patients randomized into the study. The ITT Analysis population will be the primary analysis set for all efficacy analyses. Subjects will be analyzed according to the treatments assigned at randomization.

7.6.2 Per Protocol Population

All subjects who did not substantially deviate from the protocol as to be determined on a per-subject immediately before data base lock. This is defined by taking at least one tablet of mitotane in the mitotane group and no mitotane at all in the surveillance group.

7.6.3 Safety Population

All randomized patients who received at least 1 dose of study treatment (including controls). Subjects will be summarized according to treatment actually received.

7.7 Covariates and Subgroups

Prespecified subgroup analyses will be done to look at the consistency of treatment effect according to age, sex, tumor size, stage, Ki67, and secretion using a set of Cox's proportional hazard models that include an interaction term between the treatment assignment and the subgroup of interest, each at a time. The following subgroup analyses will be determined using the baseline data:

Age (<50; ≥50)
Sex (male, female)
Tumor stage (stage 1-2; stage 3)
Tumor size (< 10mm; ≥10)
Ki67 (≤ 5; >5)
Secretion (yes, no).

7.8 Missing Data

There is no planned data imputation for missing data in this study. Every effort will be made to collect the data by querying study sites.

8 Summary of Study Data

Statistical analysis and generation of tables, figures, subject data listings, and statistical output will be performed using R Version 4·0·2 and SAS version 9·4 (SAS Institute, Cary, NC).

Descriptive statistics will be displayed to provide an overview of the study results. For continuous variables, descriptive statistics will include number of subjects with available measurements (M), median, and interquartile range (IQR). For categorical variables, the number and percentage of subjects in each category will be reported. Unless otherwise stated, the denominator for percentages will be based on the number of subjects included in the respective analysis set.

8.1 Demographic and Baseline Variables

SECTION	Variable code	Label	Unit of measurement	Option 1	Option 2	Option 3
1. Demographic data	1.1	Age	years			
	1.2	Sex		M	F	
	1.3	Race/ethnicity		Caucasian	Other	
2. Pathological examination	2.1	Size of tumor	mm			
	2.2	Weiss score				
	2.3	Ki67 assessment	%			
	2.4	Tumor stage		I	II	III
3. Surgical report	3.1	Surgical procedure		Open	Laparoscopy	
4. Endocrine assessment	4.1	Preoperative autonomous hormone secretion		Yes	No	
	4.2	Clinically apparent Cushing phenotype		Yes	No	
5. Quality of life	5.1	EORTC-QoL-C30 Functional scale				
	5.2	EORTC-QoL-C30 Symptoms scale				
	5.3	EORTC-QoL-C30 Global health scale				

8.2 Efficacy Analysis

Data will be summarized by treatment group. N, Median, I and III quartiles will summarize continuous variables, whereas number and percent will summarize categorical variables.

The primary analysis on RFS will be conducted as follows: for each treatment group, the RFS distribution and the median RFS time will be estimated using the Kaplan-Meier method. The two-sided logrank test will be used to compare the survival times between the two arms. The Cox's proportional hazard model will be performed to estimate the HR with 95% confidence intervals. To assess the proportional hazard assumption both graphical checks and statistical tests (as introducing into the model an interaction term between the treatment variable and a function of time) will be applied. The stratification employed to randomize the patients should prevent unbalances between the two arms, however an adjusted HR, standardized for the most important prognostic factors, will also be estimated.

The primary analysis will be based on the ITT analysis set. However, a sensitivity analysis will be conducted on a per protocol analysis set. The latter only serves to confirm the robustness of the results.

Further analyses of the secondary endpoints are sensitivity analyses, or they are descriptive or explorative. Subgroup analyses will be performed by including in the Cox model interaction terms between the treatment and subgroup variables.

Further analyses of the secondary endpoints are sensitivity analyses, or they are descriptive or explorative. Subgroup analyses will be performed by including in the Cox model interaction terms between the treatment and subgroup variables.

Survival curves will be plotted to present data on RFS and OS, while baseline characteristics will be presented separately for both study arms with descriptive statistics.

8.2.1 Safety Analyses

Data will be summarized by treatment group. Number and percent will summarize categorical variables.

9 Reporting Conventions

P-values ≥0.001 will be reported to 3 decimal places; p-values less than 0.001 will be reported as “<0.001”.

The mean, standard deviation, and any other statistics other than quantiles, will be reported to one decimal place greater than the original data.

Quantiles, such as median, or minimum and maximum will use the same number of decimal places as the original data.

Estimated parameters, not on the same scale as raw observations (e.g. regression coefficients) will be reported to 3 significant figures.

10 Technical Details

A second review statistician will independently reproduce the primary analyses and summary statistics tables related to the main efficacy analysis. The reviewing statistician will have an overview of the entire analyses and will explicitly check the code producing the afore mentioned tables as well as any other pieces of code as desired.

11 Summary of Changes to the Protocol

On December 2018, only 91 patients had been enrolled in the randomized ADIUVO trial and the Steering Committee decided to stop the patient enrolment. Thus, the planned interim analyses will not be carried out. The decision to stop the study was not based on any stopping rules mentioned in the study protocol, but considering that the recruitment was too slow to achieve the required number of patients in an acceptable time frame.
